# North and South: Exploring isotopic analysis of bone carbonates and collagen to understand post‐medieval diets in London and northern England

**DOI:** 10.1002/ajpa.24818

**Published:** 2023-07-22

**Authors:** Blessing Chidimuro, Sean Doherty, Jonathan Finch, Paola Ponce, Jack Eggington, Sarah Delaney, Camilla Speller, Matthew J. Collins, Malin Holst, Michelle Alexander

**Affiliations:** ^1^ Department of Geography and Environmental Science University of Reading Reading UK; ^2^ Department of Archaeology University of York York UK; ^3^ Department of Archaeology University of Exeter Exeter UK; ^4^ York Osteoarchaeology Ltd York UK; ^5^ Department of Anthropology University of British Columbia Vancouver Canada; ^6^ Department of Archaeology University of Cambridge Cambridge UK; ^7^ Natural History Museum, University of Copenhagen Copenhagen Denmark

**Keywords:** carbon, carbonate, diet, nitrogen, post‐medieval

## Abstract

**Objectives:**

We evaluate the potential of paired isotopic analysis of bone carbonate and collagen to examine the diet of post‐medieval human and animal populations from England (17th–19th c.), including, for the first time, manufacturing towns in northern England. The potential for identifying C_4_ crop consumption is explored alongside regional and local patterning in diet by sex and socioeconomic status.

**Materials and Methods:**

Humans (*n* = 216) and animals (*n* = 168) were analyzed from sites in London and northern England for both carbon and nitrogen isotopes of bone collagen (𝛿^13^C_coll_, 𝛿^15^N_coll_). Isotopic analysis of bone carbonates (𝛿^13^C_carb_, 𝛿^18^O_carb_) was carried out on all humans and 27 animals, using Fourier transform infrared spectroscopy–attenuated total reflectance to assess diagenesis.

**Results:**

Variations in diet were observed between and within different populations by geographical location and socioeconomic status. Three pigs and one cow consumed C_4_ resources, indicating the availability of C_4_‐fed animal protein. Londoners consumed more animal and marine protein and C_4_ resources. Middle‐ and upper‐class populations from both London and northern populations also had greater access to these foods compared to those of lower status in the same regions.

**Discussion:**

This substantial multi‐isotope dataset deriving from bone carbonate and collagen combined from diverse post‐medieval urban communities enabled, for the first time, the biomolecular identification of the dynamics of C_4_ consumption (cane sugar/maize) in England, providing insight into the dynamics of food globalization during this period. We also add substantially to the animal dataset for post‐medieval England, providing further insight into animal management during a key moment of agricultural change.

## Graphical abstract

Cane sugar consumption in post‐medieval individuals.

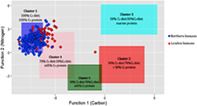

## 1 INTRODUCTION

The analysis of stable carbon isotopes in bone carbonate (𝛿^13^C_carb_) has been routinely employed to examine the adoption and spread of maize, a C_4_ plant, across the Americas (Tykot, 2006; Tykot et al., 1996). In northwest Europe, however, where C_3_ plants (e.g., wheat, barley, oats), predominate as dietary staples throughout the archeological record, isotope analysis of bone carbonate has rarely been applied. This study focuses on a period that saw the addition of C_4_ crops (cane sugar and maize) to human and animal diets in 17th–19th century England, a significant phase of food globalization.

The 17th–19th century in England witnessed unprecedented changes in almost all aspects of daily life, including dramatic shifts in food acquisition and consumption across both rural and urban environments linked to the agricultural and industrial revolutions, although the role of these revolutions in the transformations is hotly debated (Burnett, 2005; Crafts, 1985; Overton, 1996). Agricultural accounts reveal huge increases in arable output, as well as improvements in animal husbandry that sustained an increasingly urbanized population during this period (Clayton & Rowbotham, 2008; Turner et al., 2001). The political and social systems in England also had to contend with numerous wars during this period (e.g., the outbreak of the French wars in 1793) that frequently resulted in food shortages. With the expanding population, growth in urban or industrial regions, significantly in London and the northern manufacturing towns, increased competition and social imitation among different classes leading to differences in purchasing power and eating habits, as well as distinctive tastes across all classes (Burnett, 2005; Shammas, 1984). Dietary surveys conducted in the 19th century by Dr Edward Smith revealed marked regional differences in diet (Barker et al., 1970; Smith, 1864), (see Data S3 for more information on post‐medieval diet). In this article, we investigate these differences through isotopic analysis of 11 populations of differing socioeconomic statuses from cities in northern England, as well as London, to explore structured relations and dietary trends in 17th–19th century industrial England.

### 1.1 C_4_ input in diet

While crops grown in early‐modern England were predominantly C_3_, this period saw the opening up of the New World and a dynamic period of food globalization, resulting in C_4_ species such as sugar cane and maize being imported from the Americas (Mintz, 1986; Thirsk, 2007). Maize consumption developed following the Great Famine in Ireland (1845–1849) when it was used as relief food for the Irish who emigrated to England (Kinealy, 2006), though it was not until the early‐20th century that maize was widely accepted by most for human rather than animal consumption (Hill, 1977; Holland, 1919). Cane sugar consumption grew with Britain's colonization of the West Indies in the 17th century, which led to the average intake per capita increasing ~12 kg/year between 1700 and 1850, and further still following the abolition of sugar tax in 1874 (Johnson et al., 2007; Mintz, 1986; Walvin, 2017). This increase is evidenced by the prevalence of dental caries and antemortem tooth loss during this period; both consistent with the consumption of a diet high in refined sugars and processed carbohydrates (Mant & Roberts, 2015). With the British consuming more cane sugar than other Western European countries, and the Irish immigrants who had consumed maize migrating to the country around that time, this makes Britain the ideal place to study the consumption of cane sugar and/or maize in the 18th and 19th centuries.

### 1.2 Isotopic approaches to post‐medieval diets

This article uses multiple fractions of bone—both collagen and mineral—for isotopic analysis. The mechanisms underlying the fractionation of stable isotopes within these tissue components and different ecosystems have been outlined in detail elsewhere (for a detailed review see Schwarcz & Schoeninger, 2012). Carbon isotope values differentiate C_3_ and C_4_ plant foods with C_3_ plants (e.g., wheat and barley) possessing lower 𝛿^13^C values than those of C_4_ plants (e.g., maize and cane sugar). Nitrogen isotope values vary with trophic level, with 𝛿^15^N values rising by ~3‰–5‰ with each trophic step (Bocherens & Drucker, 2003). While the 𝛿^13^C ranges in marine and C_4_ foods overlap, nitrogen isotopes can also be used as an indicator of marine dietary sources because the longer trophic chains common in aquatic ecosystems result in higher 𝛿^15^N values (Chisholm et al., 1982; Schoeninger & DeNiro, 1984).

Due to turnover, bone tissues represent an individual's average diet over a number of years prior to death, depending on the skeletal element (Hedges et al., 2007). Bone collagen 𝛿^15^N values provide information on the main protein sources of the diet. While bone collagen 𝛿^13^C values (𝛿^13^C_coll_) also mostly represent dietary protein, about 25% of bone collagen (non‐essential amino acids) is typically synthesized from other dietary sources (lipids/carbohydrates) (Fernandes et al., 2012). Bone carbonate 𝛿^13^C values (𝛿^13^C_carb_), however, reflect whole diets (carbohydrates, lipids, and proteins) because bone carbonate forms in equilibrium with blood carbonate that is itself a product of energy metabolism (Ambrose & Norr, 1993; Krueger & Sullivan, 1984). Bone 𝛿^13^C_carb_ values can reveal C_4_ contributions from the whole diet that may be obscured when using bone 𝛿^13^C_coll_ alone. The utilization of both 𝛿^13^C_coll_ and 𝛿^13^C_carb_ in isotopic studies, therefore, provides a more holistic estimate of several dietary inputs. In Europe, some studies have made use of both components (Dotsika et al., 2022; Etu‐Sihvola et al., 2022; Grupe et al., 2009; Gugora et al., 2022; Olsen et al., 2016; Reitsema et al., 2010; Toyne et al., 2022; Yoder, 2012; Zechini et al., 2021). Although 𝛿^13^C_coll_ data from these studies suggested that dietary protein was primarily derived from C_3_ resources, the differences in the 𝛿^13^C_carb_ values among the individuals at certain sites indicated that some individuals may have consumed more C_4_ or marine foods than others (Dotsika et al., 2022; Reitsema et al., 2010; Yoder, 2012), demonstrating the utility of analyzing 𝛿^13^C_carb_ alongside 𝛿^13^C_coll_ values.

Previous studies of post‐medieval diet in England have all focused on the utilization of 𝛿^13^C_coll_ and 𝛿^15^N and the majority of studies have been conducted on populations from London, with only a few from elsewhere (i.e., Birmingham [Richards, 2006], Coventry [Trickett, 2006], Plymouth and Gosport [Roberts et al., 2012] and Chichester [Dhaliwal et al., 2020]). These studies have focused on exploring the diets of different social classes, including the lower‐class (Beaumont, 2013; Beaumont et al., 2013a, 2013b; Trickett, 2006), mixed (Bleasdale et al., 2019; Dhaliwal et al., 2020), and upper‐class diets (Bleasdale et al., 2019; Brown & Alexander, 2016; Nitsch et al., 2010, 2011; Richards, 2006; Trickett, 2006), the diet of naval seamen (Roberts et al., 2012) and the dietary signatures of migration and famine (Beaumont, 2013; Beaumont et al., 2013a, 2013b). All these studies have alluded to the potential for some inclusion of C_4_ resources such as maize or sugar in the diets of some individuals at each of their respective sites, visible as slightly higher 𝛿^13^C_coll_ values. Nevertheless, C_4_ sources cannot be decoupled from marine sources, when using bulk bone collagen isotopes alone, particularly where 𝛿^15^N values are also high. Bone carbonates may provide greater clarity as to the presence of C_4_ carbohydrates in the diet. The present study represents the first application of carbon isotope analysis of bone carbonate (𝛿^13^C_carb_) to skeletal remains from England alongside isotopic analysis of bone collagen (𝛿^13^C_coll_, 𝛿^15^N).

## 2 MATERIALS AND METHODS

### 2.1 Sites

**FIGURE 1:**
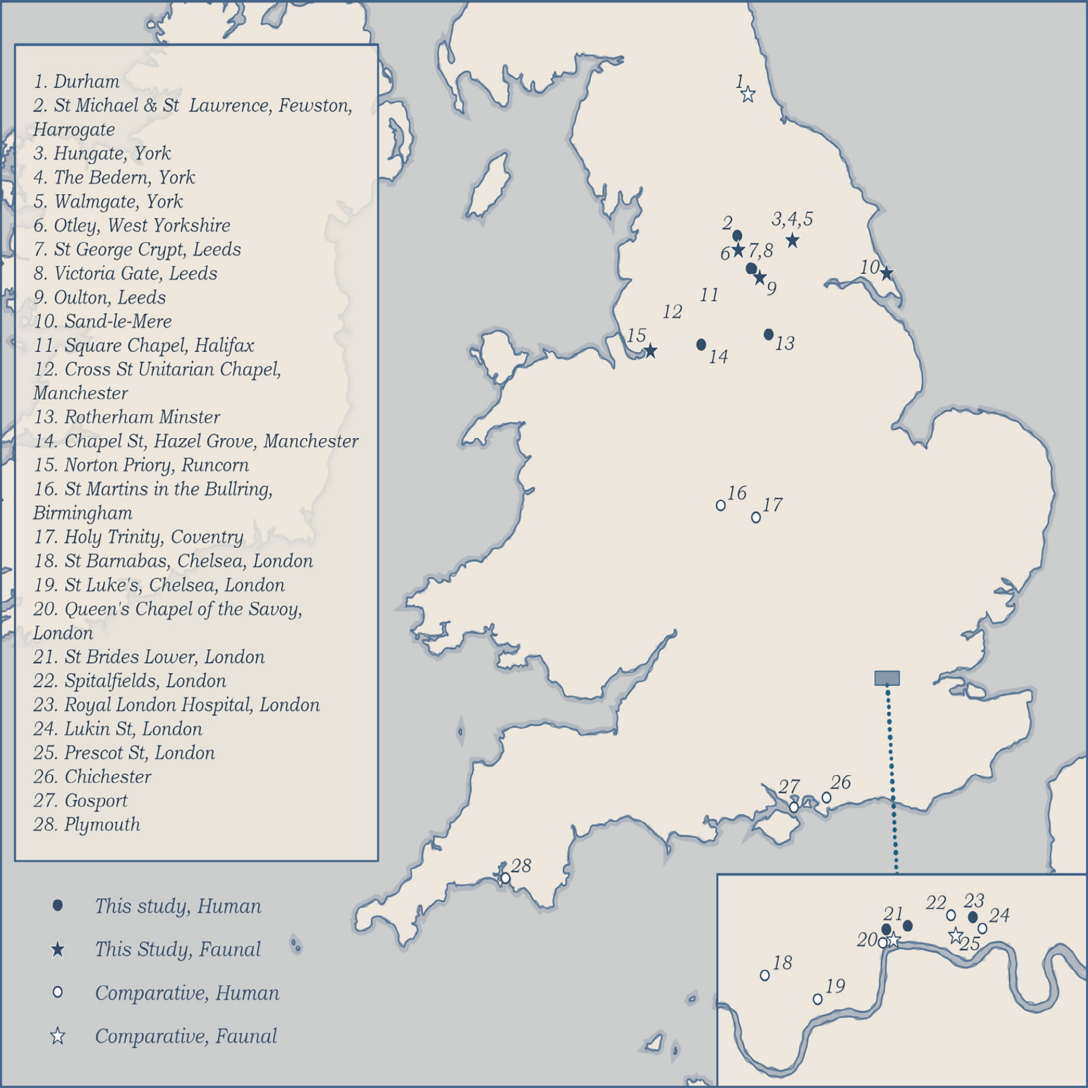
Map of post‐medieval England showing the locations of the sites of study and other post‐medieval sites mentioned in the text. The city of London is shaded, and sites are given numbers from 18 to 25. (Map by Helen Goodchild, Department of Archaeology, University of York).

We analyzed a total of 385 samples, comprising 216 human bone and 169 animal bone samples. Human bone samples (194 adults and 22 non‐adults) derive from 11 sites: Four from London and seven from manufacturing towns from Northern England (Figure 1). All non‐adults (aged 0–17 years) are from Cross Street and Hazel Grove. Altogether, these populations date from the late‐16th to 19th centuries; however, most date to the 18th and 19th centuries (Table 1). The samples cover a range of social status groups and geographical locations to assess the extent, if any, of variation in diet and socio‐economic status. Detailed information on the historical context of each human population is provided in Data S1 and a brief description is provided in Table 1. Contemporary animal material was sampled from nine sites that were located close to the human cemeteries to create an isotopic baseline (Table 2). Under current legislation, formal ethical approval is not required, as these remains are not covered by the Human Tissues Act (2004) or similar legislation, as they are over 100 years old. Bone collagen for all 169 animals was analyzed for 𝛿^13^C_coll_ and 𝛿^15^N. Bone carbonate 𝛿^13^C_carb_ was analyzed for all 216 human individuals and a sample of 27 animals. Bone collagen data for 70 of the 216 humans analyzed for carbonates were obtained from Bleasdale et al. (2019), Chidimuro et al. (n.d.), and Gowland et al. (2023).

**TABLE 1:** Humans sampled for bone collagen and bone carbonate analysis from all sites, with associated dates and economic status.

Region	Location	Site name	Status	Period	Denomination	Osteological reference
Northern towns	Manchester	Cross Street Unitarian Chapel (*n* = 54)	Middle/upper class	1694–1852	Non‐conformist Unitarian	(Keefe & Holst, 2017)
	Stockport	Chapel St, Hazel Grove (*n* = 31)	Mixed middle/low	1794–1910	Non‐conformist Wesleyan	(Newman & Holst, 2016)
	Fewston	St Michael & St Lawrence, Fewston (*n* = 7)	Mixed^a^	18th–19th c.	Church of England	(Caffell & Holst, 2017; Gowland et al., 2023)
	Halifax	Square Chapel (*n* = 32)	Middle class	1772–1857	Non‐conformist Congregationalist	(Keefe & Holst, 2015)
	Leeds	St George's Crypt (*n* = 9)	Middle/upper class	1840–1911	Anglican Church	(Caffell & Holst, 2009)
	Leeds	Victoria Gate (*n* = 3)	Low status	1796–1850s	Non‐conformist Methodist	(Caffell & Holst, 2014)
	Rotherham	Rotherham Minster (*n* = 21)	Low status	1780s–1854	Church of England	(Keefe & Holst, 2011)
London	London	Queen's Chapel of the Savoy (*n* = 10)	Mixed	1510–1854	Unknown^b^	(Sibun & Ponce, 2018)
	London	Barnabas/St Mary Abbots, Kensington (*n* = 23)	Upper class	18th–19th c	Church of England	(Bleasdale et al., 2019)
	London	Royal London Hospital (*n* = 11)	Low status	1825–1841	Mixed	(Fowler & Powers, 2012)
	London	St Brides Lower (*n* = 15)	Low status	1770–1849	Church of England	(Miles & Conheeney, 2005)

^a^ The Fewston assemblage includes rural farmers and industrial mill workers. All seven individuals date between the 18th and 19th centuries.

^b^ Difficult to confirm as there would have been people buried from many religious backgrounds, however, they are more likely non‐denominational or nominally Anglican.

**TABLE 2:** Faunal remains sampled for bone collagen and bone carbonate analysis.

Region	Site name	Number of samples for bone δ^13^C_coll_	Number of samples for bone δ^13^C_carb_	Location	Site code	Period
Northern towns	Cross Street (*n* = 37)	37	12	Manchester	CSM	17th–19th c.
	Norton Priory (*n* = 75)	75	6	Norton Runcorn	NP	16th–20th c.
	Hungate (*n* = 21)	21	0	York	HUN	16th–20th c.
	The Bedern (*n* = 11)	11	0	York	BED	15th–19th c.
	Walmgate (*n* = 3)	3	0	York	WAL	18th–19th c.
	Oulton (*n* = 4)	4	0	Leeds	Flee	Post–Medieval^a^
	Otley (*n* = 8)	8	3	Otley	GPMO	Post–Medieval^a^
	Sand‐le‐Mere (*n* = 3)	3	0	Sand‐le‐Mere, near Hull	SALM T8	17th–19th c.
	Square Chapel (*n* = 7)	7	6	Halifax	SQC	18th–19th c.
	Total	169	27			

*Note*: Animals from Cross Street and Square Chapel Halifax were sampled from the same location as the human cemeteries.

^a^ No exact date.

### 2.2 Methods

Detailed procedures for collagen and carbonate extraction and analysis can be found in Data S2. Sample preservation was assessed prior to the analysis of bone carbonate through Fourier transform infrared spectroscopy–attenuated total reflectance (FTIR‐ATR), taking into account the presence of calcite, the infrared splitting factor (IRSF) (Hollund et al., 2013; Sivakumar et al., 2014; Weiner & Bar‐Yosef, 1990) and carbonate‐to‐phosphate ratio (C/P), (Wright & Schwarcz, 1996). FTIR‐ATR sample preparation and analysis were executed according to the method of Kontopoulos et al. (2018). Collagen extraction of bone followed the procedure outlined in Longin (1971) and Brown et al. (1988). Collagen yield, %C, %N, and C/N ratios were utilized to assess collagen preservation (Ambrose, 1990; DeNiro, 1985; Sealy et al., 2014). Carbonate extraction of bone samples was adapted from Snoeck and Pellegrini (2015) and Pellegrini and Snoeck (2016).

To explore dietary patterns integrating bone collagen (𝛿^13^C_coll_ and 𝛿^15^N) and carbonate isotope values (𝛿^13^C_carb_), two related modeling procedures were utilized: (i) a simple carbon isotope model and (ii) a multivariate isotope model (Froehle et al., 2010, 2012). These two models were developed to estimate the relative amount of C_3_ and C_4_ foods in the diet as well as to determine whether the protein was derived from C_3_, C_4_ or marine resources. These modeling techniques improve on the bivariate plotting of collagen results (𝛿^13^C_coll_, 𝛿^15^N), which are biased toward dietary protein sources. The inclusion of 𝛿^13^C_carb_ values permits the incorporation of the whole diet in the analysis and provides more detailed paleodiet reconstructions. The multivariate isotope model of Froehle et al. (2012) is based on archeological North American samples but the authors utilized controlled feeding experimental animal data to test their model, verifying that it could also be used for other archeological populations. Bone carbonate models for European populations are currently unavailable, therefore, this study uses the clusters in the model to indicate which individuals probably consumed C_4_ foods in their diet in a relative manner.

All stable isotope ratios are reported using standard delta (𝛿) notation. 𝛿^13^C_collagen_ (𝛿^13^C_coll_), 𝛿^13^C_carbonate_ (𝛿^13^C_carb_), and 𝛿^15^N_collagen_ (𝛿^15^N) values used in the text are expressed in parts per mille (‰) relative to international standards, Vienna PeeDee Belemnite standard and atmospheric nitrogen for 𝛿^13^C and 𝛿^15^N, respectively using the following equation:

$$\delta^{i}E_{\text{sample}}=\frac{\left(i_{E}/j_{E}\right)\text{sample}-\left(i_{E}/j_{E}\right)\text{reference}}{\left(\frac{i_{E}}{j_{E}}\right)\text{reference}.}$$

where ^*i*^ *E* and ^*j*^ *E* denote the heavier and lighter isotopes respectively (Roberts et al., 2018). The total uncertainty for all samples across all runs was <0.2‰ for 𝛿^13^C_carb_, 𝛿^13^C_coll_ and 𝛿^15^N, see Data S2 for full details.

### 2.3 Statistical analysis

Statistical analysis and data visualization were carried out using R (R Core Team, 2020), PAST (Hammer et al., 2001) and IBM SPSS statistics version 26 (IBM, 2019). Non‐parametric tests (Mann Whitney and Kruskal Wallis for pairwise and multiple comparisons, respectively) were used to compare isotope values among groups due to the non‐normal distribution of data as indicated by Kolmogorov–Smirnov and Shapiro–Wilk tests. Post hoc pairwise tests adjusted using Holm's Sequential Bonferroni correction were used following Kruskal Wallis comparisons.

## 3 RESULTS

The 𝛿^13^C_coll_, 𝛿^13^C_carb_, 𝛿^18^O_carb_ and 𝛿^15^N_coll_ isotope data for humans and animals from all sites are presented in Data S4, Tables S1 and S2. The IRSF and C/P ratios are listed in Data S4, Table S3. There are no statistically significant differences in the 𝛿^13^C_coll_, 𝛿^13^C_carb_ or 𝛿^15^N_coll_ values between males and females at any site that had a sufficient number of individuals sexed (*n* = ≥15, Data S4, Table S4), therefore, all individuals of both sexes are combined for each site in the evaluation of geographical and status differences.

### 3.1 Sample preservation

All samples produced collagen yields of ≥1% and C:N ratios indicative of well‐preserved collagen (Ambrose, 1990; DeNiro, 1985; Sealy et al., 2014). Good bone carbonate preservation was indicated by crystallinity (IRSF) values (human mean = 3.72 ± 0.18) between 3.50 and 3.97 (animal mean = 3.71 ± 0.12), and carbonate content C/P values (human mean = 0.17 ± 0.03) between 0.12 and 0.19 (animal mean = 0.16 ± 0.02). Samples with IRSF values >4.2 (*n* = 6) were excluded from further analysis due to the likelihood of poor bioapatite preservation (Kontopoulos et al., 2020). All except for six samples fell below the mean C/P values found in modern unaltered bone (mean C/P = 0.24 ± 0.003) (Kontopoulos et al., 2019), indicating a loss of the carbonate fraction in the bone apatite. The six samples for which the C/P values fell above the modern bone range were retained within the overall analysis because they were very close to the “normal” range. Additionally, their carbonate content is reduced as a result of pre‐treatment. We identified a peak at 712 cm^−1^, which indicates the presence of secondary calcite contaminants (Baxter et al., 1966) in nine (~4%) human samples. However, FTIR spectra do not show a peak after chemical pre‐treatment indicating that no calcite remained in the treated samples, therefore, these samples were included in the bone carbonate isotope analysis. No correlation was observed between 𝛿^13^C and 𝛿^18^O values in either human or animal samples (Figures S1 and S2) suggesting no effect of diagenesis on the isotopic signals (see Data S3).

### 3.2 Animal bone collagen data

**FIGURE 2:**
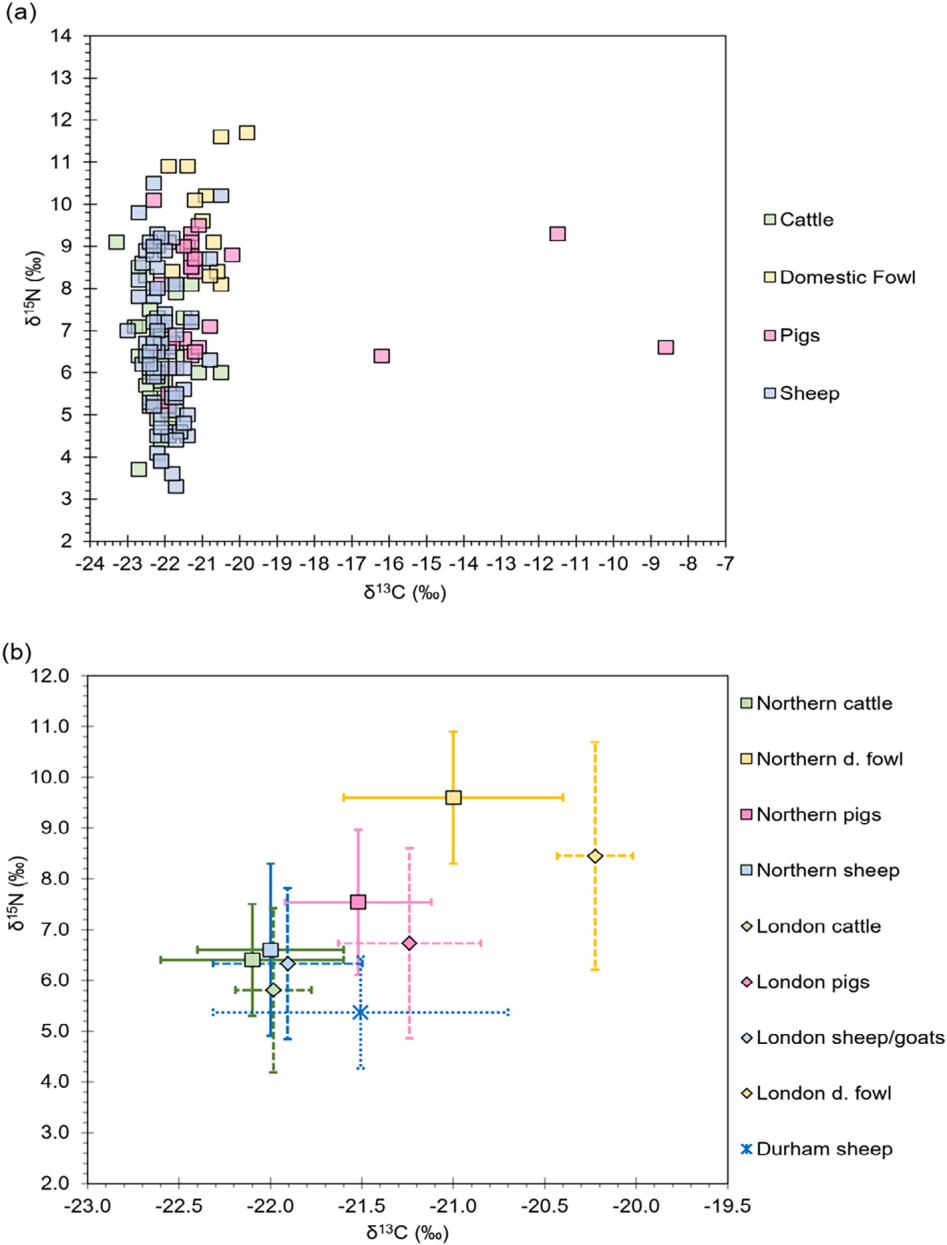
Biplots of 𝛿^13^C and 𝛿^15^N values showing (a) all animals from the northern populations in this study (b) mean and 1σ for animals from the northern towns, London and Durham. Dotted error bars are for reference from comparative data collected in Bleasdale et al. (2019) at Queen's Chapel of the Savoy and Prescot Street sites in London and in Millard et al. (2015) at Durham. The mean value for the northern pigs excludes the three pigs with high 𝛿^13^C.

The 𝛿^13^C_coll_ and 𝛿^15^N data for animals are summarized in Table 3 and plotted in Figure 2. With the exception of three pigs, all faunal carbon isotope values are indicative of C_3_ plant‐based diets (Figure 2a). The mean 𝛿^13^C_coll_ and 𝛿^15^N values from cattle (𝛿^13^C_coll_ = −22.1 ± 0.5‰; 𝛿^15^N = 6.5 ± 1.1‰), pigs (𝛿^13^C_coll_ = −20.6 ± 3.0‰; 𝛿^15^N = 7.5 ± 1.4‰), and sheep (𝛿^13^C_coll_ = −22.0 ± 0.4‰; 𝛿^15^N = 6.6 ± 1.7‰) from the northern sites all fall within the range reported from contemporary sites in London (Bleasdale et al., 2019), though the mean 𝛿^13^C_coll_ and 𝛿^15^N values from sheep in this study are significantly different to contemporary sheep from Durham (Figure 2b) [𝛿^13^C_coll_ Mann–Whitney *U* test *U* = 338.000, *p* = 0.011; 𝛿^15^N Mann–Whitney *U* test *U* = 321.000 *p* = 0.007]. Although there is no statistically significant difference in the 𝛿^15^N values between the northern and London domestic fowls, there is a significant difference in the 𝛿^13^C_coll_ values [Domestic Fowl − (𝛿^13^C_coll_ Mann–Whitney *U* test *U* = 5.000, *p* = 0.03; 𝛿^15^N Mann–Whitney *U* test *U* = 25.000 *p* = 0.391)].

**TABLE 3:** Descriptive statistics for all faunal remains analyzed in this study.

	δ^13^C_coll_ (‰)					δ^15^N_coll_ (‰)				δ^13^C_carb_ (‰)				
Site	*N*	Min	Max	Mean ± 1σ	Range	Min	Max	Mean ± 1σ	Range	*N*	Min	Max	Mean ± 1σ	Range
Cross Street Cattle	14	−22.7	−20.5	−22.0 ± 0.6	2.2	5.4	7.5	6.3 ± 0.6	2.1	4	−14	−12.6	−13.6 ± 0.6	1.4
Norton Priory Cattle	20	−22.8	−21.5	−22.2 ± 0.3	1.3	4.9	7.3	6.2 ± 0.8	2.4	‐	‐	‐	‐	‐
Oulton Cattle	4	−23.3	−21.7	−22.6 ± 0.7	1.6	7.9	9.1	8.5 ± 0.5	1.2	‐	‐	‐	‐	‐
Otley Cattle	3	−22.7	−22.1	−22.3 ± 0.3	0.6	4.4	7.3	6.3 ± 1.6	2.9	1	‐	‐	−13.0	‐
Sand‐le‐Mere Cattle	3	−22.2	−21.3	−21.7 ± 0.5	0.9	4.9	8.1	6.6 ± 1.6	3.2	‐	‐	‐	‐	‐
Square Chapel Cattle	2	−22.7	−21.7	−22.2 ± 0.7	1	3.7	8.1	5.9 ± 3.1	4.4	1	‐	‐	−6.8	‐
Cattle (all)	**46**	**−23.3**	**−20.5**	**−22.1 ± 0.5**	**2.8**	**3.7**	**9.1**	**6.5 ± 1.1**	**5.4**	**6**	**−14**	**−6.8**	**−12.3 ± 2.8**	**7.2**
Cross Street Domestic Fowl	12	−21.8	−20.5	−21.0 ± 0.4	1.3	8.1	11.6	9.4 ± 1.1	3.5	‐	‐	‐	‐	‐
Norton Priory Domestic Fowl	2	−21.9	−19.8	−20.9 ± 1.5	2.1	10.9	11.7	11.3 ± 0.6	0.8	‐	‐	‐	‐	‐
Domestic Fowl (all)	**14**	**−21.9**	**−19.8**	**−21.0 ± 0.6**	**2.1**	**8.1**	**11.7**	**9.6 ± 1.3**	**3.6**	‐	‐	‐	‐	‐
Cross Street Pigs	8	−22.3	−16.2	−21.0 ± 2	6.1	5.5	8.8	6.8 ± 5	3.3	6	−16	−13.4	−15.0 ± 1	2.6
Norton Priory Pigs	20	−22.1	−20.8	−21.4 ± 2	1.3	5.1	9.5	7.6 ± 5	4.4	6	−15.9	−14.3	−15.1 ± 1	1.6
Otley Pigs	2	−11.5	−8.6	−10.1 ± 1	2.9	6.6	9.3	8.0 ± 6	2.7	2	−10.3	−9.9	−10.1 ± 1	0.4
Square Chapel Pigs	1	‐	‐	−22.3	‐	‐	‐	10.1	‐	1	‐	‐	−12.5	‐
Pigs (all)	**31**	**−22.3**	**−8.6**	**−20.6 ± 3.0**	**13.7**	**5.1**	**10.1**	**7.5 ± 1.4**	**5**	**15**	**−16**	**−9.9**	**−14.2 ± 2**	**6.1**
Cross Street Sheep	13	−22.4	−21.4	−22.0 ± 0.4	1	3.3	6.8	5.4 ± 1.0	3.5	2	−15	−14.7	−14.9 ± 0.2	0.3
Norton Priory Sheep	22	−22.7	−21.3	−22.1 ± 0.4	1.4	4.6	10.5	7.9 ± 1.4	5.9	‐	‐	‐	‐	‐
Hungate Sheep	21	−23	−21.5	−22.2 ± 0.4	1.5	3.6	9.2	6.2 ± 1.6	5.6	‐	‐	‐	‐	‐
The Bedern Sheep	11	−22.3	−20.8	−21.9 ± 0.5	1.5	3.9	9	6.4 ± 1.9	5.1	‐	‐	‐	‐	‐
Walmgate Sheep	3	−21.7	−20.8	−21.4 ± 0.5	0.9	4.4	6.3	5.4 ± 1.0	1.9	‐	‐	‐	‐	‐
Otley Sheep	3	−22.4	−21.7	−22.1 ± 0.4	0.7	5.5	6.7	6.2 ± 0.6	1.2	‐	‐	‐	‐	‐
Square Chapel Sheep	4	−22.7	−20.5	−21.8 ± 1.0	2.2	6.2	10.2	8.1 ± 1.6	4	4	−15.3	−12.8	−13.7 ± 1.1	2.5
Sheep (all)	**78**	**−23**	**−20.5**	**−22.0 ± 0.4**	**2.5**	**3.3**	**10.5**	**6.6 ± 1.7**	**7.2**	**6**	**−15.3**	**−12.8**	**−14.1 ± 1.1**	**2.5**

### 3.3 Human bone collagen data

**FIGURE 3:**
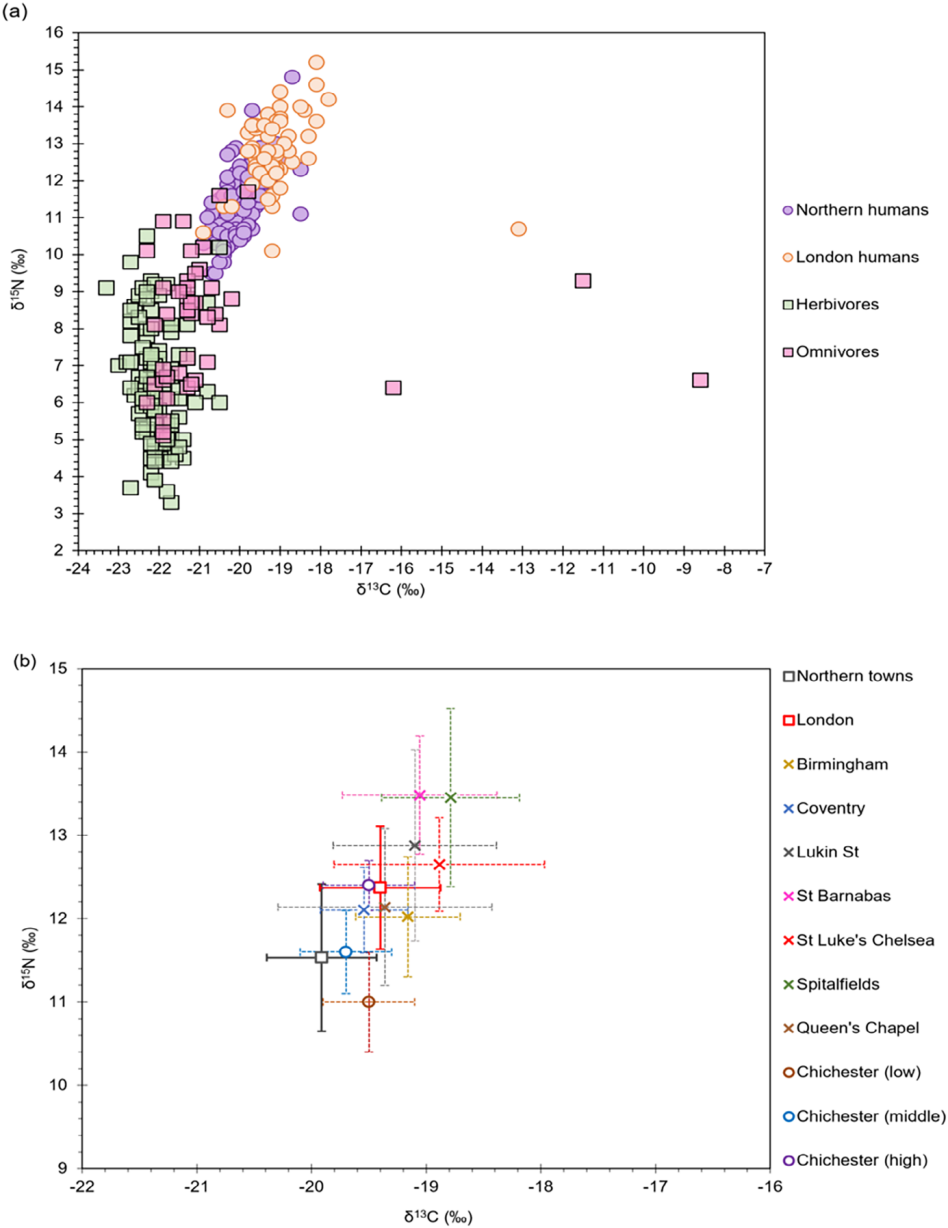
Biplots of 𝛿^13^C and 𝛿^15^N values showing (a) northern adults (*n* = 135) and London adults (*n* = 59) populations plotted together with omnivores (pigs and domestic fowl *n* = 45) and herbivores (sheep and cattle *n* = 124) and (b) showing mean and 1σ for individuals from the northern towns (*n* = 135); and London (St Brides Lower and Royal London only) (*n* = 26) populations and published eight contemporary English sites Chichester (low (*n* = 13), middle (*n* = 14) and high class (*n* = 13)) (Dhaliwal et al., 2020); St Barnabas (*n* = 25) and Queen's Chapel of the Savoy (*n* = 67) (Bleasdale et al., 2019); Lukin Street (*n* = 119) (Beaumont et al., 2013b); Spitalfields, London (*n* = 164) (Nitsch et al., 2010); St Luke's Chelsea (*n* = 32) and Holy Trinity, Coventry (*n* = 13) (Trickett, 2006) and Churchyard of St. Martin's‐in‐the‐Bull Ring, Birmingham (*n* = 18) (Richards, 2006).

Excluding the 22 non‐adults, the London adult populations generally have more elevated 𝛿^13^C_coll_, and 𝛿^15^N values than the northern populations (Table 4; Figure 3a) and this is also supported statistically [𝛿^13^C_coll_ Mann–Whitney *U* test *U* = 1182.5, *p* < 0.05; 𝛿^15^N Mann–Whitney *U* test *U* = 1272.5 *p* < 0.05]. As expected, the human 𝛿^13^C_coll_ and 𝛿^15^N values are closer to the omnivores than the herbivores (Figure 3a). The 𝛿^13^C_coll_ offsets of humans against omnivores for both the northern and London populations (𝛿^13^C_North‐omnivores_ = 0.8‰ and 𝛿^13^C_London‐omnivores_ = 1.8‰) are within the range of trophic level shift of 0‰–2‰ (Bocherens & Drucker, 2003; Lee‐Thorp et al., 1989), and just above the upper limit for herbivores (𝛿^13^C_North‐herbivores_ = 2.2‰ and 𝛿^13^C_London‐herbivores_ = 2.9‰). Regarding the 𝛿^15^N human‐fauna offsets, the offsets between the northern populations and herbivores (5.0 ‰) and omnivores (3.4‰) fall within the expected trophic level enrichment of 3%–5‰ (Bocherens & Drucker, 2003; Schoeninger, 1985) but those from London fall outside the upper limit of 5‰ (𝛿^15^N_London‐herbivores_ = 6.7‰ and 𝛿^15^N_London‐omnivores_ = 5.5‰) reflecting the addition of other resources such as omnivores and marine/freshwater sources.

**TABLE 4:** Summarized statistics of all stable isotope ratios according to region and sites.

	δ^13^C_coll_ (‰)					δ^15^N_coll_ (‰)				δ^13^C_carb_ (‰)			
Site	*N*	Min	Max	Mean ± 1σ	Range	Min	Max	Mean ± 1σ	Range	Min	Max	Mean ± 1σ	Range
Cross Street adults	46	−20.7	−18.5	−19.8 ± 0.5	2.2	9.5	13.0	11.7 ± 0.9	3.5	−16.6	−12.6	−14.5 ± 0.9	4.0
Cross Street non‐adults	8	−20.1	−18.9	−19.3 ± 0.4	1.2	11.5	15.1	13.1 ± 1.6	3.6	−14.7	−10.9	−13.5 ± 1.2	3.8
Hazel Grove	17	−20.6	−19.7	−20.3 ± 0.3	0.9	9.8	11.5	10.7 ± 0.5	1.7	−15.9	−11.3	−14.0 ± 1.2	4.6
Hazel Grove non‐adults	14	−20.7	−19.4	−20.0 ± 0.4	1.3	9.4	13.9	11.4 ± 1.5	4.5	−15.7	−12.9	−14.2 ± 0.8	2.8
Fewston	7	−20.7	−19.3	−20.1 ± 0.5	1.4	10.7	12.2	11.4 ± 0.7	1.5	−16.5	−13.3	−14.6 ± 1.1	3.2
Square Chapel	32	−20.9	−18.7	−19.9 ± 0.5	2.2	10.3	14.8	11.8 ± 0.9	4.5	−16.5	−12.5	−14.8 ± 0.9	4.0
St George Crypt	9	−20.3	−18.5	−19.7 ± 0.5	1.8	11.1	12.4	11.8 ± 0.5	1.3	−15.6	−13.6	−14.6 ± 0.7	2.0
Victoria Gate	3	−20.4	−19.8	−20.1 ± 0.3	0.6	11.6	12.0	11.7 ± 0.2	0.4	−14.9	−13.8	−14.2 ± 0.6	1.1
Rotherham Minster	21	−20.7	−18.8	−19.8 ± 0.4	1.9	10.1	12.8	11.4 ± 0.8	2.7	−16.2	−11.7	−13.6 ± 1.2	4.5
Northern towns (all)	**157**	**−20.9**	**−18.5**	**−19.9 ± 0.5**	**2.4**	**9.4**	**15.1**	**11.6 ± 1.0**	**5.7**	**−16.6**	**−10.9**	**−14.3 ± 1.1**	**5.7**
Queen's Chapel of the Savoy	10	−19.2	−13.1	−18.4 ± 1.9	6.1	10.1	14.4	12.3 ± 1.3	4.3	−15.0	−9.8	−13.7 ± 1.5	5.2
St Barnabas	23	−20.3	−17.8	−19.1 ± 0.7	2.5	12.5	15.2	13.6 ± 0.7	2.7	−15.3	−10.2	−13.6 ± 1.5	5.1
Royal London Hospital	11	−20.4	−18.3	−19.3 ± 0.5	2.1	11.3	13.6	12.5 ± 0.7	2.3	−14.3	−12.3	−13.4 ± 0.7	2.0
St Brides Lower	15	−20.9	−19	−19.5 ± 0.5	1.9	10.6	13.5	12.3 ± 0.8	2.9	−16.0	−12	−14.5 ± 1.0	4.0
London (all)	**59**	**−20.9**	**−13.1**	**−19.1 ± 1.0**	**7.8**	**10.1**	**15.2**	**12.8 ± 1.0**	**5.1**	**−16.0**	**−9.8**	**−13.8 ± 1.3**	**6.2**

*Note*: Stable isotope values reported as the mean ± 1 SD.

When compared with other published populations, we observed that two London populations in this study (St Brides Lower and Royal London Hospital) have lower 𝛿^13^C_coll_, and 𝛿^15^N values relative to others from London, most likely due to their lower socioeconomic status. Additionally, the northern populations have the lowest mean 𝛿^13^C_coll_ values compared to all populations (Figure 3b), although, their 𝛿^15^N values are similar to individuals from middle‐class Chichester.

### 3.4 Human and animal bone carbonate data

All human 𝛿^13^C_carb_ values range from −9.8‰ to −16.6‰, with an average of −14.2 ± 1.2‰. The human 𝛿^13^C_carb_ values have a broader range at each site than the 𝛿^13^C_coll_ which likely reflects the wider range of resources that the carbonate values reflect (Table 4). In scenarios where all individuals' diets are based either entirely on C_3_ resources or entirely on C_4_ resources, the anticipated 𝛿^13^C_carb_ values are −14.5‰ and −0.5‰ respectively (Díaz‐del‐Río et al., 2017; Tykot, 2004). Diets that fall in between, as is the case for almost 40% of the population in this study, suggest mixed C_3_ and C_4_ dietary inputs. The carbonate data suggest some dietary variability, but the populations are mainly dependent on C_3_ terrestrial resources overall.

#### 3.4.1 Regression analysis

**FIGURE 4:**
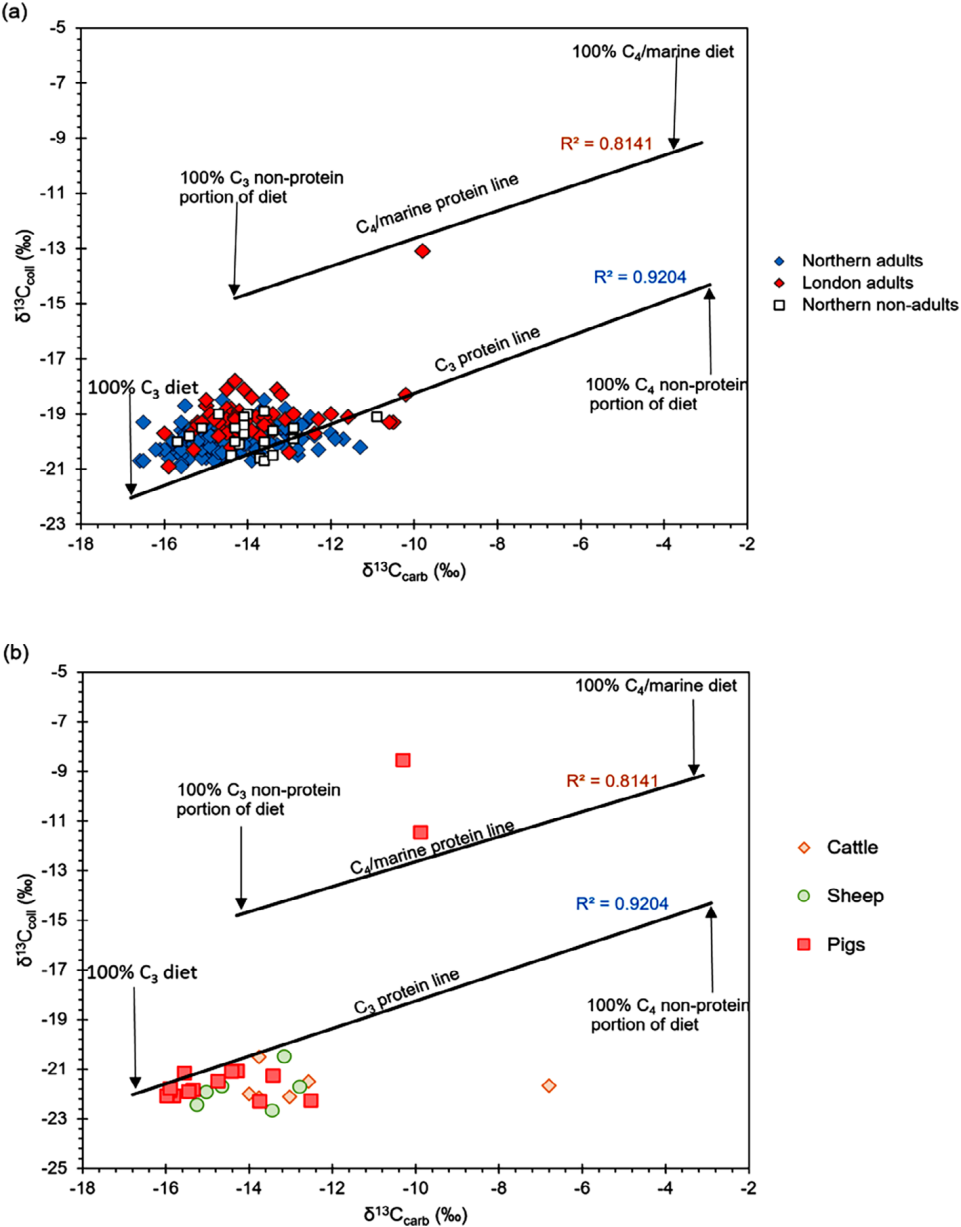
Scatter plot comparing 𝛿^13^C_coll_ and 𝛿^13^C_carb_ values on the Froehle et al. (2010). Bivariate regression model for all (a) humans and (b) animals in this study.

Using both collagen and carbonate data, we plotted the 𝛿^13^C_carb_ and 𝛿^13^C_coll_ values from this study against regression lines of the simple carbon isotope model (Figure 4) developed by Froehle et al. (2010). The proximity of the values to the poles of the regression lines denotes the ratio of C_3_ to C_4_ foods in the diet whereas primary sources of dietary protein are indicated by the proximity of the values to the regression lines.

When the human data are plotted in the regression model, the majority of individuals fall near the C_3_ protein line and the 100% C_3_ diet endpoint suggesting that their main energy contributor (including carbohydrates and fats) was from C_3_ terrestrial resources (Figure 4a). Some London individuals are located away from the 100% C_3_ diet endpoint toward the 100% C_4_ non‐protein portion of the diet suggesting that they may have consumed a small proportion of carbon from C_4_ plants but not C_4_/marine protein sources. It seems that only one London individual (QCS 1123) is displaying a clear inclusion of C_4_/marine protein resources in their diet. Regarding the animals, except for two pigs and a cow that are clear outliers, most animals plot below the C_3_ protein line at the 100% C_3_ energy endpoint suggesting a C_3_ terrestrial diet. The two pigs and the cow consumed C_4_ resources, whereby the pigs consumed C_4_ or marine protein (and possibly plants) whereas the cow only consumed mixture of C_3_ and C_4_ plants (Figure 4b).

#### 3.4.2 Multivariate isotope analysis

**FIGURE 5:**
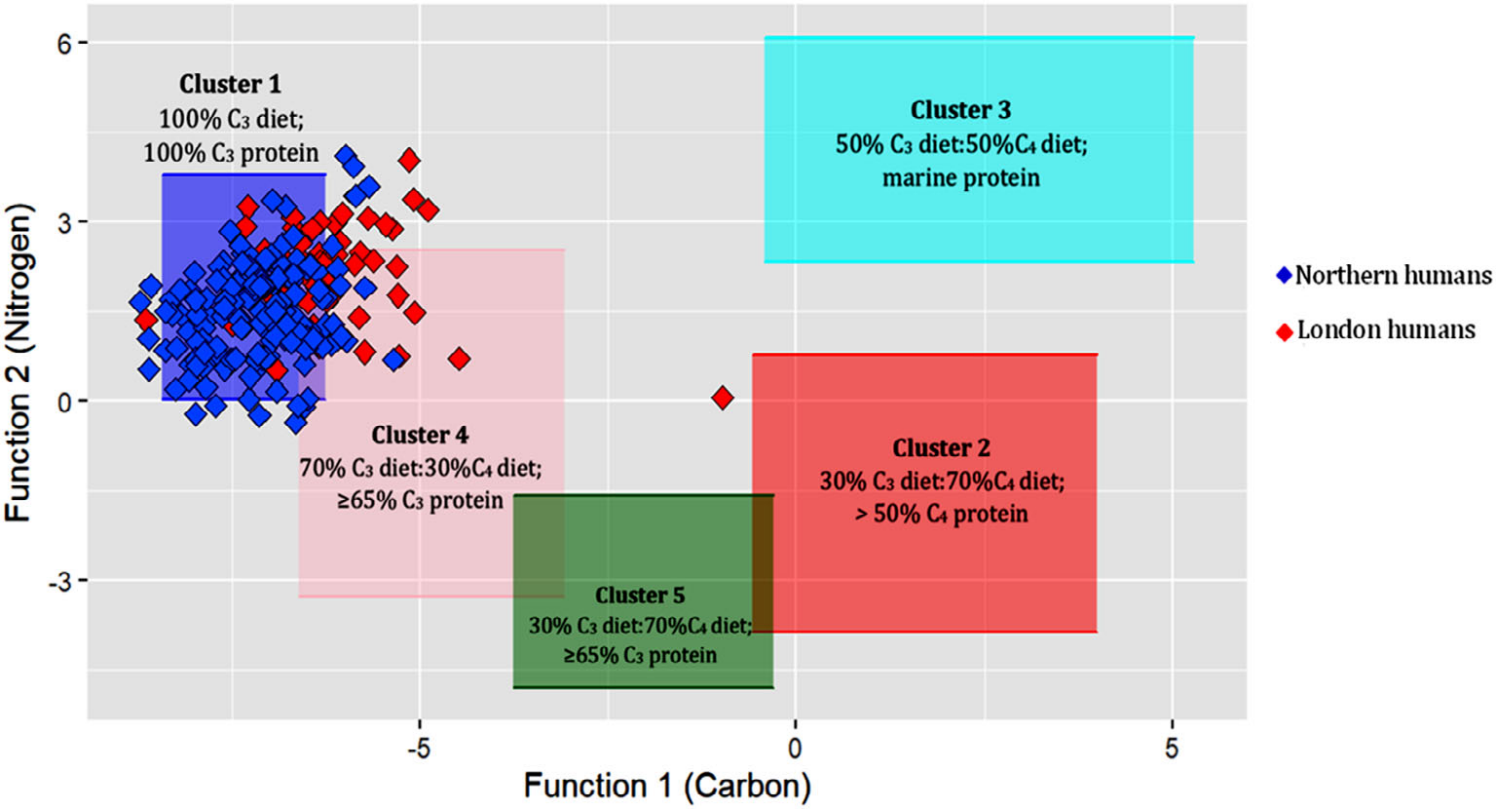
F1 and F2 discriminant function values from northern and London individuals across England plotted against previously generated dietary clusters (see Froehle et al., 2012).

To further explore the data, a multivariate isotope model (Froehle et al., 2012) was employed which incorporates 𝛿^15^N collagen results using cluster and discriminant analysis and enables the plotting of data into five clusters of dietary types (Figure 5). The multivariate model produced results generally in line with the regression analysis model but the addition of the 𝛿^15^N values provided another dimension with which to differentiate between marine and C_4_ diets. When the humans are placed within Froehle et al. (2012) clusters, ~65% of the sampled population, including non‐adults, plot within Cluster 1, indicating a predominantly C_3_ diet (Figure 5). In cases where there are elevated 𝛿^13^C and 𝛿^15^N values (corresponding to Functions 1 and 2 respectively in this case) in children under the age of 2, this may be interpreted to have resulted from breastfeeding (Beaumont et al., 2015), therefore all non‐adults were excluded from the overall interpretation of a C_4_ diet. Around 20% of the population plot within Cluster 4, which corresponds to a total diet consisting of both C_4_ diet and C_4_ protein. An additional ~15% plot within the area where Clusters 1 and 4 overlap, suggesting these individuals most likely consumed some C_4_ plants, although it is not clear how much. A Queen's Chapel of the Savoy (QCS 1123) individual plots closer to Cluster 2 (30%C_3_:70%C_4_ diet; >50%C_4_ protein), suggesting that his diet was based predominantly on C_4_ sources, and not marine foods (Figure 5). When sites are grouped by region in either London or the North, a higher proportion of London populations (73%) plot within Cluster 4 (70%C_3_:30%C_4_ diet; ≥65%C_3_ protein) than those from the North (18%), indicative of a higher C_4_ contribution to the diet in London overall.

## 4 DISCUSSION

### 4.1 Animal management

With the exception of three pigs, all animals exhibited 𝛿^13^C_coll_, 𝛿^13^C_carb_ and 𝛿^15^N values consistent with livestock raised in the British Isles grazing on C_3_ forage and fodder crops. The substantial range in cattle and sheep 𝛿^15^N values (5.4‰ and 7.2‰, respectively) likely reflects the large geographical range from which livestock were drawn to feed the burgeoning industrial towns (Scola, 1992) and the diversity in environmental conditions and land management practices in these areas, particularly the intensity of manuring (Doherty et al., 2023). Elevated 𝛿^15^N values in some pigs and domestic fowl indicate a diet with a component of animal protein, typical of omnivores consuming human refuse (Halley & Rosvold, 2014; Hammond & O'Connor, 2013).

Three pigs and one cow produced isotope values indicative of a mixed C_3_/C_4_ diet, and potential foddering on imported C_4_ crops such as maize (Figures 3 and 4b). Such values may also suggest these animals were of non‐domestic origin and highlight the increasing number of livestock imported from the Americas (Trow‐Smith, 1959).

### 4.2 Interpreting human diet using 𝛿 ^13^C_coll_ and 𝛿 ^15^N

Generally, the 𝛿^13^C and 𝛿^15^N values for all analyzed individuals in this study indicate that their diet was heavily C_3_‐based. The northern diet is broadly similar, however, a Kruskal–Wallis *H* test showed that there were statistically significant differences in 𝛿^13^C and 𝛿^15^N values between different sites and Hazel Grove, a lower‐class population (Table 5). Nevertheless, the mean 𝛿^13^C and 𝛿^15^N differences between these sites are too small (average 0.4‰ and 0.8‰ respectively) to suggest interpretable dietary variations as the cause of these statistically significant differences. Focusing on London, the wider comparison of human collagen data from both this and other published studies supports a different dietary trend for London in comparison to other areas of England at that time (Figure 3b). Humans from London sites have higher mean 𝛿^15^N values relative to other areas, suggesting London's better access to high trophic level protein than other cities in England, even at lower social levels. The human‐animal collagen offsets for London reflect that these populations consumed more higher trophic level animal protein than those in the manufacturing towns of the north, with a greater potential input of aquatic resources. Collagen 𝛿^13^C data suggest that all individuals, bar the outlier QCS 1123 (previously identified by Bleasdale et al. (2019) as having been born elsewhere), were consuming C_3_ plants.

**TABLE 5:** Kruskal–Wallis *H* test and post hoc *p* value results for the northern populations after the Holm's Sequential Bonferroni adjustment.

Isotopes	Kruskal–Wallis *H* test	Site	CSM	HGM	FEW	SCH	SGC	VGL	ROM
δ^13^C_coll_ (‰)	*X* ^2^ (6) = 19.52; *p* < 0.05	CSM	‐	0.000	0.075	0.572	0.707	0.214	0.800
		HGM	0.000	‐	0.410	0.001	0.003	0.575	0.002
		FEW	0.075	0.410	‐	0.157	0.089	0.978	0.134
		SCH	0.572	0.001	0.157	‐	0.479	0.312	0.821
		SGC	0.707	0.003	0.089	0.479	‐	0.188	0.610
		VGL	0.214	0.575	0.978	0.312	0.188	‐	0.275
		ROM	0.800	0.002	0.134	0.821	0.610	0.275	‐
δ^15^N (‰)	*X* ^2^ (6) = 23.3; *p* < 0.05	CSM	‐	0.000	0.311	0.813	0.649	0.986	0.108
		HGM	0.000	‐	0.065	0.000	0.001	0.046	0.012
		FEW	0.311	0.065	‐	0.392	0.252	0.541	0.978
		SCH	0.813	0.000	0.392	‐	0.560	0.914	0.189
		SGC	0.649	0.001	0.252	0.560	‐	0.816	0.139
		VGL	0.986	0.046	0.541	0.914	0.816	‐	0.482
		ROM	0.108	0.012	0.978	0.189	0.139	0.482	‐

*Note*: Statistically significant differences in all red *p* values.

Abbreviations: CSM, Cross Street Unitarian Chapel, Manchester; HGM, Hazel Grove, Manchester; FEW, Fewston; SCH, Square Chapel, Halifax; SGC, St George's Crypt, Leeds; VGL, Victoria Gate Leeds; ROM, Rotherham.

### 4.3 Interpreting diet using 𝛿 ^13^C_coll_ , 𝛿 ^15^N_,_ and 𝛿 ^13^C_carb_

The application of carbonate isotopic modeling techniques is becoming a significant component in palaeodietary interpretations as they provide more detailed insights into past diets than the traditional methods using bone collagen alone for example, (Olsen et al., 2016; Reitsema et al., 2017; Somerville et al., 2013). Our results indicate that only around 18% of the northern populations exhibited a diet consisting of some C_4_ foods, either through C_4_‐foddered animals and/or through direct consumption of cane sugar or maize. The northern individuals in this category were from lower‐class Rotherham, middle/upper‐class Cross Street (Manchester), middle/upper‐class St George's Crypt (Leeds) and mixed‐status Square Chapel (Halifax) populations. All populations except Rotherham were middle or upper social class, therefore it is unlikely that they directly consumed maize, as it was regarded as animal fodder by those classes (Hill, 1977; Holland, 1919). Individuals of a range of socioeconomic statuses seem to be consuming sugar, perhaps most surprisingly at Rotherham, which was poor and predated the 1874 Abolition of Sugar Tax when cane sugar became available to all social classes (Mintz, 1986; Walvin, 2017). High consumption of sugar for Rotherham individuals is most likely a result of the cane sugar price drop between 1800 and 1850 that was a result of equalization of cane sugar duties in Britain, which resulted in the national sugar consumption rising from 300 million in 1800 to a billion pounds (lbs) in 1852 (Mintz, 1986; Walvin, 2017). Once the sugar duties were equalized, cane sugar consumption also increased for the poorer classes. Furthermore, individuals consumed sugar as a cheap form of energy or in tea (Mintz, 1986), making it possible for the lower‐class Rotherham individuals' C_4_ diet to have derived from cane sugar as well as C_4_ animal protein.

In London, by contrast, a larger proportion of the population (73%) had some C_4_ input into their diet, including individuals of both high and low socioeconomic status. Again, there are similar sources to those in the North that most likely contributed to this C_4_ signature. Unlike the northern populations, some individuals from the London sites plot above the Cluster 4 zone and these high Function 2 values (hence high 𝛿^15^N values) suggest consumption of a greater proportion of C_4_ animal protein than the rest of the populations studied. In addition, since these individuals plot between Cluster 1 (100% C_3_ diet; 100% C_3_ protein) and Cluster 3 (50% C_3_:50%C_4_ diet; Marine protein), it is possible that they also could have been consuming marine protein. Consumption of marine protein is likely to be a factor in the high Function 2 values given the documentary evidence for the consumption of cheap marine foods in London (Picard, 2006; Tames, 2003). Additionally, higher 𝛿^15^N values observed in London populations are similar to those of high‐status individuals from Chichester, which have also been attributed to marine protein (see Figure 3a). Low marine intake cannot be discounted from our interpretations. The isotopic data here is congruent with the historical evidence that indicates that Londoners consumed more animal protein than the rest of the country during this period (Metcalfe, 2015; Picard, 2006; Spencer, 2000; Trow‐Smith, 1959). It has already been established that Londoners were receiving livestock from a wide range of areas, therefore it is feasible that they also consumed more C_4_ animal protein.

Additionally, London was the commercial, financial, and trading center behind Britain's sugar colonies (Walvin, 2017). From the 1650s, there is evidence from London that indicates that cane sugar was being used as an additive to hot beverages such as tea, which had become popular by 1704 because of the transformation in British tea drinking enabled by the increased importation of tea to the city from China (Walvin, 2017). By 1800, the poor in the city had also become attached to cane sugar as a sweetener in tea, the latter of which had become more cheaply available by the reduction of tea duties. Notably, London employers began providing their domestic servants sugar and tea as a replacement for the ration of beer, traditionally granted to servants as part of their food allowance (Graham, 2008, p. 72). Therefore, it is possible that the poor individuals from Royal London hospital (1825–1841) displaying a C_4_ signature in their diet were consuming a significant amount of cane sugar and C_4_ animal protein, especially since these individuals died before the Great Irish Famine and therefore would not have been consuming maize.

On the other hand, the lower‐class St Brides' Lower population included lodgers and inhabitants of the Bridewell workhouse. During the Great Irish Famine, many Irish people migrated to Britain where they stayed and worked in workhouses that provided relief maize in the form of soup or porridge (Dudley‐Edwards & Williams, 1956; Ó'Gráda, 1989). The consumption of a short‐term maize relief diet over the duration of the Famine in workhouses has also been suggested in other post‐medieval studies in England (Beaumont, 2013; Beaumont et al., 2013b). However, it is proposed here that some of the C_4_‐based diet in the St Brides Lower population individuals is unlikely to have been from maize. This is because the introduction of relief maize lasted for only about 2 years from its introduction in 1845 after Charles Trevelyan closed the food depots that had been selling maize and stopped its importation from America (Dudley‐Edwards & Williams, 1956; Ó'Gráda, 1989; Swift, 2002). The 2‐year consumption of maize is unlikely to have been sufficient to significantly alter these individuals' bone isotopic signal. Additionally, the St Brides Lower graveyard was closed in 1849, therefore, not many famine migrants could have been buried there. Therefore, similar to Royal London Hospital individuals, it is also possible that the C_4_‐based diet of St Brides Lower individuals was derived from cane sugar and C_4_ animal protein.

Looking at the mixed‐status Queen's Chapel of the Savoy population, the results suggest that all individuals had access to some C_4_ resources. This population was made up of hospital patients, parishioners, criminals, seamen, and military personnel. The heterogeneous social structure at this site makes it challenging to draw conclusions regarding the C_4_ source, but given the military connections of the site, it is highly likely that at least some of these individuals could have had access to C_4_ resources abroad. This is probably the case for QCS 1123, with a significant C_4_ diet indicating that he is likely to have originated from elsewhere, potentially America (Bleasdale et al., 2019). It is also possible that the C_4_ resources at this site could have been rum, or beer made with molasses, products of sugarcane. Historical evidence indicates that sailors and military personnel were provided with food substitutions such as rum and spruce beer made from molasses as there were limited supplies of food during this period (Knight & Wilcox, 2010).

Finally, the wealthy St Barnabas population is particularly notable for having the highest number and a high proportion (78%) of individuals (*n* = 18) who consumed C_4_ resources. As suggested for other middle/upper social class populations in the country, these individuals are most likely to have obtained their C_4_ signature from both the consumption of cane sugar and C_4_‐fed animal protein.

## 5 CONCLUSIONS

This study is the first to obtain human and animal 𝛿^13^C_carb_ data in post‐medieval England. The multi‐proxy isotopic approach to a large sample of animal and human populations provided a detailed insight into animal management and the diet of 17th–19th c. human populations. Previous dietary interpretations based on analysis of carbon and nitrogen stable isotopes from bone collagen have largely been adequate in documenting diets dominated by C_3_ plants and animal products. Where C_4_ diets were significant, these previous studies have only been able to make suppositions that C_4_ resources were available, but it was not possible to clearly demonstrate the presence of C_4_ source(s) in the diet by analyzing collagen alone. With the addition of bone carbonate analysis and statistical modeling, consumption of C_4_ resources has been revealed with greater clarity. Issues of equifinality between consumption of marine and C_4_‐fed animal protein sources remain however, and future research should be directed toward single compound approaches to more identify between consumption of terrestrial C_4_ and marine diets (Corr et al., 2005).

Our results demonstrate that within these post‐medieval English populations there were significant variations in dietary patterns affecting the consumption of animal protein and C_3_ and C_4_ at both regional and site‐specific scales. Individuals in London had greater access to animal protein and foods enriched in ^13^C compared to those in the manufacturing northern towns. Intrapopulation variability was also evident, with some individuals consuming more C_4_ resources than the remainder of their respective populations. Differences in diet between sites present striking evidence of the role played by social status in the type and quantity of food that was available to different individuals during this period. Overall, individuals from the middle and upper classes had greater access to animal protein and C_4_ resources than their lower social class counterparts, although the availability of sugar to low‐status populations is hypothesized here. This multi‐isotope approach enhances our knowledge of variation and the role of C_4_ crops in the post‐medieval diet in England. Furthermore, this research presents a large new multi‐isotope dataset deriving from multiple post‐medieval sites representing major regional centers in England to consider diets in terms of geographical location and socioeconomic status. Altogether, our work further highlights the role of a multi‐isotope approach in future research exploring the addition of maize and sugar cane in Europe during this dynamic period of food globalization.

## AUTHOR CONTRIBUTIONS

**Blessing Chidimuro:** Conceptualization (lead); data curation (lead); formal analysis (lead); funding acquisition (lead); investigation (lead); methodology (lead); project administration (lead); resources (lead); validation (lead); visualization (lead); writing – original draft (lead); writing – review and editing (lead). **Sean Doherty:** Data curation (supporting); formal analysis (supporting); resources (supporting); writing – review and editing (supporting). **Jonathan Finch:** Supervision (supporting); writing – review and editing (supporting). **Paola Ponce:** Formal analysis (supporting); methodology (supporting); resources (supporting); writing – review and editing (supporting). **Jack Eggington:** Data curation (supporting); writing – review and editing (supporting). **Sarah Delaney:** Data curation (supporting); formal analysis (supporting); writing – review and editing (supporting). **Camilla Speller:** Resources (supporting); supervision (supporting); writing – review and editing (supporting). **Matthew Collins:** Conceptualization (supporting); formal analysis (supporting); funding acquisition (supporting); supervision (supporting); writing – review and editing (supporting). **Malin Holst:** Data curation (supporting); formal analysis (supporting); resources (supporting); supervision (supporting); writing – review and editing (supporting). **Michelle Alexander:** Conceptualization (equal); data curation (supporting); formal analysis (supporting); funding acquisition (equal); investigation (supporting); methodology (supporting); resources (equal); supervision (lead); validation (equal); visualization (equal); writing – review and editing (supporting).

## Supporting information


**DATA S1.** Supporting Information.

**DATA S2.** Supporting Information.

**DATA S3.** Supporting Information.

**DATA S4.** Supporting Information.

## Data Availability

The data that support the findings of this study are available in the Supporting Information material of this article.
