# Supplementary material for: North and South: Exploring isotopic analysis of bone carbonates and collagen to understand post‐medieval diets in London and northern England
Source: Am J Biol Anthropol. 2023 Jul 22;182(1):126–42. doi: 10.1002/ajpa.24818 (PMC10952890; doi:10.1002/ajpa.24818)
Supplement: Supplementary file 1 — DATA S1. Supporting Information. [file AJPA-182-126-s004.docx]

**Supporting Information 1**

**1 | Sites in Greater Manchester**

Human remains from this region were sampled from two sites: Cross Street Unitarian Chapel, Manchester and Hazel Grove, Greater Manchester. All collagen, FTIR, and carbonate data from this region were generated as part of the laboratory work carried out for this study, as none existed beforehand.

**1.1 | Cross Street Unitarian Chapel**

Archaeological excavations at Cross Street Unitarian Chapel, Manchester, were commissioned by Transport for Greater Manchester (project code TFGM3/2162) and conducted by CFA Archaeology Ltd. between September 2014 and November 2015 in advance of the construction of the Second City Crossing tramway along Cross Street in the northern half of Manchester city centre (CFA Archaeology Ltd. 2017a, 5–6). The Unitarian Chapel was originally erected in 1694 by Reverend Henry Newcome to accommodate his growing congregation and the cemetery was in use for just over 130 years, with the earliest grave (no surviving skeleton) dating to 1720 and the latest to 1852. Like many other non-conformist groups, the chapel was initially attended by the poorer classes, however, with time the chapel recruited more prosperous members from the manufacturing classes or those who were artisans or merchants [(Baker, 1884)](https://paperpile.com/c/6Kd6Sj/lL0TV). People interred at the Cross Street burial ground came from wealthy streets in Manchester such as Ardwick, Mosley Street, Brazennose Street, Holme Street and Princess Street [(CFA Archaeology Ltd., 2017b)](https://paperpile.com/c/6Kd6Sj/VnO7). Some of the members of the congregation who were buried at the chapel in the late 18^th^ century include wealthy cotton merchants, such as Thomas and Richard Potter [(Baker, 1884, p. 95)](https://paperpile.com/c/6Kd6Sj/lL0TV/?locator=95). The first Mayor of Manchester, Sir John Potter, a ‘very wealthy man’, according to the description given by Messinger [(1985, p. 59)](https://paperpile.com/c/6Kd6Sj/0iIFo/?locator=59&noauthor=1), also worshipped in this chapel. Sir John Potter himself was not exhumed during the redevelopment of Cross Street but some individuals excavated who are part of this study had grave markers denoting the burials of members of the Potter family, for instance, Skeleton 2.31 (Elizabeth Potter). On the whole, the population is considered to be modest-living middle-class citizens. A total of 241 individual skeletons and 17,679 fragments of disarticulated bone were recovered. Among the individuals recovered at this site, 172 could be identified based on legible nameplates, coffins with studwork text, or legible gravestones, as well as documentary sources, nine of whom were analysed in this study [(CFA Archaeology Ltd., 2017b, pp. 5–6)](https://paperpile.com/c/6Kd6Sj/VnO7/?locator=5-6).

Osteological analysis carried out by York Osteoarchaeology Ltd. on all 241 skeletons as well as on disarticulated bones recovered at this site revealed that the proportion of non-adults recovered (28%) was lower than expected from historical documents for this period. The London Bills of Mortality suggest that around 50% of the population died before the age of twenty years between the early 18^th^ and mid-19^th^ centuries [(Roberts & Cox, 2003, p. 304)](https://paperpile.com/c/6Kd6Sj/aZW0j/?locator=304). Among the sexed adults, there was a slightly higher proportion of males (51.1%) than females (48.9%) [(Keefe & Holst, 2017)](https://paperpile.com/c/6Kd6Sj/oz5qm). In terms of osteological indicators of diet and nutrition, dental health at this site was generally poor; females showed high levels of caries, with 20.7% of their teeth affected compared to 18.5% of teeth from males. Antemortem tooth loss was observed for both males and females. Just over three-quarters of the adults with at least one observable tooth position (94/122, 77.0%) had experienced antemortem tooth loss and 27.5% of their teeth had been lost antemortem. This is considered consistent with a diet of refined sugars and processed carbohydrates. Conditions associated with lack of specific nutrients such as scurvy (lack of vitamin C which can be found in fresh fruit, vegetables, and marine fish) and rickets (lack of vitamin D, which is produced by the body during exposure to sunlight as well as other sources like oily fish, eggs, and milk) were observed in non-adults. The non-adult prevalence rates for scurvy and rickets in this population were 5.9% and 4.4% respectively, relatively low when compared to contemporary urban populations [(Keefe & Holst, 2017)](https://paperpile.com/c/6Kd6Sj/oz5qm). Children with these conditions also had evidence of higher rates of childhood stress enamel defects than adults [(Keefe & Holst, 2017)](https://paperpile.com/c/6Kd6Sj/oz5qm).

**1.2 | Chapel Street Hazel Grove**

In 2016, CFA Archaeology excavated the site of a former Wesleyan Chapel on Chapel Street, Hazel Grove, Greater Manchester. Hazel Grove is presently a suburb located within the Metropolitan Borough of Stockport, approximately 9 miles from Manchester city centre (Figure 1). The Wesleyan Chapel dates to around 1785 and a Sunday school was in place between c.1823-1912 and the cemetery was in use between 1794 and 1910. Most of the population living in Hazel Grove were those of the poor classes [(Errington, 2001; Newman & Holst, 2016)](https://paperpile.com/c/6Kd6Sj/8MdEx+EjMvM). Osteological analysis was carried out on 39 skeletons recovered from the southwest and northwest area behind the chapel by York Osteoarchaeology Ltd in 2016 [(Newman & Holst, 2016)](https://paperpile.com/c/6Kd6Sj/EjMvM). The nameplates recovered during the excavations (Supporting Table S1) reveal that the excavated individuals were representative of the 18^th^ and early 19^th^ century members of the congregation [(Newman & Holst, 2016)](https://paperpile.com/c/6Kd6Sj/EjMvM).

Only a small proportion of the adult burial population could be sexed, indicating the presence of six males and seven females. Half of the population was made up of non-adults, with a high proportion of infants (1 to 11 months, 52.6%) and young juveniles (1 to 6 years, 31.6%) which corresponds with historical documentation that states that a large proportion of deaths occurred within the first five years of life during this time period (Cherryson, Crossland & Tarlow, 2012).

Osteological analysis also revealed poor dental health at this site [(Newman & Holst, 2016)](https://paperpile.com/c/6Kd6Sj/EjMvM)**.** Females showed higher levels of caries than males, but there was disproportionality between sexes with 49.1% of their teeth affected compared to 7.2% of teeth from males. This was likely skewed by two female individuals that had a significant number of carious lesions [(Newman & Holst, 2016)](https://paperpile.com/c/6Kd6Sj/EjMvM). Ante-mortem tooth loss was observed in both sexes. Just under three-quarters of the adults from Hazel Grove (11/15, 73.3%) had experienced antemortem tooth loss and 22.64% of their teeth had been lost antemortem. Similar to Cross Street, poor dental health was consistent with a diet of refined sugars and processed carbohydrates. Palaeopathological analysis of the Hazel Grove population suggested that this group had suffered from periods of ill health. Six infants and young juveniles suffered from scurvy and rickets, with a prevalence rate of 15.8% (3/19) for both [(Newman & Holst, 2016)](https://paperpile.com/c/6Kd6Sj/EjMvM). Enamel defects, *cribra orbitalia*, and residual rickets, all evidence of childhood stress, were also identified among adults.

Within the Hazel Grove population, four individuals tentatively exhibited cranial morphological traits as described by Byers [(2010, pp. 154–165)](https://paperpile.com/c/6Kd6Sj/kTXuS/?locator=154-165&noauthor=1) that could be suggestive of African/mixed ancestry (SK 1, SK 10, SK 14 and SK 21, [(Newman & Holst, 2016, pp. 24–25)](https://paperpile.com/c/6Kd6Sj/EjMvM/?locator=24-25). DNA test results confirmed that two of the individuals (SK 1 and SK 10) were likely of mixed ancestry [(Banken, 2018)](https://paperpile.com/c/6Kd6Sj/UCXTx).

**2 | Sites in Yorkshire**

Human remains from this region were sampled from five sites: Fewston, Harrogate; Square Chapel, Halifax; St George’s Crypt, Leeds; Victoria Gate, Leeds; and Rotherham Minster, Rotherham. Collagen data for Fewston were obtained from Poppy Yapp and Chloe Brown, for Square Chapel from Sarah Delaney and Orsolya Czére, and for Victoria Gate from Michelle Alexander’s unpublished material. However, the remaining collagen data for five of the Square Chapel individuals, all Rotherham Minster and St George’s Crypt individuals as well as all FTIR and carbonate data were generated as part of the laboratory work carried out for this study.

**2.1 | Fewston, Harrogate**

Fewston is a small village located in the Washburn Valley, 9 miles West of Harrogate, 14 miles East of Skipton, and 9 miles North of Otley. The 18^th^ century saw many local families in this village earn the right to acquire and maintain land for stock rearing and better cultivation. Many of the named individuals in this assemblage were farmers and textile workers of varying social statuses [(Caffell & Holst, 2017)](https://paperpile.com/c/6Kd6Sj/ncKBJ). Seven of the human individuals sampled for this study were part of 154skeletons recovered during archaeological excavations in 2009 and 2010, of the churchyard of St Michael and St Lawrence, Fewston, North Yorkshire [(Caffell & Holst, 2017)](https://paperpile.com/c/6Kd6Sj/ncKBJ). The excavations were carried out by John Buglass Archaeological Services (JBAS) prior to the construction of a Heritage Centre at Fewston Church. Of the 154 individuals excavated, twenty-six were confidently identified and named; 11 from their association with grave monuments and 15 from legible and partially legible coffin plates [(Buglass, 2009; Caffell & Holst, 2017)](https://paperpile.com/c/6Kd6Sj/3jJWR+ncKBJ). A notable addition to the information available for this population are the diaries of John Dickinson, a late 19^th^ century resident of the adjacent village of Greater Timble. They were written between 1878 and 1912, furthermore, an edited version of the diaries is also available [(Harker, 1988)](https://paperpile.com/c/6Kd6Sj/lVpYR). These diaries refer to some of the identified individuals within the skeletal assemblage, providing unique personal insights into everyday life in the Washburn Valley [(Caffell & Holst, 2017)](https://paperpile.com/c/6Kd6Sj/ncKBJ). Information from coffin plates and headstones revealed that burials date predominantly to the latter half of the 19^th^ century [(Caffell & Holst, 2017)](https://paperpile.com/c/6Kd6Sj/ncKBJ). The seven individuals sampled for this study date to the 19^th^ century and include three named individuals identified by coffin plates: George Lister, date of death 19th July 1882 (Skeleton 130); Christiana Patterson nee Hardwick (Skeleton 156); and Elizabeth Dermaine, nee Dickinson, date of death 6th April 1888 (Skeleton 238).

**2.2 | Square Chapel Halifax**

The town of Halifax is situated approximately 17 miles southwest of Leeds and 28 miles northeast of Manchester. The town began to transform in the late 18^th^ century due to the development of woollen and textile industries in the area. There are prints available from the 18^th^ century that reveal that Halifax was largely rural with many small, enclosed fields (Engl, Potten & Pollington, 2013; Hargreaves, 1999, p. 112). However, by 1790, the town was described as a place filled with smoke emitted from factories, with about 46 cotton mills [(Hargreaves, 1999, p. 70)](https://paperpile.com/c/6Kd6Sj/JYWL8/?locator=70). During this period, there were several people migrating into the township from outside Halifax including many, again, from Ireland [(Hargreaves, 1999)](https://paperpile.com/c/6Kd6Sj/JYWL8). The population in Halifax rose from 5,000 to 25,159 between 1743 and 1851, turning the town from a rural settlement to an urban centre [(Hargreaves, 2003, p. 73)](https://paperpile.com/c/6Kd6Sj/aiwB1/?locator=73). However, it must be noted that the town did not develop as much as the nearby Manchester and Leeds urban cities because of its geography that made transportation of goods difficult, preventing the large-scale shipments of the coal necessary to power the steam engines in mills, restricting the manufacturing industries from becoming wholly dominant in the town [(Hargreaves, 2003)](https://paperpile.com/c/6Kd6Sj/aiwB1). During the 18^th^ and 19^th^ centuries, the town appears to have consisted of a population from a variety of economic backgrounds, including those living on or below the poverty line, the working classes, artisans, and the newly established middle classes [(Hargreaves, 2001)](https://paperpile.com/c/6Kd6Sj/YK2Qj). Studies have indicated that most of the poorer people in Halifax worked in the textile industry, working for most of the middle and upper classes who were manufacturers and merchants [(Iwama, 2003)](https://paperpile.com/c/6Kd6Sj/BcCIk).

The Square Chapel site is located on the eastern side of the modern Halifax town. The site, previously known as Square Independent Chapel, was a Congregationalist non-conformist chapel. It was excavated in 2014 by the Archaeological Services WYAS and osteological analysis was carried out on the 203 skeletons excavated [(Keefe & Holst, 2015)](https://paperpile.com/c/6Kd6Sj/hFDaC). The burial register held at the National Archive in Kew shows records for Square Chapel date burials occurring between 1771 to 1837. Although Square Chapel ceased being a chapel in 1857 gravestone inscriptions indicate that burials continued until 1870. Six individuals were identified by coffin plates and only one of them (William Fletcher, Skeleton 35) was included in this study. These six coffin plates indicate that the individuals died in the 19^th^ century [(Keefe & Holst, 2015)](https://paperpile.com/c/6Kd6Sj/hFDaC). Osteological analysis revealed that the burial population comprised a slightly higher proportion of females than males, which, as previously mentioned, is common in non-conformist burial grounds [(Hargreaves, 1999, pp. 97–98)](https://paperpile.com/c/6Kd6Sj/JYWL8/?locator=97-98). About 65% of the skeletal assemblage were adults with 35% being non-adults, with a high proportion of infants (1-12 months) and young juveniles (1-6 years). It appears that there are likely variations in the socioeconomic status of those buried at Square Chapel, but it is impossible to differentiate this by burials themselves. The mixed socioeconomic status of these individuals may be due to the individuals being part of a non-conformist congregation as these appealed to individuals from a variety of backgrounds and professions, such as artisans, shopkeepers, and manufacturers, and did not typically attract only one class [(Keefe & Holst, 2015)](https://paperpile.com/c/6Kd6Sj/hFDaC). In addition, historical sources imply that living conditions were mixed, with the poorest of society enduring the worst conditions close to the factories, while those that could afford the luxury of living further away from the industrial pollution enjoyed better conditions [(Hargreaves, 2003; Webster, 1998)](https://paperpile.com/c/6Kd6Sj/Ig3wl+aiwB1). Individuals sampled from this site are therefore intended to characterise a mixed-status diet in England.

**2.3 | St George’s Crypt, Leeds**

St George’s Crypt is located in the city of Leeds, West Yorkshire in Northern England, approximately 170 miles north of central London. As was the case for Manchester City, inventions of new processes in linen and wool manufacturers in Leeds developed rapidly in the late 18^th^ century resulting in population growth from 15,000 to 309,119 between 1760 and 1881 in the city [(Caffell & Holst, 2009; Jacob, 2007)](https://paperpile.com/c/6Kd6Sj/91Sh3+YnBqr).

In February 2009, St George’s Crypt in Leeds was excavated prior to the construction of an extension to the south of St George’s church in the city. This church, built between 1836 and 1839, had no graveyard. Instead, it had a crypt and burial vaults beneath the podium which were in use between 1840 and 1911. Osteological analysis was carried out on eight articulated skeletons and an assemblage of disarticulated human bone and the analysis identified the individuals as 22 adults and 8 non-adults (one neonate, two infants, one young child, and four adolescents). Both sexes were represented. Since the individuals recovered at this site were found in vaults, they were probably buried within small family groups because it has been suggested that in the 19^th^ century wealthy families would reserve particular vaults for the future burial of their members (Mays, Ives, & Brickley, 2009). Unfortunately, there was not enough information on the identities of the individuals buried within each vault to confirm if this was the case at this site. Although vault burials are associated with wealthier classes, the social status of the individuals in this study is unknown as there was no churchyard to accommodate the poor. The males were taller (175.8cm) than average for the post-medieval period, while the females were slightly below average height (158.0cm). The above-average height of the males, however, could suggest that these individuals were not accustomed to episodes of poor nutrition and disease during their childhood, indicating that they may have been wealthy. Furthermore, data from the grave markers in the crypt suggest that some of the individuals may have been surgeons [(Caffell & Holst, 2009)](https://paperpile.com/c/6Kd6Sj/YnBqr). Therefore, these individuals will be considered to have been from the middle/upper class in this study.

**2.4 | Victoria Gate Leeds**

Victoria Gate is located in the centre of Leeds City. The human remains from this site were associated with a Methodist chapel, having been excavated from the vicinity of Ebenezer Chapel, a Baptist chapel sold to the Methodists in 1797 [(Beckwith, 1938)](https://paperpile.com/c/6Kd6Sj/yRcyE). The remains were excavated in 2013 and 2014 and osteological analysis was carried out by York Osteoarchaeology Ltd in 2014. They date to the late eighteenth or nineteenth centuries and were believed to represent some of the poorest in Leeds. Overall, the assemblage represented a minimum of 28 individuals; twelve adults and sixteen non-adults. Nine of the twelve adults at the site had developed metabolic diseases which included scurvy, rickets, and possibly anaemia [(Caffell & Holst, 2014)](https://paperpile.com/c/6Kd6Sj/UJFD7).

**2.5 | Rotherham Minster**

Rotherham is located in South Yorkshire, England, approximately 32.2 miles to the south of Leeds and 43 miles to the east of Manchester. Archaeological excavations of Rotherham Minster, previously known as All Saints Church, were carried out between early December 2009 and late February 2010 by Archaeological Services WYAS. Osteological analysis was performed on 60 skeletons by York Osteoarchaeology Ltd [(Keefe & Holst, 2011)](https://paperpile.com/c/6Kd6Sj/zX4MN). Skeletal evidence suggests that this population was probably of the lower social class who worked in coal mines, foundries, and glassworks, as well as other industries in the town. Historical records indicate that the Rotherham Minster graveyard was closed due to the expanding population as a result of the growth in the iron and steel industry between 1800 and 1854. These sources also suggest that in Rotherham, the poor were living in cramped areas with poor facilities, air and water pollution, poor hygiene, poor sewage disposal, limited access to clean water, and inadequate nutrition, and medical care [(Jones, 1995, 1996; Munford, 2003; Munford & Munford, 2000)](https://paperpile.com/c/6Kd6Sj/pwjbC+T95ld+zc8Vc+b0Fq8). These poor living conditions resulted in two outbreaks of cholera in 1832 and 1849 [(Hague, 2018; Underwood, 1935)](https://paperpile.com/c/6Kd6Sj/zqVk9+JTkTX). Osteological analysis revealed that the quantity and severity of disease manifestations compared with those from contemporary populations were unusual thereby supporting the extent of poor living conditions in Rotherham [(Keefe & Holst, 2011)](https://paperpile.com/c/6Kd6Sj/zX4MN). Nonadults made up 40% of the assemblage whereas 60% was made up of adults, a group for which there was near parity of sexes. The analysis also revealed that children often did not survive beyond the age of three years due to nutritional deficiencies. It has been suggested that the sizable number of adolescents (13-17 years) among the nonadults most likely represents the immigration of adolescents into towns for work, where they would have been exposed to infectious diseases, they had no immunity against [(Keefe & Holst, 2011)](https://paperpile.com/c/6Kd6Sj/zX4MN).

**3 | Sites in London**

Human remains from this region were sampled from four sites, Queen’s Chapel of the Savoy; St Barnabas/St Mary Abbots, Chelsea; Royal London Hospital; and St Bride’s Lower sites. Collagen data for Queen’s Chapel of the Savoy and St Barnabas/St Mary Abbots, Chelsea were obtained from Bleasdale et al. [(2019)](https://paperpile.com/c/6Kd6Sj/HckiH/?noauthor=1). However, the remaining collagen data for Royal London Hospital and St Bride’s Lower sites and all FTIR and carbonate data in this study were generated as part of the laboratory work for this study

**3.1 | Queen’s Chapel of the Savoy**

The Queen’s Chapel of the Savoy cemetery site is located in the City of Westminster London. King Henry VII commissioned the Savoy Hospital to be built in 1502 on the former site of the Savoy Palace once owned by Henry, Duke of Lancaster, which had been destroyed in the 1381 Peasants Revolt [(Somerville, 1960)](https://paperpile.com/c/6Kd6Sj/ymadf). The Savoy hospital, which included the Queen’s Chapel and churchyard serving as a burial ground, was first used in 1523 for the poor [(Thornbury, 1878)](https://paperpile.com/c/6Kd6Sj/2KRMl). Following this, the hospital was used as a military infirmary during the British Civil War (1642 - 1651), a naval hospital for injured seamen during the Dutch Wars (1652-1667) and finally in 1679, part of the hospital was converted to a prison, housing offenders, deserters and prisoners of war [(Firth, 1902; Keevil, 1957; Strype, 1720)](https://paperpile.com/c/6Kd6Sj/HgNkV+oAzx4+2xhip). The hospital was dissolved in 1702 after its possession by the Treasury; by 1816, all buildings except the chapel had been demolished [(Somerville, 1960)](https://paperpile.com/c/6Kd6Sj/ymadf). The burials in the churchyard began in 1552 and throughout the hospital’s many changes, hospital patients, parishioners, criminals, seamen, and military personnel were buried there until 1854 when it was closed after the Burial Act of 1853 [(Sibun & Ponce, 2018; Somerville, 1960)](https://paperpile.com/c/6Kd6Sj/ymadf+mzmny). The graves could not be phased; however, Sibun and Ponce (2018) suggested that the surviving graves are most likely those from later years. Archaeological excavations by Archaeology South-East, UCL were performed in 2011 in response to the redevelopment of the chapel. In total 609 burials were recovered and for those whose sex could be determined, 76% were male and 24% were female [(Sibun & Ponce, 2018)](https://paperpile.com/c/6Kd6Sj/mzmny). It has been suggested that the difference in the sex distribution at the site was due to the considerable use of the cemetery for military personnel burials [(Sibun & Ponce, 2018)](https://paperpile.com/c/6Kd6Sj/mzmny). Additional information regarding the site is found in Bleasdale et al. [(2019)](https://paperpile.com/c/6Kd6Sj/HckiH/?noauthor=1).

**3.2 | St Barnabas/St Mary Abbots Chelsea**

St Barnabas/St Mary Abbots individuals were buried between 1831 and 1853 and had a collective lifespan of 1760 to 1853 [(Goldsmith, 2016)](https://paperpile.com/c/6Kd6Sj/hUZ7y). St Barnabas Church site is located on Addison Road, in the borough of Chelsea and Kensington in London which to this day is a very affluent area. There is very little information known about this church. The church, consecrated in June 1829, was expanded in 1860 and remodelled in 1909 to allow for more seating. The church continued to be remodelled throughout the 19^th^ century thereby suggesting the congregants were rich enough to afford the additions [(Sheppard, 1973a, 1973b)](https://paperpile.com/c/6Kd6Sj/Vy4vC+stSew). It is therefore assumed that the individuals recovered in 1991 from a crypt at the site in response to construction work were wealthy members of the society [(Bleasdale et al., 2019; Goldsmith, 2016)](https://paperpile.com/c/6Kd6Sj/hUZ7y+HckiH). In addition, it should be highlighted that although the individuals analysed here cannot be identified directly, the burial ground includes known named wealthy individuals, further supporting the understanding that the 23 individuals analysed for carbonate isotopes in this study were also wealthy. Additional information regarding the site is found in Bleasdale et al. [(2019)](https://paperpile.com/c/6Kd6Sj/HckiH/?noauthor=1).

**3.3 | Royal London Hospital**

The Royal London Hospital is located on Whitechapel Road, about a mile to the east of London city centre. The Hospital, constructed in 1752 by Boulton Mainwaring and opened in 1757, was set up to be run as a charitable institution [(Clark-Kennedy, 1962)](https://paperpile.com/c/6Kd6Sj/F74Ep). Like all hospitals operating during the 18^th^ and 19^th^ centuries in England, the Royal London Hospital had to make burial provisions for patients who had passed away at the hospital but had no friends or relatives to collect their bodies. Some of the families and friends did not collect their relatives’ remains due to the high costs of providing decent burials and since the London Hospital was one of the few hospitals that did not charge a burial fee, many of the hospital patients and their families opted for them to be buried at the hospital [(Fowler & Powers, 2012; Howard, 1791)](https://paperpile.com/c/6Kd6Sj/3JxPa+4ByO6).

Archaeological excavations undertaken by The Museum of London Archaeology (MOLA) in 2006, revealed 636 articulated contexts and 175 contexts of disarticulated bones of unclaimed patients buried between c.1825 and 1841 [(Fowler & Powers, 2012)](https://paperpile.com/c/6Kd6Sj/4ByO6). The presence of decayed coffins, multiple burials within graves, and other nonstandard burials containing multiple body parts made it difficult to make an exact count of the number of individuals buried there. Furthermore, the absence of a burial register for the Hospital made it difficult to determine if the number of excavated remains was proportionate to the number of the buried population [(Fowler & Powers, 2012)](https://paperpile.com/c/6Kd6Sj/4ByO6). It has been suggested that some of the disarticulated bones may have been removed as a result of surgical removal or anatomical dissection for medical training. A variety of diets were specific to each patient’s circumstances and were divided into two: the common diet and the middle diet. The common diet consisted of 1 pint of milk pottage or water gruel for breakfast; 8 oz of meat every day for dinner; broth for supper six days a week; and 12 oz of bread a day. The middle diet consisted of 1 pint of panada or water gruel, 4 oz of meat every day for dinner: a pint of broth or panada for supper, and 8 oz of bread. There were no vegetables included in both diets [(Howard, 1791, p. 131)](https://paperpile.com/c/6Kd6Sj/3JxPa/?locator=131). Some cattle and sheep/goat animal remains, displaying evidence of butchering, were recovered from some of the graves suggesting that they may have been food waste for the hospital patients. However, the hospital kitchens supplied meat to both hospital staff and patients, and it has been suggested that some of the more expensive meat was most likely to have been served to staff [(Millard, 1825; Mitchell et al., 2011; Morris, 2014)](https://paperpile.com/c/6Kd6Sj/yK8zC+G20ov+ud4OZ). However, as this was a hospital patient burial ground, the eleven people sampled here are not expected to include members of the hospital staff.

**3.4 | St Brides Lower**

The site of St Brides Lower, believed to have received burials between 1770 and 1849, is located within the parish of St Brides on 75-82 Farringdon Street in the city of London. The burial cemetery is one of the three burial cemeteries of the parish linked to St Bride’s Church on Fleet Street. St Brides Lower cemetery was a consequence of overcrowding of two other burial cemeteries at the same location - the original churchyard and the church crypt [(Miles & Conheeney, 2005)](https://paperpile.com/c/6Kd6Sj/rOUeh). Excavations at St Bride’s Lower carried out between 1991 and 1992 by the Museum of London Archaeology Service (MoLAS), revealed stacked human burials in wooden coffins that had since degraded. A total of 606 individuals, 47 in vaults and 497 from the open yard were excavated from the site [(Miles & Conheeney, 2005)](https://paperpile.com/c/6Kd6Sj/rOUeh). Many parish burial grounds in England charged different rates per burial location and the types of burials were connected to different socio-economic backgrounds which resulted in two very contrasting skeletal assemblages from the parish of St Bride’s [(Miles & Conheeney, 2005)](https://paperpile.com/c/6Kd6Sj/rOUeh). While those buried in crypts were prominent members of the parish, the individuals buried at the Lower Churchyard (sampled as part of this study) were poorer members of the parish, servants, prisoners from Fleet prison, lodgers, and inhabitants of Bridewell workhouse. A total of 41 former prisoners were said to be buried at this site [(Kausmally, 2008; Miles & Conheeney, 2005)](https://paperpile.com/c/6Kd6Sj/rOUeh+XUXwX). There is, however, no information available that would indicate which group each of the fifteen individuals analysed for this study belongs to.

**4 | Animal remains**

Except for Cross Street Unitarian Chapel, Manchester, animal bones were not recovered from the sites where human remains were obtained. Consequently, animal bones examined in this study derive from contemporary sites located nearby (Table 2). Faunal collagen data for Hungate, Bedern and Walmgate were generated by Dr Sean Doherty and Ms Chloe Brown, and those from London are from Bleasdale et al. [(2019)](https://paperpile.com/c/6Kd6Sj/HckiH/?noauthor=1).

**4.1 | Cross Street, Manchester**

The faunal assemblage from Cross Street Unitarian Chapel cemetery was unstratified and highly disturbed, obfuscating dating beyond ‘medieval/post-medieval’. As a result, interpretation using these remains will be considered with great caution.

**4.2 | Norton Priory, Chester**

The 1970-87 excavations of The Augustinian Priory of St Mary, Norton Priory, revealed evidence for its occupation from AD 1134 to disillusionment in 1536. Dating the faunal assemblage proved difficult due to later landscaping and demolition of the ruins in 1928, but the animal bones sampled for this study are believed to date from the 16th to early-20th century (Brown & Howard-Davis, 2008; Wright, Corbino & Albarella, 2017).

**4.3 | Otley, West Yorkshire**

Excavations at 12 Bridge Street, Otley, in 2014 yielded butchered animal bones dated broadly to the post-medieval period [(Hunt, 2014)](https://paperpile.com/c/6Kd6Sj/Y5w7J).

**4.4 | Oulton, Leeds**

Excavations in Oulton, Leeds, revealed evidence of agricultural activity from the 18^th^ century onwards, including a structure thought to relate to a Corn Mill. A small faunal assemblage was recovered, including young and neonate lambs and calves [(Hunt, 2015)](https://paperpile.com/c/6Kd6Sj/qcq8n). The animal bones sampled were phased to the 18th-19th centuries.

**4.5 | Hungate, York**

Located adjacent to the River Foss within the historic walled centre of York, Hungate is a large complex site with a sequence of use from a 3^rd^ century Roman cemetery through to early-20^th^ century tenements and industrial activities [(York Archaeological Trust, 2015)](https://paperpile.com/c/6Kd6Sj/JZz3). Between the 15^th^ and 17^th^ centuries, the area was used for refuse pits, horticulture and the Cordwainers Hall, before more intensive use from the 18th century onwards, with dense terraced housing, gas works and warehouses. By the 19th century, the tenements of Hungate had proliferated to become *“one of the main slum districts in York”* [(Rowntree, 1901, p. 199)](https://paperpile.com/c/6Kd6Sj/Sjjy/?locator=199), before being cleared in the 1930s [(Harrison, 2015)](https://paperpile.com/c/6Kd6Sj/GZrC). The animal remains used in this study were phased to the 16^th^-20^th^ centuries [(Rainsford, 2012a)](https://paperpile.com/c/6Kd6Sj/tyNs5).

**4.6 | Bedern, York**

Located east of York Minster, the Bedern was an area of residential occupation and light industry [(Richards, 1993)](https://paperpile.com/c/6Kd6Sj/uKKY), with faunal evidence for domestic activity and animal-based crafts [(Scott, 1985)](https://paperpile.com/c/6Kd6Sj/WJBV). The animal remains used in this study were phased to the 15^th^ and 19^th^ centuries.

**4.7 | Walmgate, York**

Located in the south of the walled centre, 18^th^ and 19^th^ century Walmgate was a slum area and considered “*one of the poorest in the city*” [(Rowntree, 1901, p. 199)](https://paperpile.com/c/6Kd6Sj/Sjjy/?locator=199). Extensive deposits of sheep metapodia were recovered from clay-lined pits, believed to derive from industrial activity including nearby tanners and skin processors [(O’Connor, 1985)](https://paperpile.com/c/6Kd6Sj/fVRum). The animal remains used in this study were phased to the 18^th^-19^th^ centuries.

**4.8 | Sand-Le-Mere Caravan Park, Tunstall**

A small but diverse faunal assemblage was recovered from the excavation at Sand-Le-Mere, including goats, geese and cattle burials. The assemblage was phased broadly to the medieval/post-medieval period, although has been heavily disturbed by ploughing [(Lightfoot, 2011)](https://paperpile.com/c/6Kd6Sj/oMx6I).

**References**

Baker, S. T. (1884). *Memorials of a dissenting chapel, a sketch of the rise of nonconformity in Manchester, and of the erection of the Chapel in Cross street*. London: Simpkin, Marshall & Co.

Banken, E. (2018). *Assessing osteological indicators of ancestry through ancient genomic analyses: 10 British skeletons of putative African ancestry* (Unpublished undergraduate dissertation). University of York, York.

Beckwith, F. (1938). A Forgotten Eighteenth Century Baptist Church in Leeds. *Baptist Quarterly, 9(2),* 125-127.

Bleasdale, M., Ponce, P., Radini, A., Wilson, A. S., Doherty, S., Daley, P., Brown, C., Spindler, L., Sibun, L., Speller, C., & Alexander, M. M. (2019). Multidisciplinary investigations of the diets of two post-medieval populations from London using stable isotopes and microdebris analysis. *Archaeological and Anthropological Sciences*. *11*, 6161–6181.

doi.org/10.1007/s12520-019-00910-8

Brown, F., & Howard-Davis, C. (2008). *Norton Priory: Monastery to Museum: Excavations 1970-87*. Oxford Archaeology North.

Buglass, J. (2009). *The Church of St Michael and St Lawrence, Fewston, North Yorkshire: Interim Statement on the Archaeological Investigation*. John Buglass Archaeological Services, on Behalf of Washburn Heritage Group.

Byers, S. N. (2010). *Introduction to Forensic Anthropology (International Edition)*. Boston.

Caffell, A., & Holst, M. (2009). *Osteological Analysis St. George’s Crypt, Leeds West Yorkshire* (No. 0409). York Osteoarchaeology Ltd.

Caffell, A., & Holst, M. (2014). *Osteological Analysis Victoria Gate Leeds West Yorkshire* (No. 1614). York Osteoarchaeology Ltd.

Caffell, A., & Holst, M. (2017). *Osteological Analysis, The Church of St Michael and St Lawrence, Fewston, North Yorkshire*. (No. 1210). York Osteoarchaeology Ltd.

CFA Archaeology Ltd. (2017a). *Cross Street Chapel, Manchester: Archaeological excavations and exhumations.* (Assessment Report No. Y220/16, volume 1). CFA Archaeology Ltd.

CFA Archaeology Ltd. (2017b). *Cross Street Chapel, Manchester: Archaeological excavations and exhumations.* (Assessment Report No. Y220/16, volume 2). CFA Archaeology Ltd.

Cherryson, A., Crossland, Z., & Tarlow, S. (2012). *A fine and private place: the archaeology of death and burial in post-medieval Britain and Ireland*. Leicester: Leicester Archaeology Monograph 22.

Clark-Kennedy, A. E. (1962). *The London: a Study in the Voluntary Hospital System Vol 1, 1740-1840*. Pitman Medical Publishing.

Engl, R., Potten, S., & Pollington, M. (2013). *The Square Church, Square Road, Halifax, West Yorkshire: Archaeological Evaluation Report* (No. 51172). AOC Archaeology Group.

Errington, P. A. (2001). *The History of Hazel Grove Or should it be Bullock Smithy?* http://www.hazel-grove.com/history.html#short%20history.

Firth, C. H. (1902). *Cromwell’s Army: A History of the English Soldier During the Civil Wars, the Commonwealth and the Protectorate: Being the Ford Lectures Delivered in the University of Oxford in 1900-1*. Methuen & Company.

Fowler, L., & Powers, N. (2012). *Doctors, Dissection and Resurrection Men: Excavations in the 19th-century Burial Ground of the London Hospital, 2006*. Museum of London Archaeology Service.

Goldsmith, S. (2016). *Draft historical context report for Fewston, St Barnabas, Victoria Cross and Spitalfields* (Unpublished Historical Research Report)*.* University of York, York.

Hague, V. (2018). *Rabbit George and Me*. Troubador Publishing Ltd.

Hargreaves, J. A. (1999). *Halifax (Town & City Histories)*. Edinburgh University Press.

Hargreaves, J. A. (2001). The revival of Old Dissent: Baptists and Independents in the parish of Halifax, 1743-1851. *Transactions of the Halifax Antiquarian Society*, *9*, 79–99.

Hargreaves, J. A. (2003). *Halifax* (p. 320). Carnegie Publishing.

Harker, R. (1988). *Timble Man: Diaries of a Dalesman*. Lancashire: Hendon Publishing Co. Ltd.

Harrison, L. (2015). Creating the slum: representations of poverty in Hungate and Walmgate districts of York, 1875-1914. *Ex Historia*, *7*, 61–89.

Howard, J. (1791). An Account of the Principal Lazarettos in Europe: With Various Papers Relative to the Plague, Together with Further Observations on Some Foreign Prisons and Hospitals, and Additional Remarks on the Present State of Those in Great Britain and Ireland. In J. Johnson, D. Dilly, and T. Cadell (Eds.), Princeton University.

Hunt, R. (2014). *Land at 12 Bridge Street Otley West Yorkshire: Archaeological Evaluation* (No. Y175/14). CFA Archaeology Ltd.

Hunt, R. (2015). *Land off Fleet Lane Oulton Leeds West Yorkshire Archaeological Strip, Map and Record and Watching Brief* (No. Y191/15). CFA Archaeology Ltd.

Iwama, T. (2003). *The middle class in Halifax, 1780-1850* (Unpublished doctoral thesis). University of Leeds, Leeds.

Jacob, M. C. (2007). Mechanical Science on the Factory Floor: The Early Industrial Revolution in Leeds. *History of Science; an Annual Review of Literature, Research and Teaching*, *45*(2), 197–221.

Jones, M. (1995). *Aspects of Rotherham: Discovering Local History: Volume 1*. Wharncliffe Publishing Limited.

Jones, M. (1996). *Aspects of Rotherham: Discovering Local History: Volume 2*. Wharncliffe Publishing Limited.

Kausmally, T. (2008). Farringdon: St Brides lower churchyard. *London: Museum of London*.

Keefe, K., & Holst, M. (2011). *Osteological Analysis Rotherham Minster Rotherham South Yorkshire* (No. 0111). York Osteoarchaeology Ltd.

Keefe, K., & Holst, M. (2015). *Osteological Analysis, Square Chapel, Halifax* ( No 2115). York Osteoarchaeology Ltd.

Keefe, K., & Holst, M. (2017). *Osteological Analysis Cross Street Unitarian Chapel Manchester* (No. 0817).York Osteoarchaeology Ltd.

Keevil, J. J. (1957). The seventeenth-century English medical background. *Bulletin of the History of Medicine*, *31*(5), 408–424.

Lightfoot, M. (2011). *Sand-le-Mere Caravan Park, East Yorkshire Archaeological Evaluation* (No. Y015/11). CFA Archaeology Ltd.

Mays, S., Ives, R., & Brickley, M. (2009). The effects of socioeconomic status on endochondral and appositional bone growth, and acquisition of cortical bone in children from 19th century Birmingham, England. *American Journal of Physical Anthropology: The Official Publication of the American Association of Physical Anthropologists*, *140*(3), 410–416.

Messinger, G. S. (1985). *Manchester in the Victorian age: the half-known city*. Manchester University Press Manchester.

Miles, A., & Conheeney, J. (2005). A Post-medieval population from London: excavations in the St Bride’s Lower Churchyard 75--82 Farringdon Street, City of London, EC4. *MoLAS Studies Series. London: MoLAS*.

Millard, A. (1825). *An account of the circumstances attending the imprisonment and death of the late William Millard, formerly superintendent of the Theatre of Anatomy of St. Thomas's Hospital, Southwark.* Sutton Street.

Mitchell, P. D., Boston, C., Chamberlain, A. T., Chaplin, S., Chauhan, V., Evans, J., Fowler, L., Powers, N., Walker, D., Webb, H., & Witkin, A. (2011). The study of anatomy in England from 1700 to the early 20th century. *Journal of Anatomy*, *219*(2), 91–99.

Morris, J. (2014). Explorations in anatomy: the remains from Royal London Hospital. *Anthropozoologica*, *49*(1), 109–120.

Munford, A. P. (2003). *Iron & Steel Town: An industrial history of Rotherham*. Stroud: Sutton.

Munford, A. P., & Munford, T. (2000). *A History of Rotherham*. Sutton.

Newman, S., & Holst, M. (2016). *Hazel Grove Osteological Report Final.pdf* (No. 2116). York Osteoarchaeology Ltd.

O’Connor, T. P. (1985). *Selected Groups of Bones from Skeldergate and Walmgate (The Archaeology of York Vol 15: The Animal Bones, Fascicule 1)* (P. V. Addyman (ed.)). York Archaeological Trust.

Rainsford, C. (2012a). *Block H1 and H2: The faunal remains: 1550-1939*. York Archaeological Trust.

Richards, J. (1993). *The Bedern Foundry*. York Archaeological Trust.

Roberts, C. A., & Cox, M. (2003). *Health and disease in Britain: from prehistory to the present day*. Sutton Publishing Ltd.

Rowntree, S. (1901). *Poverty: a study of town life*. MacMillan.

Scott, S. A. (1985). *Bones from The Bedern* (AML Report 4821 85/33).

Sheppard, F. H. W. (1973a). The Holland estate: To 1874. *Survey of London*, *37*, 101–126.

Sheppard, F. H. W. (1973b). The Holland estate: since 1874*. Survey of London*, *37*, 126–150.

Sibun, L., & Ponce, P. (2018). *In life and death. Archaeological Excavations at the Queen's Chapel of the Savoy, London*. London: SpoilHeap Monograph.

Somerville, R. (1960). *The Savoy: manor, hospital, chapel*. London: Chancellor and Council of the Duchy of Lancaster.

Strype, J. (1720). *Chapter VII: liberties of the Dutchy of Lancaster, Survey of London Volume II, Book 4, Online, Sheffield*. https://www.dhi.ac.uk/strype/TransformServlet?page=book4_104

Thornbury, W. (1878). *Old and New London: VIII*. Cassell, Petter, & Galpin.

Underwood, E. A. (1935). The History of the 1832 Cholera Epidemic in Yorkshire. In *Proceedings of the Royal Society of Medicine, 28 (5)*, 603–616. doi.org/10.1177/003591573502800545

Webster, E. (1998). *William Ranger’s reports on the sanitary condition of Halifax, 1850-51*. http://www.openbibart.fr/item/display/10068/712228

Wright, E., Corbino, C., & Albarella, U. (2017). *Norton Priory, Runcorn, Cheshire: The Animal Bones*. Department of Archaeology, University of Sheffield, Sheffield.

York Archaeological Trust. (2015). Hungate species: our five-year mission. *Yorkshire Archaeology Today*, *12*, 1–18.
