# Supplementary material for: North and South: Exploring isotopic analysis of bone carbonates and collagen to understand post‐medieval diets in London and northern England
Source: Am J Biol Anthropol. 2023 Jul 22;182(1):126–42. doi: 10.1002/ajpa.24818 (PMC10952890; doi:10.1002/ajpa.24818)
Supplement: Supplementary file 2 — DATA S2. Supporting Information. [file AJPA-182-126-s001.docx]

**Supporting Information 2**

**2 | Methods**

**2.1 | Collagen analysis**

Collagen extraction of bone samples was performed using the Longin [(1971)](https://paperpile.com/c/DSwV5m/d4Zv0/?noauthor=1) method modified by Brown et al. [(1988)](https://paperpile.com/c/DSwV5m/CryYY/?noauthor=1). Bone samples, each weighing between 300 and 400 mg were demineralised by immersing them in a weak acid solution (8ml 0.6M Hydrochloric acid (HCl)). When demineralisation was complete, the samples were rinsed three times in distilled water and gelatinised in pH3 HCL at 80°C for 48 hours. Each sample was filtered using Ezee^TM^ filters to remove large unwanted insoluble particulate matter prior to ultrafiltration. The supernatants (filtered gelatinised samples) were ultrafiltered to remove contaminants of low molecular weight (<30 kDa) such as humic acid, degraded collagen fragments, and salts from the sample and isolate high molecular weight components of the gelatinised collagen (>30 kDa) using Amicon® Ultra centrifugal filters, 30 kDa [(Brock et al., 2013; Brown et al., 1988)](https://paperpile.com/c/DSwV5m/CryYY+equPu). The ultrafiltered samples were then frozen for 24 hours at -20°C before being lyophilised in a freeze dryer for 48 hours. Between 0.4 and 0.6mg of the freeze-dried retentate were weighed out in duplicate into 4x3.2mm tin capsules and combusted alongside international standards in an Elemental Analyser Isotope Ratio Mass Spectrometer (EA-IRMS): a Sercon 20-22 mass spectrometer coupled with a Sercon GSL Sample Preparation System module at BioArch, University of York.

Accuracy for collagen analysis was determined by measurements of international standard reference materials within each analytical run. These were IAEA 600 ẟ^13^C_raw_ = -27.65 ± 0.09‰, ẟ^13^C_true_ = -27.77 ± 0.043‰, ẟ^15^N_raw_ = 0.92 ± 0.21‰, ẟ^15^N_true_ = 1 ± 0.2‰; IAEA N2 ẟ^15^N_raw_ = 20.35 ± 0.13‰, ẟ^15^N_true_ = 20.3 ± 0.2‰; IA Cane, ẟ^13^C_raw_ = -11.77 ± 0.09‰; ẟ^13^C_true_ = -11.64 ± 0.03‰. The overall uncertainties on the measurements of each sample were calculated based on the method of Kragten [(1994)](https://paperpile.com/c/DSwV5m/Z2oxp/?noauthor=1) by combining uncertainties in the values of the international reference materials and those determined from repeated measurements of samples and reference materials. These are expressed as one standard deviation. The maximum uncertainty for all samples across all runs was <0.2‰ for both ẟ^13^C and ẟ^15^N. In addition, a homogenised bovine bone extracted and analysed within the same batch as the samples produced the following average values; ẟ^13^C = -23.01 ± 0.13‰; ẟ^15^N = 6.21 ± 0.44‰.  This was comparable to the overall mean value from 50 separate extracts of this bone sample, which produced values of ẟ^13^C = -22.97 ± 0.19‰ and ẟ^15^N = 6.19 ± 0.30‰.

**2.2 | FTIR analysis**

Sample preparation and analysis were executed according to the method of Kontopoulos et al. [(2018)](https://paperpile.com/c/DSwV5m/1NM9G/?noauthor=1). Prior to chemical pretreatment, the bones were cleaned using a sterile scalpel blade to remove dirt and contaminating material. Once cleaned the samples were ground using an agate mortar and pestle. The powdered samples were then sieved through Endecotts woven stainless steel mesh sieves with an aperture size of 20µm and 50µm so that only grains between 20µm and 50µm particle size would be used. Spectral analyses were performed using OPUS software (Bruker). Spectra were collected in 144 scans, in the 4000–400 cm^-1^ wavenumber range, with a spectral resolution of 4cm^-1^ and zero-filling factor of 4. Each sample was measured in triplicate. The instrument’s crystal and arm’s tip were cleaned with tissue paper soaked in propanol before each measurement. Baseline correction and spectra normalisation were carried out using the OPUS software. For each sample, the background reading was measured, and then ~ 2-3 mg of bone powder was pressed onto the diamond crystal and measured. Pressure was applied using a pressure control spot on the applicator to ensure good contact between the diamond crystal and the sample. Integration was performed on each of the spectra by defining the left and right edges for the peaks, troughs, and curves for baseline correction. Two common diagenetic parameters namely (i) the infrared splitting factor calculated as follows [IRSF = [565_ht_cm^-1^ + 605_ht_cm^-1^]/590_ht_cm^-1^] [(Weiner & Bar-Yosef, 1990)](https://paperpile.com/c/DSwV5m/me9Bh) and (ii) the carbonate/phosphate ratio calculated as follows [C/P = 1415_ht_cm^-1^/1035_ht_cm^-1^] [(Wright & Schwarcz, 1996)](https://paperpile.com/c/DSwV5m/eWuyL) as well as the presence of the calcite (CaCO_3_) band at 712 cm^-1^.peak [(Baxter et al., 1966; Hunt et al., 1950)](https://paperpile.com/c/DSwV5m/Lta4t+VpdGb) were utilised in assessing diagenesis.

**2.3 | Carbonate analysis**

Carbonate extraction of bone samples followed the standard operating procedure used at the University of York adapted from Snoeck and Pellegrini [(2015)](https://paperpile.com/c/DSwV5m/yBK5S/?noauthor=1) and Pellegrini and Snoeck [(2016)](https://paperpile.com/c/DSwV5m/DSDui/?noauthor=1). Firstly, the bones were cleaned using a sterile blade to remove dirt and contaminating material. The cleaned bones were crushed using an agate mortar and pestle that had been cleaned with distilled water. Approximately 7.5 mg of bone powder was required for each sample. The agate mortar and pestle were also cleaned with ethanol between samples to prevent cross-contamination. The bone powder for each sample as well as the bovine control samples was transferred into their respective weighed fresh 15ml centrifuge tubes pre-labelled with name, sample ID, and date.

In order to remove secondary minerals from the samples, the weighed samples were dissolved in 15 ml of calcium acetate ((CH_3_COO)_2_Ca) buffered 1 M solution and placed on a roller rocker for 30 mins. The calcium acetate ((CH_3_COO)_2_Ca) buffered 1 M solution (pH 4.7) was prepared by mixing two solutions - the first was prepared by adding 24 ml of acetic acid (CH_3_COOH) 100% into 500 ml deionised water and the second by adding 39.54 g of (CH_3_COO)_2_Ca into a separate 500 ml deionised water. After treatment, the samples were rinsed six times with deionised water, centrifuging between washes to separate the powder and water before pipetting off the water to avoid loss of powder. The samples were placed in the freezer for 24 hours and freeze-dried for 24 hours to remove all the water and isolate apatite. The 15ml centrifuge tubes containing the treated samples were reweighed and the mass loss generated by the treatment was measured by subtracting the original weight of the tube. The weighed samples were submitted for analysis to the Iso-Analytical laboratory at Crewe Cheshire.

At the Iso-Analytical laboratory, the samples were weighed into clean Exetainer^TM^ tubes and then flushed with 99.995% helium (He). After flushing, phosphoric acid was added to the samples and they were left to react in the acid overnight to allow complete conversion of carbonate to carbon dioxide (CO_2_). Reference and control materials were prepared the same way. The CO_2_ gas liberated from samples was then analysed by Continuous Flow-Isotope Ratio Mass Spectrometry (CF-IRMS). Carbon dioxide was sampled from the Exetainer^TM^ tubes into a continuously flowing He stream using a double-holed needle. The CO_2_ was resolved on a packed column of gas chromatography and the resultant chromatographic peak carried forward into the ion source of a Europa Scientific 20-20 IRMS where it was ionised and accelerated. Gas species of different mass were separated in a magnetic field and then simultaneously measured using a Faraday cup collector array to measure the isotopomers of CO_2_ at m/z 44, 45, and 46. The phosphoric acid used for digestion had been prepared for isotopic analysis in accordance with Coplen et al. [(1983)](https://paperpile.com/c/DSwV5m/s8Tua/?noauthor=1) and was injected through the septum into the vials. Acid preparations of samples and controls were measured directly against acid preparations of the Iso-Analytical working calcium carbonate standard. This procedure removes the need to apply separate corrections for temperature-dependent isotope fractionation.

The reference material used during carbonate analysis was IA-R022 (Iso-Analytical working standard calcium carbonate, δ^13^C_V-PDB_ = -28.63‰, and δ^18^O_V-PDB_ = -22.69‰).  IA-R022, NBS-18 (carbonatite, δ^13^C_V-PDB_ = -5.01‰ and δ^18^O_V-PDB_ = -23.20‰), IA-R066 (chalk, δ^13^C_V-PDB_ = +2.33‰ and δ^18^O_V-PDB_ = -1.52‰) and ILC-1 (limestone) were run as quality control check samples during analysis of the samples. IA-R022 has been calibrated against and is traceable to NBS-18 and NBS-19 (limestone, δ^13^C_V-PDB_ = +1.95‰ and δ^18^O_V-PDB_ = -2.2‰). IA-R066 has been calibrated against and is traceable to NBS-18 and IAEA-CO-1 (carrara marble, δ^13^C_V-PDB_ = +2.5‰ and δ^18^O_V-PDB_ = -2.4‰). ILC-1 has been analysed multiple times in the Isoanalytical laboratory over a number of years. The current long-term mean value for those analyses is ẟ^13^C = 2.17‰. NBS-18, NBS-19, and IAEA-CO-1 are inter-laboratory comparison standard materials distributed by the International Atomic Energy Agency (IAEA).

**References**

Baxter, J. D., Biltz, R. M., & Pellegrino, E. D. (1966). The physical state of bone carbonate. A comparative infra-red study in several mineralized tissues. *The Yale Journal of Biology and Medicine*, *38*(5), 456–470.

Brock, F., Higham, T., & Ramsey, C. B. (2013). Comments on the Use of Ezee-Filters™ and Ultrafilters at Orau. *Radiocarbon*, *55*(1), 211-212.

Brown, T. A., Nelson, D. E., Vogel, J. S., & Southon, J. R. (1988). Improved Collagen Extraction by Modified Longin Method. *Radiocarbon*, *30*(2), 171–177.

Coplen, T. B., Kendall, C., & Hopple, J. (1983). Comparison of stable isotope reference samples. *Nature*, *302*, 236.

Hunt, J. M., Wisherd, M. P., & Bonham, L. C. (1950). Infrared Absorption Spectra of Minerals and Other Inorganic Compounds. *Analytical Chemistry*, *22*(12), 1478–1497.

Kontopoulos, I., Presslee, S., Penkman, K., & Collins, M. J. (2018). Preparation of bone powder for FTIR-ATR analysis: The particle size effect. *Vibrational Spectroscopy*, *99*, 167–177.

Kragten, J. (1994). Tutorial review. Calculating standard deviations and confidence intervals with a universally applicable spreadsheet technique. *The Analyst*, *119*(10), 2161–2165.

Longin, R. (1971). New method of collagen extraction for radiocarbon dating. *Nature*, *230*(5291), 241–242.

Pellegrini, M., & Snoeck, C. (2016). Comparing bioapatite carbonate pre-treatments for isotopic measurements: Part 2—Impact on carbon and oxygen isotope compositions. *Chemical Geology*, *420*, 88–96.

Snoeck, C., & Pellegrini, M. (2015). Comparing bioapatite carbonate pre-treatments for isotopic measurements: Part 1—Impact on structure and chemical composition. *Chemical Geology*, *417*, 394–403.

Weiner, S., & Bar-Yosef, O. (1990). States of preservation of bones from prehistoric sites in the Near East: A survey. *Journal of Archaeological Science*, *17*(2), 187–196.

Wright, L. E., & Schwarcz, H. P. (1996). Infrared and Isotopic Evidence for Diagenesis of Bone Apatite at Dos Pilas, Guatemala: Palaeodietary Implications. *Journal of Archaeological Science*, *23*(6), 933–944.
