# Supplementary material for: North and South: Exploring isotopic analysis of bone carbonates and collagen to understand post‐medieval diets in London and northern England
Source: Am J Biol Anthropol. 2023 Jul 22;182(1):126–42. doi: 10.1002/ajpa.24818 (PMC10952890; doi:10.1002/ajpa.24818)
Supplement: Supplementary file 3 — DATA S3. Supporting Information. [file AJPA-182-126-s002.docx]

**Supporting Information 3**

## **3.1 | Diet in 17^th^ to 19^th^ century England**

Post-medieval diet was predominantly cereal-based with a small meat component, this difference being more pronounced in lower socioeconomic classes who consumed considerably more bread relative to meat [(Oddy, 2000)](https://paperpile.com/c/bd5Rpe/IiDKC). The rich enjoyed greater access to foods such as meat, fish, and cane sugar due to the privilege of wealth which provided them with greater access to non-local food markets [(Barker et al., 1970; Gumerman, 1997; Oddy, 1990)](https://paperpile.com/c/bd5Rpe/D7JKd+dejgi+Vsevz). For the poor, certain foods such as cane sugar, cheese, and occasional supplies of meat were prioritised for the males who supposedly required more energy to work and as a consequence, females and children subsisted on mainly cheap bread and/or potatoes [(Burnett, 2005; Horrell & Oxley, 2012)](https://paperpile.com/c/bd5Rpe/gy03G+d2dsA). On the other hand, for the middle/upper classes, where women and men did not carry out as much labour-intensive work, both sexes in these populations consumed similar foods (Davidoff and Hall 2018). There was regional variation in meat and fish consumption, with Londoners of all social classes consuming more meat and fish than those in similar classes in the rest of the country during this period [(Thirsk, 2007; Trow-Smith, 2013)](https://paperpile.com/c/bd5Rpe/fPPSZ+196rF). However, meat products from beef, sheep, poultry and pigs were locally available in great quantities in the country [(Burnett, 2005; Clayton & Rowbotham, 2009; Scola, 1992)](https://paperpile.com/c/bd5Rpe/MGPnh+gy03G+gy33C). Livestock was brought from a wide range of places including Scotland for both London and Northern England [(Metcalfe, 2015; Trow-Smith, 2013)](https://paperpile.com/c/bd5Rpe/196rF+V6BIa).

**3.2 | Carbonate preservation**

No correlation was observed between δ^13^C and δ^18^O values in both human and animal samples (Figure S1). It has previously been established that a correlated variation of bone carbonate δ^13^C and δ^18^O can occur as a result of both uptake and incorporation of light carbon and oxygen from the surrounding environment during diagenetic alteration [(Heydari et al., 2001)](https://paperpile.com/c/bd5Rpe/sRPL). Additionally, Ullmann and Korte [(2015, p. 13)](https://paperpile.com/c/bd5Rpe/Mhpb/?locator=13&noauthor=1) also noted that post-depositional alteration almost always results in a positive correlation between δ^13^C and δ^18^O carbonates subjected to diagenesis. Therefore, the absence of a correlation between these two isotope ratios in this study strongly suggests that the isotopic signals for the samples were generally preserved. Furthermore, when FTIR data (IRSF and C/P ratio) was examined with reference to the δ^13^C and δ^18^O of bone carbonate, no correlations were observed between the parameters (Figure S2).


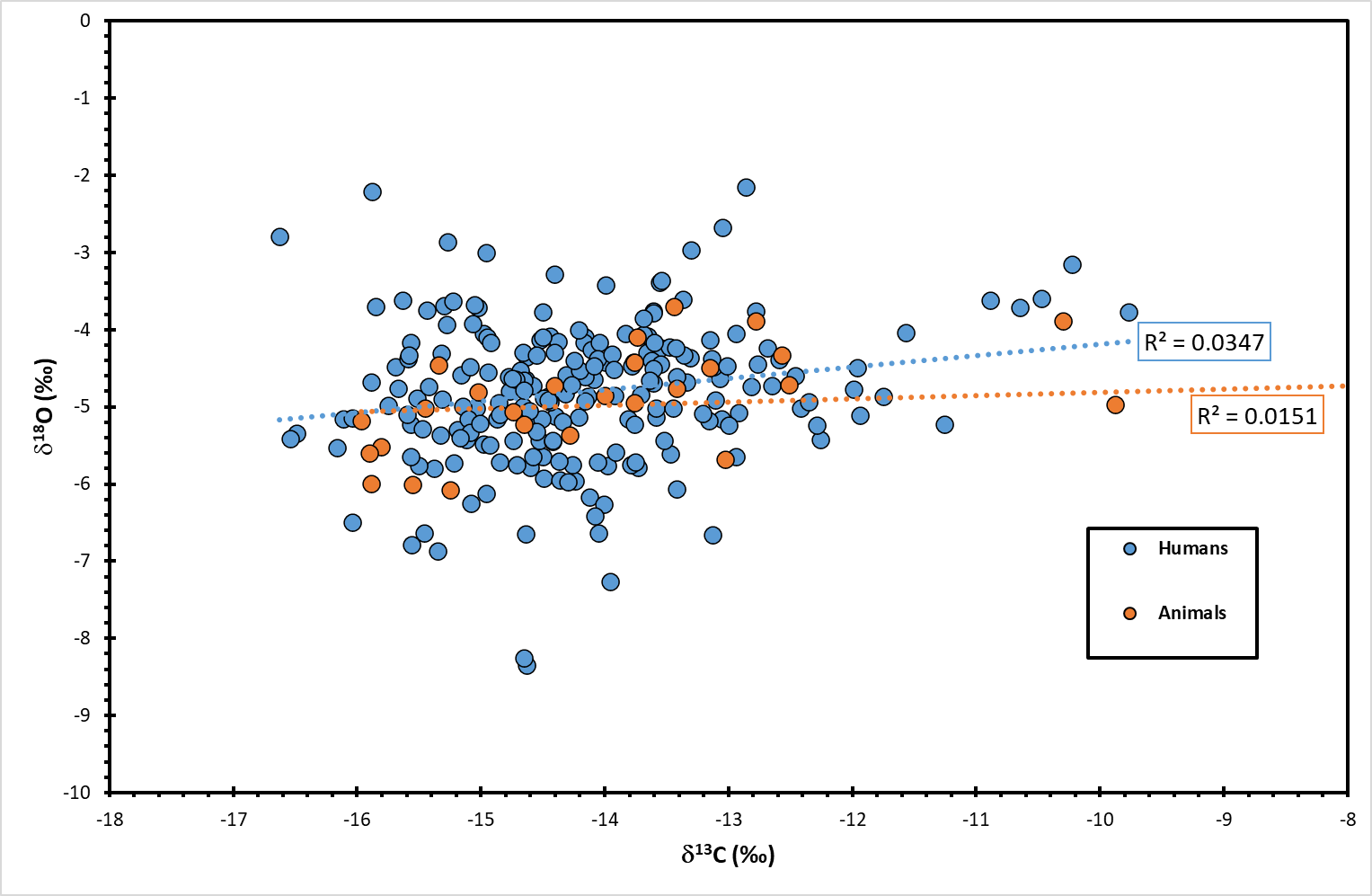


*Figure S1: The relationship between bone carbonate δ^13^C and δ^18^O in humans and animals in this study*


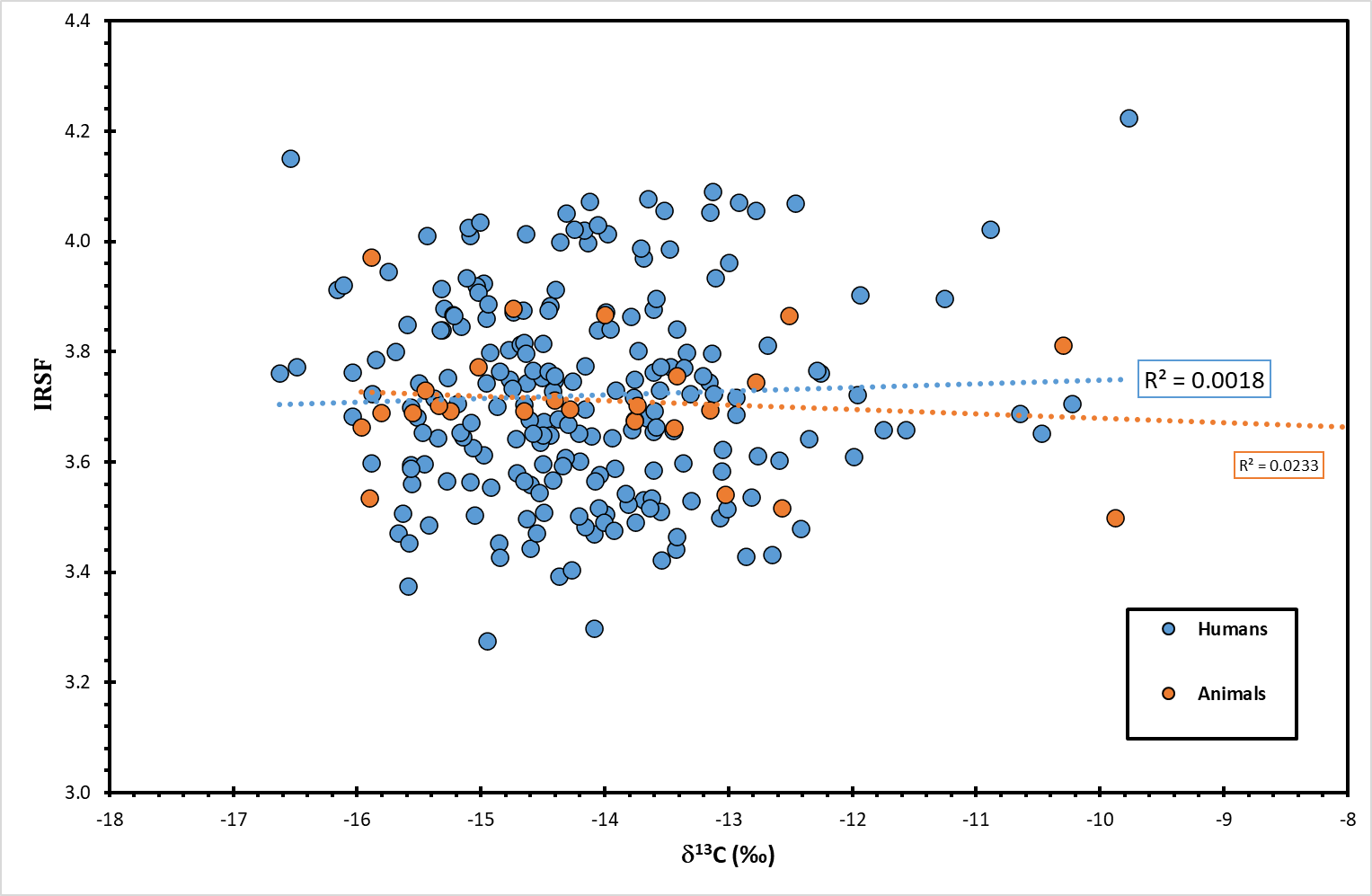


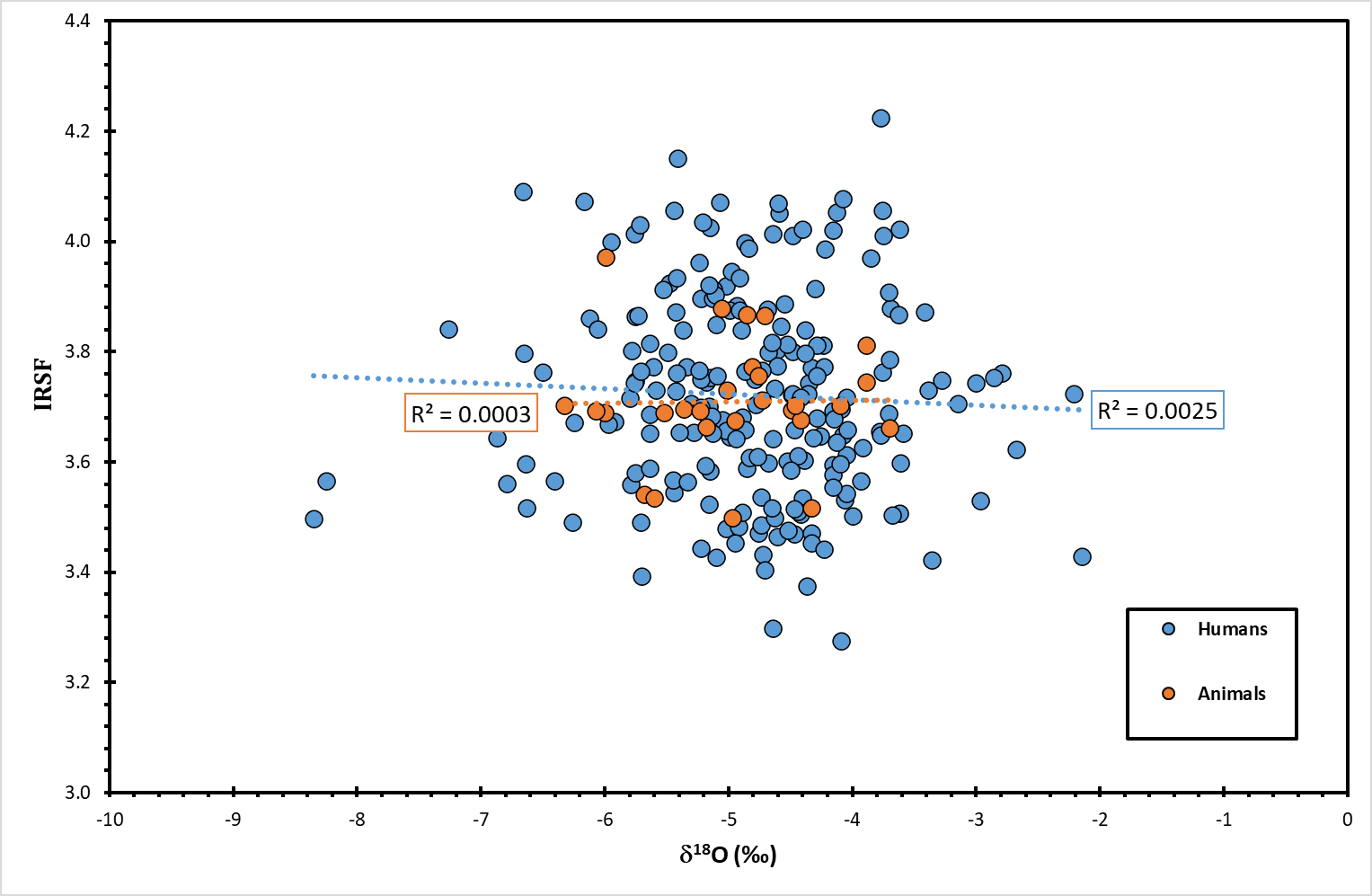


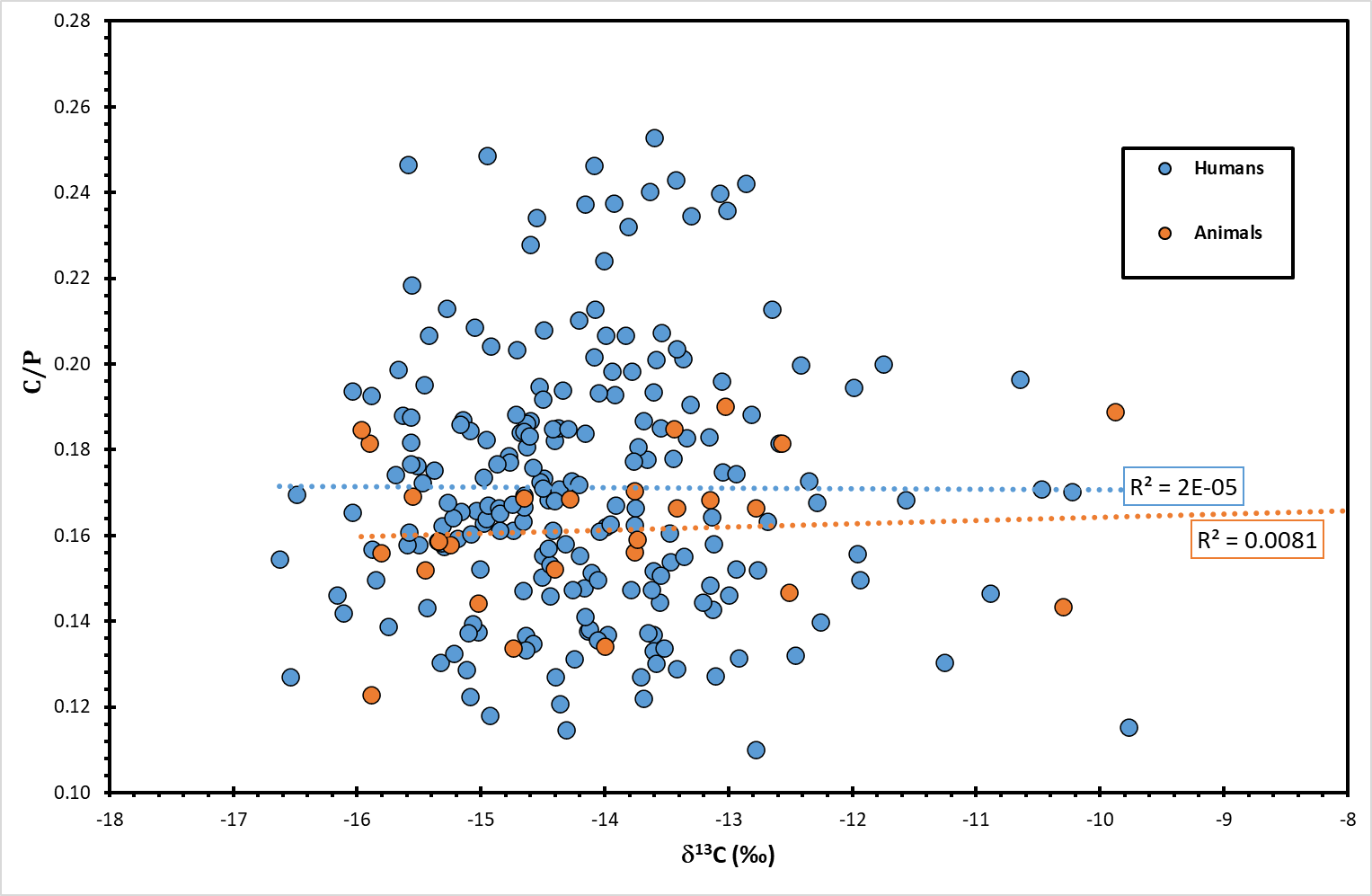


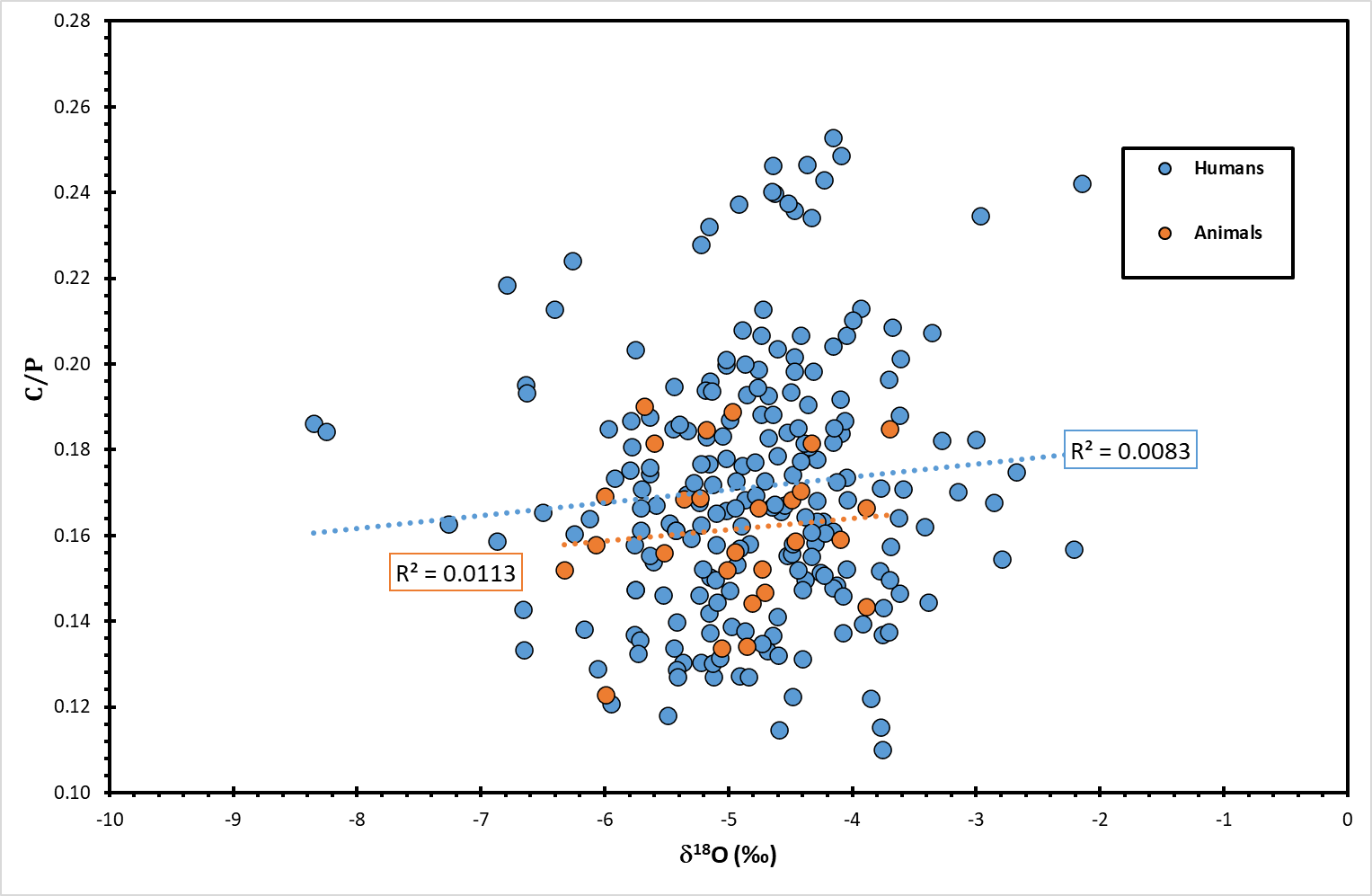


*Figure S2: The relationship between bone carbonate δ^13^C and δ^18^O with IRSF and C/P ratio in humans and animals in this study*


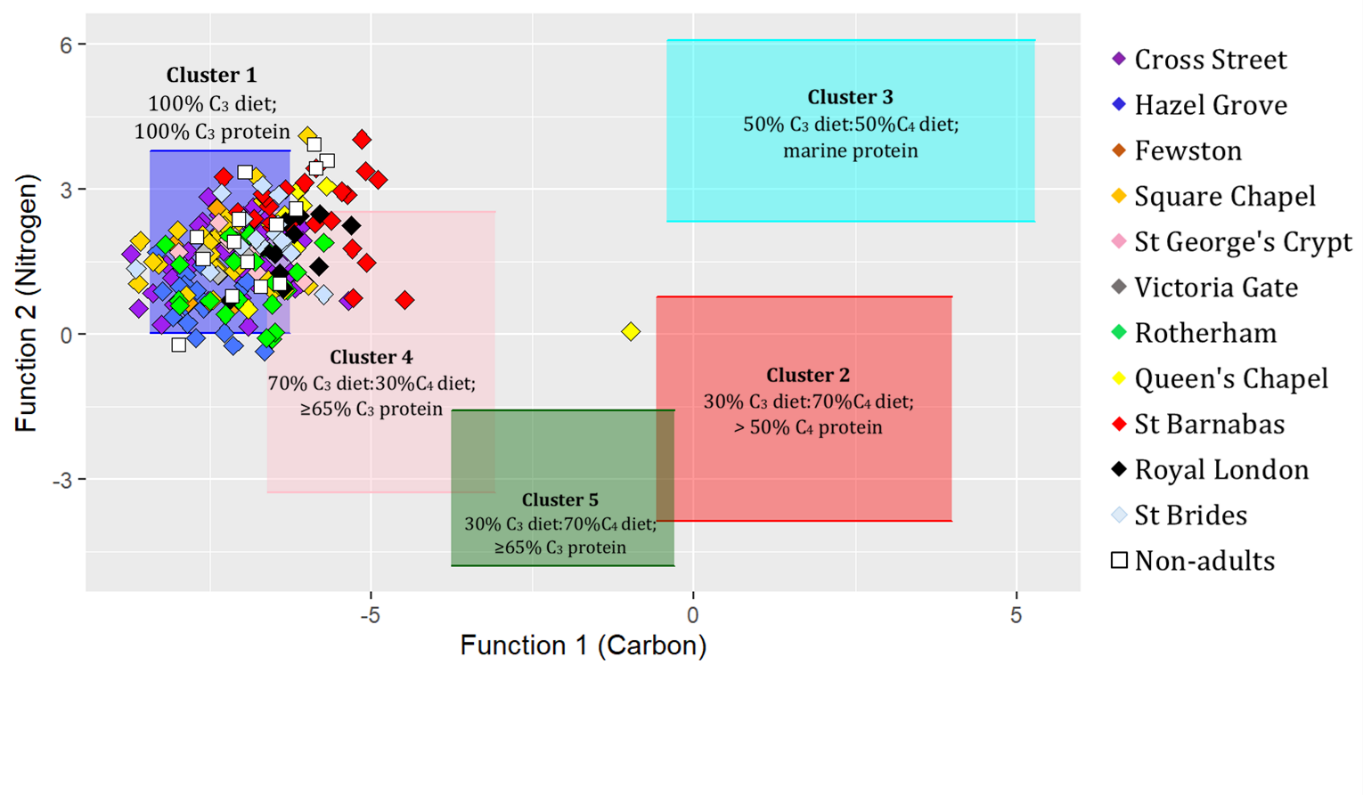


*Figure S3: F1 and F2 discriminant function values from northern and London individuals across England plotted site by site against previously generated dietary clusters (see Froehle et al., 2012)*

**References**

Barker, T. C., Oddy, D. J., & Yudkin, J. (1970). *The Dietary Surveys of Dr. Edward Smith 1862-3. A New Assessment.* London: Staples Press.

Burnett, J. (2005). *Plenty and Want: A Social History of Food in England from 1815 to the Present Day*. Routledge.

Clayton, P., & Rowbotham, J. (2009). How the mid-Victorians worked, ate and died. *International Journal of Environmental Research and Public Health*, *6*(3), 1235–1253.

Gumerman, G. (1997). Food and complex societies. *Journal of Archaeological Method and Theory*, *4*(2), 105–139.

Heydari, E., Wade, W. J., & Hassanzadeh, J. (2001). Diagenetic origin of carbon and oxygen isotope compositions of Permian–Triassic boundary strata. *Sedimentary Geology*, *143*(3), 191–197.

Horrell, S., & Oxley, D. (2012). Bringing home the bacon? Regional nutrition, stature, and gender in the industrial revolution 1. *The Economic History Review*, *65*(4), 1354–1379.

Metcalfe, R. S. (2015). *Meat, Commerce and the City: The London Food Market, 1800–1855*. Routledge.

Oddy, D. J. (1990). Food, drink, and nutrition. In F. M. L. Thompson (Ed.), *The Cambridge Social History of Britain, 1750-1950* (Vol. 2, pp. 251–278). Cambridge University Press.

Oddy, D. J. (2000). The paradox of diet and health: England and Scotland in the nineteenth and twentieth centuries in. In A. Fenton (Ed.), *Order and Disorder: The health implications of eating and drinking in the nineteenth and twentieth centuries* (pp. 45–63). Tuckwell Press Ltd.

Scola, R. (1992). *Feeding the Victorian City: The Food Supply of Manchester, 1770-1870*. Manchester: Manchester University Press.

Thirsk, J. (2007). *Food in Early Modern England: Phases, Fads, Fashions, 1500-1760*. Bloomsbury Academic.

Trow-Smith, R. (2013). *A History of British Livestock Husbandry, 1700-1900*. Routledge.

Ullmann, C. V., & Korte, C. (2015). Diagenetic alteration in low-Mg calcite from macrofossils: a review. *Geological Quarterly*, *59*(1), 3-20. <https://doi.org/10.7306/gq.1217>
