# Supplementary material for: North and South: Exploring isotopic analysis of bone carbonates and collagen to understand post‐medieval diets in London and northern England
Source: Am J Biol Anthropol. 2023 Jul 22;182(1):126–42. doi: 10.1002/ajpa.24818 (PMC10952890; doi:10.1002/ajpa.24818)
Supplement: Supplementary file 4 — DATA S4. Supporting Information. [file AJPA-182-126-s003.docx]

**Supporting Tables**

Table S1: Collagen quality indicators, Collagen and Carbonate stable isotope values for all post-medieval humans analysed in this study

| Site & Location | Sample No. | Element | Sex | Age (years) | Collagen Yield (%) | %C | %N | C:N ratio | δ^13^C_coll_ (‰) | δ^15^N (‰) | δ^13^C_carb_ (‰) | δ^18^O_carb_ (‰) |
| --- | --- | --- | --- | --- | --- | --- | --- | --- | --- | --- | --- | --- |
| Cross Street, Manchester  (Northern) | CSM 1.07 | Rib | M | 36-45 | 1.8 | 30.9 | 11.0 | 3.3 | -19.8 | 10.9 | -13.5 | -3.36 |
|  | CSM 1.12 | Rib | M | 36-45 | 4.4 | 41.0 | 14.9 | 3.2 | -19.6 | 11.5 | -15.2 | -3.63 |
|  | CSM 1.27 | Skull fragment | F | 18+ | 6.5 | 41.6 | 15.3 | 3.2 | -20.2 | 10.6 | -15.3 | -4.30 |
|  | CSM 1.37 | Rib | F | 46+ | 19.5 | 42.0 | 15.1 | 3.2 | -19.5 | 12.9 | -14.0 | -4.42 |
|  | CSM 1.41 | Rib | M | 46+ | 3.1 | 36.7 | 13.3 | 3.2 | -19.1 | 13.0 | -13.7 | -5.78 |
|  | CSM 2.05 | Fibula | U | 13-17 | 10.7 | 41.7 | 15.3 | 3.2 | -18.9 | 11.5 | -13.6 | -3.76 |
|  | CSM 2.07 | L. arm | F | 18+ | 8.2 | 43.8 | 16.0 | 3.2 | -20.4 | 10.8 | -13.7 | -4.29 |
|  | CSM 2.12 | Rib | M | 46+ | 16.4 | 41.1 | 14.7 | 3.3 | -19.6 | 11.3 | -13.6 | -3.39 |
|  | CSM 2.15 | Ribs | F | 46+ | 5.9 | 41.8 | 15.3 | 3.2 | -20.7 | 9.5 | -15.5 | -4.89 |
|  | CSM 2.16 | Fibula | M | 46+ | 14.1 | 42.3 | 15.5 | 3.2 | -20.4 | 10.0 | -14.8 | -4.61 |
|  | CSM 2.18 | Clavicle | M | 18+ | 12.6 | 40.2 | 14.8 | 3.2 | -20.4 | 9.8 | -15.7 | -4.48 |
|  | CSM 2.20 | L.ulna | F | 46+ | 8.2 | 42.3 | 15.3 | 3.2 | -20.7 | 10.6 | -16.6 | -2.79 |
|  | CSM 2.21 | Rib | F | 46+ | 18.8 | 42.5 | 15.4 | 3.2 | -19.4 | 12.3 | -14.4 | -3.28 |
|  | CSM 2.25 | Rib | F | 46+ | 17.8 | 43.7 | 16.1 | 3.2 | -19.7 | 11.5 | -15.0 | -5.48 |
|  | CSM 2.29 | Long Bone | F | 18+ | 1.6 | 41.3 | 15.0 | 3.2 | -20.5 | 11.0 | -15.0 | -5.02 |
|  | CSM 2.30 | Rib | M | 46+ | 11.1 | 42.1 | 15.1 | 3.3 | -19.4 | 12.4 | -15.0 | -3.0 |
|  | CSM 2.31 | Rib | F | 46+ | 15.0 | 42.4 | 15.4 | 3.2 | -19.7 | 12.0 | -13.1 | -5.15 |
|  | CSM 2.32 | Rib | F | 18+ | 8.7 | 46.1 | 16.7 | 3.2 | -19.7 | 12.6 | -14.2 | -4.09 |
|  | CSM 2.34 | Rib | F | 36-45 | 17.1 | 43.4 | 15.8 | 3.2 | -19.4 | 12.6 | -13.2 | -4.13 |
|  | CSM 2.35 | L.Arm | F | 18+ | 2.2 | 26.5 | 9.5 | 3.3 | -19.5 | 11.7 | -13.2 | -5.18 |
|  | CSM 2.36 | Rib | M | 18+ | 7.6 | 41.5 | 15.0 | 3.2 | -20.6 | 11.0 | -15.4 | -3.75 |
|  | CSM 2.37 | 2nd Metacarpal | M | 46+ | 12.9 | 43.7 | 15.9 | 3.2 | -19.8 | 11.9 | -14.0 | -5.76 |
|  | CSM 2.40 | Femur | U | 6.0 | 5.6 | 42.5 | 15.5 | 3.2 | -19.1 | 12.3 | -10.9 | -3.62 |
|  | CSM 2.41 | Rib | F | 26-35 | 8.1 | 43.2 | 15.6 | 3.2 | -19.6 | 12.1 | -14.6 | -4.35 |
|  | CSM 2.43 | Arm bone | M | 26-35 | 11.1 | 41.9 | 15.0 | 3.3 | -20.1 | 12.9 | -15.8 | -4.98 |
|  | CSM 2.49 | Rib | M | 18-25 | 11.3 | 41.6 | 15.3 | 3.2 | -19.6 | 12.5 | -14.7 | -5.43 |
|  | CSM 2.51 | Rib | M | 46+ | 15.3 | 43.9 | 16.0 | 3.2 | -19.7 | 12.0 | -13.5 | -5.61 |
|  | CSM 2.52 | Rib | F | 46+ | 21.5 | 45.2 | 16.1 | 3.3 | -19.3 | 12.3 | -14.6 | -5.22 |
|  | CSM 2.54 | Rib | M | 46+ | 5.5 | 43.3 | 15.2 | 3.3 | -19.5 | 12.9 | -15.0 | -4.05 |
|  | CSM 3.00 | Cranium | U | 10m-1 | 9.6 | 47.5 | 17.2 | 3.2 | -19.0 | 15.0 | -14.0 | -3.42 |
|  | CSM 3.25 | Ulna | F | 18+ | 4.0 | 43.9 | 15.7 | 3.3 | -19.9 | 11.8 | -15.3 | -4.90 |
|  | CSM 3.33 | Rib | U | 13-17 | 9.0 | 41.8 | 15.1 | 3.2 | -19.7 | 13.1 | -14.1 | -4.87 |
|  | CSM 3.34 | Rib | M | 36-45 | 15.5 | 45.3 | 16.3 | 3.2 | -20.6 | 9.5 | -14.6 | -4.64 |
|  | CSM 3.36 | Rib | U | 1-6 | 17.3 | 45.3 | 16.4 | 3.2 | -19.1 | 14.7 | -14.1 | -6.17 |
|  | CSM 3.43 | Rib | U | 13-17 | 12.1 | 43.7 | 15.6 | 3.3 | -20.1 | 11.7 | -13.6 | -3.78 |
|  | CSM 3.45 | Fibula | F | 18+ | 4.0 | 41.9 | 15.3 | 3.2 | -20.1 | 12.2 | -15.6 | -3.62 |
|  | CSM 3.48 | Rib | M | 36-45 | 17.2 | 44.2 | 15.9 | 3.2 | -20.0 | 11.9 | -15.8 | -3.70 |
|  | CSM 3.53 | Rib | F | 36-45 | 19.5 | 44.2 | 16.1 | 3.2 | -19.9 | 10.5 | -12.7 | -4.24 |
|  | CSM 4.05 | Rib | F | 36-45 | 2.7 | 30.6 | 10.8 | 3.3 | -19.9 | 11.7 | -13.7 | -4.08 |
|  | CSM 4.11 | Rib | M | 18-25 | 14.2 | 42.1 | 15.5 | 3.2 | -20.1 | 11.2 | -15.6 | -4.16 |
|  | CSM 4.12 | Rib | F | 26-35 | 31.5 | 41.5 | 15.2 | 3.2 | -19.8 | 11.9 | -14.7 | -4.29 |
|  | CSM 4.24 | Rib | M | 26-35 | 18.4 | 44.6 | 16.5 | 3.2 | -20.1 | 11.3 | -14.2 | -4.16 |
|  | CSM 4.28 | Rib | M | 36-45 | 13.4 | 42.9 | 15.6 | 3.2 | -18.5 | 12.3 | -14.6 | -5.79 |
|  | CSM 4.38 | Rib | F | 46+ | 20.1 | 43.7 | 15.3 | 3.3 | -19.5 | 12.5 | -15.2 | -4.58 |
|  | CSM 4.53 | Rib | F? | 18+ | 10.3 | 41.3 | 14.9 | 3.2 | -20.1 | 10.9 | -15.3 | -3.69 |
|  | CSM 5.05 | Rib | M | 46+ | 19.7 | 42.1 | 15.2 | 3.2 | -18.9 | 12.5 | -13.7 | -4.06 |
|  | CSM 5.07 | Ulna | F | 46+ | 2.8 | 27.8 | 10.0 | 3.2 | -20.6 | 10.8 | -15.0 | -3.71 |
|  | CSM 5.09 | Rib | M | 18+ | 8.0 | 42.2 | 15.4 | 3.2 | -19.3 | 11.7 | -12.6 | -4.39 |
|  | CSM 5.16 | Rib | M | 46+ | 12.6 | 43.5 | 15.9 | 3.2 | -19.4 | 12.5 | -13.3 | -4.68 |
|  | CSM 5.23 | Clavicle | F | 18+ | 16.1 | 42.2 | 15.3 | 3.2 | -19.5 | 11.8 | -13.1 | -2.68 |
|  | CSM 5.36 | Humerus | U | 1-6 | 5.1 | 36.8 | 13.2 | 3.3 | -19.5 | 11.7 | -12.9 | -4.05 |
|  | CSM 61.03 | Metacarpal | U | 36-45 | 17.8 | 43.7 | 15.9 | 3.2 | -19.2 | 12.7 | -14.1 | -4.64 |
|  | CSM 61.04 | Femur | U | 1-6 | 12.5 | 41.8 | 15.3 | 3.2 | -19.0 | 15.1 | -14.7 | -4.53 |
|  | CSM 62.02 | Rib | F | 26-35 | 19.9 | 43.0 | 15.8 | 3.2 | -20.2 | 12.8 | -15.0 | -6.12 |
| Hazel Grove, Stockpot  (Northern) | HGM 1 | Fibula | M? | 26-35 | 9.8 | 45.9 | 16.9 | 3.2 | -20.4 | 10.2 | -14.4 | -5.12 |
|  | HGM 2 | Tibia | U | 36-40wiu | 8.5 | 45.9 | 16.5 | 3.2 | -20 | 11.6 | -15.7 | -4.76 |
|  | HGM 3 | Rib | U | 6-8 | 13.6 | 46.4 | 17.1 | 3.2 | -20.1 | 10.2 | -14.2 | -4.61 |
|  | HGM 4 | Rib | U | 12-14 | 9.3 | 46.3 | 16.9 | 3.2 | -20 | 10.2 | -14.3 | -4.59 |
|  | HGM 5 | Rib | U | 1-2 | 16.1 | 47.2 | 17.1 | 3.2 | -19.8 | 13.9 | -15.4 | -4.74 |
|  | HGM 6 | Rib | F | 46+ | 13.3 | 45.8 | 16.8 | 3.2 | -20.4 | 11.5 | -14.5 | -5.64 |
|  | HGM 8 | Tibia | U | 6m -2 | 5.4 | 46.4 | 16.6 | 3.3 | -19.4 | 12.9 | -14.1 | -4.26 |
|  | HGM 9 | Femur | U | 0-1 | 11.4 | 43.3 | 15.7 | 3.2 | -19.6 | 13.9 | -13.4 | -4.33 |
|  | HGM 10 | Rib | F | 36-45 | 14.9 | 46.8 | 17.3 | 3.2 | -20.2 | 10.3 | -15.2 | -5.30 |
|  | HGM 11 | Femur | U | 0-3m | 13.6 | 46.3 | 16.8 | 3.2 | -19.5 | 11.7 | -15.1 | -4.48 |
|  | HGM 12 | Humerus | M? | 18+ | 8.1 | 43.3 | 15.7 | 3.2 | -20.5 | 9.8 | -14.5 | -5.15 |
|  | HGM 13 | Petrous | U | 0m | 3 | 41.8 | 14 | 3.5 | -20.5 | 11.8 | -14.4 | -5.95 |
|  | HGM 15 | Rib | M | 36-45 | 6.4 | 44.5 | 16 | 3.2 | -20.2 | 11.2 | -13.1 | -4.91 |
|  | HGM 16 | Tibia | U | 18+ | 8.5 | 44.4 | 15.9 | 3.3 | -20.6 | 10.3 | -15.1 | -3.92 |
|  | HGM 17 | Tibia | U | 4-6 | 9.5 | 45.3 | 16.1 | 3.3 | -20.6 | 9.4 | -13.7 | -3.85 |
|  | HGM 19 | Rib | U | 8-10 | 7.7 | 43.7 | 15.8 | 3.2 | -20.7 | 10.2 | -13.6 | -4.69 |
|  | HGM 21 | Rib | M | 46+ | 9.4 | 42.8 | 15.4 | 3.2 | -20.5 | 11.4 | -12.8 | -4.74 |
|  | HGM 22 | Rib | F | 36-45 | 6.2 | 42.9 | 15.6 | 3.2 | -20.4 | 10.7 | -14.2 | -4.53 |
|  | HGM 23 | Tibia | U | 7-9m | 9.8 | 42.4 | 15.3 | 3.2 | -19.5 | 10.4 | -14.3 | -4.83 |
|  | HGM 24 | Rib | U | 2-4 | 12.7 | 44 | 16 | 3.2 | -19.9 | 11.7 | -12.9 | -5.64 |
|  | HGM 26 | Rib | F | 46+ | 9.4 | 43.6 | 16 | 3.2 | -20.2 | 10.6 | -11.3 | -5.22 |
|  | HGM 27 | Tibia | U | 18+ | 4.9 | 42.7 | 15.4 | 3.2 | -19.7 | 11.1 | -13.8 | -5.22 |
|  | HGM 28 | Rib | U | 5-7 | 11.1 | 43.2 | 15.8 | 3.2 | -20.5 | 9.8 | -13.4 | -6.06 |
|  | HGM 29 | Radius | U | 18+ | 6.6 | 45.8 | 16.5 | 3.2 | -20.6 | 11.1 | -15.9 | -2.21 |
|  | HGM 30 | Fibula | U | 18+ | 7 | 44.4 | 16.2 | 3.2 | -20.1 | 10.6 | -15.3 | -5.37 |
|  | HGM 31 | Femur | U | 2-4 | 6.7 | 42.9 | 15.5 | 3.2 | -20 | 12.1 | -13.6 | -4.23 |
|  | HGM 32 | Rib | M | 46+ | 10.3 | 43.3 | 15.7 | 3.2 | -20.3 | 10.3 | -12.8 | -3.76 |
|  | HGM 33 | Rib | F | 36-45 | 9 | 47.2 | 17.3 | 3.2 | -20.3 | 10.2 | -12.3 | -5.42 |
|  | HGM 34 | Tibia | U | 18+ | 7.8 | 42.2 | 15.2 | 3.2 | -19.8 | 11.4 | -13.6 | -5.13 |
|  | HGM 35 | Fibula | U | 18+ | 5.3 | 44.4 | 16 | 3.2 | -20.5 | 10.8 | -14.6 | -4.73 |
|  | HGM 36 | Rib | F | 60+ | 4.9 | 42.3 | 15.5 | 3.2 | -20.3 | 10.7 | -14.4 | -4.08 |
| Fewston, Harrogate  (Northern) | FEW 53 | Rib | F | 46+ | - | - | - | 3.2 | -20.1 | 10.7 | -13.3 | -4.36 |
|  | FEW 77 | Rib | M | 17-20 | - | - | - | 3.3 | -20.7 | 10.7 | -13.9 | -4.85 |
|  | FEW 130 | Rib | M | 66 | - | - | - | 3.2 | -19.3 | 11.9 | -16.5 | -5.34 |
|  | FEW 156 | Rib | F? | 66? | - | - | - | 3.2 | -20.3 | 10.7 | -13.8 | -5.16 |
|  | FEW 177 | Rib | F | 36-35 | - | - | - | 3.2 | -19.8 | 12.2 | -14.5 | -5.92 |
|  | FEW 238 | Rib | F | 49 | - | - | - | 3.2 | -20.5 | 11.5 | -15.6 | -4.37 |
|  | FEW 241 | Rib | M | 46+? | - | - | - | 3.2 | -20.3 | 11.9 | -14.7 | -5.75 |
| Square Chapel, Halifax  (Northern) | SCH 35 | Rib | M | 46+ | 16.1 | 43.1 | 15.7 | 3.2 | -19.6 | 12.2 | -15.6 | -6.79 |
|  | SCH 39 | Rib | F | 46+ | 13.1 | 45.5 | 16.7 | 3.2 | -19.7 | 13.5 | -14.5 | -4.89 |
|  | SCH 59 | Rib | F | 46+ | 15.2 | 46 | 17.1 | 3.1 | -18.7 | 14.8 | -15.5 | -6.64 |
|  | SCH 80 | Rib | U | 3 | 12.1 | 42.9 | 15.9 | 3.2 | -19.3 | 12.1 | -15.6 | -5.22 |
|  | SCH 88 | Ulna | M | 46+ | 7.6 | 44.5 | 16.4 | 3.2 | -19.7 | 12.1 | -15.4 | -5.80 |
|  | SCH 92 | Rib | F | 17-22 | 10.7 | 45.3 | 16.5 | 3.2 | -19.7 | 11.7 | -15.5 | -5.76 |
|  | SCH 98 | Humerus | M | 18+ | 13.4 | 42 | 15.2 | 3.2 | -19.7 | 13.9 | -15.1 | -5.42 |
|  | SCH 119 | Fibula | M | 18+ | 9.4 | 45.3 | 16.4 | 3.2 | -19.6 | 11.9 | -15.4 | -6.87 |
|  | SCH 140 | Femur | F | 36-45 | 11.3 | 44.8 | 16.4 | 3.2 | -19.8 | 12.1 | -14.6 | -8.25 |
|  | SCH 191 | Rib | M | 18+ | 5.9 | 44.1 | 16.1 | 3.2 | -19.8 | 12.4 | -14.9 | -5.49 |
|  | SCH 1144 | Left medial clavicle | F | 18+ | 4.3 | 40.7 | 15 | 3.2 | -19.8 | 11.9 | -12.9 | -5.07 |
|  | SCH 1146 | 1st right rib | F | 26-35 | 9.6 | 41.2 | 15.2 | 3.2 | -19.5 | 11.4 | -13 | -5.24 |
|  | SCH 1171 | Left proximal radius | M | 36-45 | 5.9 | 41.6 | 15.4 | 3.2 | -20.5 | 10.6 | -14.4 | -5.45 |
|  | SCH 1202 | 2nd right rib | M | 45+ | 5 | 41.6 | 15.1 | 3.2 | -19.3 | 11.5 | -14.6 | -5.05 |
|  | SCH 1232 | 1st right rib | M | 36-45 | 8.7 | 41.3 | 15.2 | 3.2 | -20 | 12.2 | -14.6 | -5.64 |
|  | SCH 1247 | Right distal radius | M | 26-35 | 1.5 | 37.9 | 13.7 | 3.2 | -20.3 | 12.1 | -15.1 | -5.15 |
|  | SCH 1268 | 1st left rib | M | U | 9.7 | 41.8 | 15.3 | 3.2 | -19.1 | 11.8 | -12.5 | -4.60 |
|  | SCH 1329 | 2nd left rib | M | 36-45 | 2.1 | 41.1 | 15 | 3.2 | -19.4 | 12.6 | -13.5 | -5.02 |
|  | SCH 1347 | Left medial clavicle | F? | 18+ | 6.5 | 42.2 | 15.4 | 3.2 | -19.9 | 11.3 | -15.1 | -5.33 |
|  | SCH 1357 | 2nd right rib | F | 26-35 | 11.6 | 41.8 | 15.4 | 3.2 | -20.1 | 11.4 | -14.4 | -5.43 |
|  | SCH 1377 | 2nd left rib | F | 18-25 | 11.5 | 44.6 | 16.3 | 3.2 | -19.8 | 11.6 | -14.1 | -5.72 |
|  | SCH 1381 | Left ulna midshaft | M | 18+ | 3.5 | 40.3 | 14.8 | 3.2 | -20.3 | 10.5 | -16.1 | -5.16 |
|  | SCH 1384 | 2nd left rib | M | 18-25 | 16.2 | 43.6 | 15.9 | 3.2 | -20 | 11.3 | -14.5 | -4.91 |
|  | SCH 1415 | Left radius midshaft | M | 18+ | 7.5 | 42.7 | 15.1 | 3.3 | -20.7 | 11.1 | -16.5 | -5.41 |
|  | SCH 1470 | 11th left rib | M | 36-45 | 16.2 | 42.3 | 15.4 | 3.2 | -19.8 | 11.5 | -15.5 | -5.28 |
|  | SCH 1525 | 1st right rib | F | 18-25 | 12.4 | 42 | 15.3 | 3.2 | -20.1 | 11.6 | -14.8 | -5.71 |
|  | SCH 1546 | 2nd right rib | M | 18-25 | 11.5 | 41.2 | 15.1 | 3.2 | -20.8 | 11 | -15.6 | -5.64 |
|  | SCH 1159 | Humerus | U | 18+ | 13.5 | 36.8 | 13.4 | 3.2 | -19.9 | 11.2 | -15 | -5.21 |
|  | SCH 1337 | Ulna | U | 18+ | 19 | 39.9 | 14.3 | 3.3 | -20.9 | 10.3 | -15.6 | -5.10 |
|  | SCH 1452 | Metatarsal | U | U | 22.7 | 38.5 | 14.1 | 3.2 | -20.1 | 10.6 | -14 | -6.63 |
|  | SCH 1482 | Rib | U | 18+ | 24.4 | 39.8 | 14.5 | 3.2 | -20 | 11.6 | -13.9 | -5.59 |
|  | SCH 1513 | Rib | U | 18+ | 15.2 | 41.3 | 14.8 | 3.3 | -20.3 | 11.7 | -16 | -6.50 |
| St George’s Crypt, Leeds  (Northern) | SGC 1003 | Metatarsal | U | 18+ | 19.5 | 43.2 | 15.8 | 3.2 | -20 | 12.4 | -15.2 | -5.73 |
|  | SGC 1006 | Metatarsal | U | 18+ | 19.4 | 43.6 | 15.9 | 3.2 | -18.5 | 11.1 | -13.6 | -4.40 |
|  | SGC 1010 | Metatarsal | U | 18+ | 22.5 | 44.1 | 16 | 3.2 | -20 | 12.4 | -14.4 | -5.70 |
|  | SGC 1014 | Hand Phalanx | F? | 18-25 | 20.2 | 43.6 | 16 | 3.2 | -19.6 | 11.6 | -13.7 | -5.71 |
|  | SGC 1017 | Metatarsal | M? | 36-45 | 21.3 | 43.3 | 15.8 | 3.2 | -19.6 | 11.9 | -14.9 | -4.95 |
|  | SGC 1020 | Hand Phalanx | M | 36-45 | 17.3 | 44.4 | 16.1 | 3.2 | -19.8 | 11.9 | -14.8 | -5.10 |
|  | SGC 1024 | Hand Phalanx | M | 18-25 | 19.7 | 43.1 | 15.8 | 3.2 | -19.5 | 11.8 | -14.3 | -4.71 |
|  | SGC 1029 | Hand Phalanx | M | 46+ | 19.7 | 42.8 | 15.6 | 3.2 | -20.3 | 11.2 | -15.6 | -4.33 |
|  | SGC 5003 | Hand Phalanx | F | 46+ | 20.4 | 41.9 | 15.2 | 3.2 | -20 | 12.2 | -14.6 | -6.65 |
| Victoria Gate, Leeds  (Northern) | VGL 12 | Rib | F? | 16-19 | - | 42.9 | 15.7 | 3.2 | -19.8 | 12 | -14 | -6.26 |
|  | VGL 2 | Rib | U | 5-6 | - | 43 | 15.6 | 3.2 | -20.4 | 11.6 | -13.8 | -4.42 |
|  | VGL6 | Rib | U | 7-8 | - | 43.4 | 16 | 3.2 | -20.2 | 11.6 | -14.9 | -4.16 |
| Rotherham Minster, Rotherham  (Northern) | ROM 3 | Tibia shaft | U | 18+ | 10.4 | 44.5 | 16.1 | 3.2 | -20.7 | 11.4 | -14.8 | -4.79 |
|  | ROM 7 | Left rib | F | 18-20 | 10.6 | 43 | 15.5 | 3.2 | -20.3 | 11.1 | -16.2 | -5.53 |
|  | ROM 9 | Right rib | M | 18+ | 9.2 | 43.4 | 15.4 | 3.3 | -20.4 | 10.2 | -14.7 | -4.65 |
|  | ROM 11 | Right phalanx | M? | 46+ | 1.4 | 42.3 | 15.2 | 3.3 | -20.1 | 10.5 | -14.1 | -4.38 |
|  | ROM 13 | Rib | M | 36-45 | 6.3 | 42.5 | 15 | 3.3 | -19.5 | 12.2 | -14.7 | -4.99 |
|  | ROM 17 | Right temporal | F? | 18+ | 8.4 | 42.9 | 15.5 | 3.2 | -20 | 10.4 | -13.5 | -5.44 |
|  | ROM 23 | Ulna | F | 18-25 | 5.6 | 43.3 | 15.5 | 3.2 | -19.8 | 11.4 | -12.4 | -5.02 |
|  | ROM 24 | Radius | F | 46+ | 19.5 | 43.2 | 15.8 | 3.2 | -19.3 | 12.1 | -12.8 | -4.44 |
|  | ROM 25 | Humerus | M | 26-35 | 20.2 | 42.6 | 15.6 | 3.2 | -18.8 | 12.8 | -13.1 | -4.63 |
|  | ROM 28 | Rib | F | 26-35 | 26.8 | 42.7 | 15.6 | 3.2 | -19.7 | 10.7 | -12 | -4.49 |
|  | ROM 31 | Rib | F? | 46+ | 10.4 | 42.7 | 15.4 | 3.2 | -19.8 | 10.8 | -13.6 | -5.02 |
|  | ROM 35 | Humerus | M? | 18+ |  | 42.5 | 15.6 | 3.2 | -19.9 | 10.7 | -11.7 | -4.87 |
|  | ROM 37 | Rib | F | 46+ | 14.3 | 43.6 | 15.6 | 3.3 | -20.3 | 12.7 | -13.8 | -5.75 |
|  | ROM 44 | Femur | F | 46+ | 11.5 | 43.1 | 15.4 | 3.3 | -19.8 | 12.1 | -14.9 | -5.16 |
|  | ROM 47 | Rib | M | 36+ | 15 | 42.3 | 15.5 | 3.2 | -20.4 | 10.1 | -14.6 | -5.32 |
|  | ROM 51 | Right distal tibia | F | 46+ | 4.5 | 42.4 | 14.7 | 3.4 | -19.9 | 11.7 | -14.2 | -5.96 |
|  | ROM 55 | Rib | M | 46+ | 9 | 43.1 | 15.7 | 3.2 | -19.7 | 11.9 | -13.8 | -4.05 |
|  | ROM 56 | Radius | M | 18-21 | 5 | 42 | 15.4 | 3.2 | -19.4 | 11.6 | -12.7 | -4.72 |
|  | ROM 57 | Radius | F | 46+ | 23.7 | 43.7 | 15.8 | 3.2 | -19.5 | 12.2 | -14 | -7.26 |
|  | ROM 58 | Radius | M | 36-45 | 15.1 | 42.3 | 15.5 | 3.2 | -19.5 | 11.6 | -13.1 | -4.48 |
|  | ROM 60 | Rib | M | 26-35 | 23 | 42.6 | 15.6 | 3.2 | -19.9 | 10.6 | -11.9 | -5.11 |
| Queen’s Chapel Savoy, London | QCS 122 | Rib | F | 18-30 | 13.3 | 41.2 | 15.3 | 3.1 | -19.2 | 10.1 | -14 | -4.16 |
|  | QCS 124 | Rib | F? | 45+ | 11 | 41.5 | 15.4 | 3.2 | -18.7 | 12.5 | -15 | -4.09 |
|  | QCS 163 | Rib | F | 18+ | 2.2 | 36 | 12.9 | 3.3 | -19.2 | 11.3 | -13.1 | -6.66 |
|  | QCS 534 | Rib | F | 45+ | 5.9 | 42.9 | 15.7 | 3.2 | -19.1 | 12.4 | -13.5 | -4.22 |
|  | QCS 589 | Rib | M | 45+ | 5.9 | 44.4 | 16.2 | 3.2 | -19 | 13.7 | -14.5 | -5.44 |
|  | QCS 1123 | Mandible | M | 31-45 | 15.1 | 43.8 | 16.2 | 3.1 | -13.1 | 10.7 | -9.8 | -3.77 |
|  | QCS 1804 | Rib | F | 31-45 | 15.7 | 43.7 | 16.1 | 3.2 | -18.8 | 13.2 | -14.4 | -4.93 |
|  | QCS 1810 | Rib | F | 45+ | 5.7 | 43.2 | 15.9 | 3.2 | -19 | 12.3 | -14.9 | -4.55 |
|  | QCS 1817 | Rib | F | 45+ | 13.3 | 43.3 | 15.9 | 3.2 | -18.8 | 12.8 | -14.5 | -4.13 |
|  | QCS 1998 | Rib | M? | 31-45 | 13 | 45.5 | 16.7 | 3.2 | -19 | 14.4 | -13.6 | -4.16 |
| St Barnabas/St Mary Abbots, London | SBK 7 | Metacarpal | F | 81 | 7.5 | 38 | 13.9 | 3.2 | -19.6 | 12.6 | -15.3 | -2.86 |
|  | SBK 8 | Mandible | M | 71 | 1.3 | 42 | 15.3 | 3.2 | -19.8 | 13.3 | -13.7 | -4.84 |
|  | SBK 9 | Metacarpal | F | 57 | 9.4 | 41.5 | 15 | 3.3 | -19.3 | 12.7 | -10.5 | -3.59 |
|  | SBK 10 | Metacarpal | M | 42 | 2.5 | 43.3 | 14.6 | 3.3 | -19 | 14 | -14.4 | -4.15 |
|  | SBK 11 | Metacarpal | M | 64 | 2.2 | 43.5 | 15.4 | 3.3 | -19.2 | 13.5 | -13.1 | -4.38 |
|  | SBK 12 | Metacarpal | M | 71 | 5.3 | 43.7 | 15.2 | 3.3 | -19.3 | 13.2 | -15.2 | -4.99 |
|  | SBK 15 | Metacarpal | M | 48 | 10.8 | 43.6 | 15.4 | 3.3 | -19.3 | 13.8 | -14.5 | -4.10 |
|  | SBK 17 | Metacarpal | M | 81 | 8 | 45.6 | 16.5 | 3.3 | -18.3 | 12.6 | -10.2 | -3.15 |
|  | SBK 18 | Metacarpal | M | 55 | 8.3 | 48.2 | 17 | 3.3 | -18.1 | 14.6 | -13.3 | -2.97 |
|  | SBK 21 | Rib | M | 73 | 11.3 | 44.6 | 15.9 | 3.3 | -19.3 | 13.8 | -10.6 | -3.71 |
|  | SBK 26 | Metacarpal | F | 43 | 9.4 | 43.5 | 15.8 | 3.3 | -19.2 | 12.5 | -14.5 | -4.87 |
|  | SBK 30 | Rib | M | 18 | 14.7 | 44.6 | 16 | 3.3 | -19 | 13.7 | -12.9 | -2.15 |
|  | SBK 34 | Metacarpal | M | 83 | 11.9 | 42.7 | 15.4 | 3.3 | -18.4 | 13.9 | -13.9 | -4.32 |
|  | SBK 36 | Metacarpal | M | 18+ | 9.4 | 43.1 | 15.9 | 3.3 | -17.8 | 14.2 | -14.3 | -5.75 |
|  | SBK 43 | Rib | F | 46 | 11.1 | 42.8 | 15.5 | 3.3 | -19.1 | 13.6 | -11.6 | -4.04 |
|  | SBK 44 | Metacarpal | F | 74 | 12.4 | 44.8 | 15.8 | 3.3 | -18.1 | 15.2 | -14.5 | -3.77 |
|  | SBK 45 | Metacarpal | F | 29 | 11.7 | 45.9 | 16.4 | 3.3 | -19.6 | 13.4 | -13.4 | -3.61 |
|  | SBK 46 | Metacarpal | F | 44 | 5.8 | 43.1 | 15.8 | 3.2 | -19.6 | 13.5 | -14.6 | -8.35 |
|  | SBK 48 | Metacarpal | F | 72 | 8.9 | 40.9 | 14.9 | 3.2 | -18.1 | 13.6 | -14.1 | -4.47 |
|  | SBK 53 | Metacarpal | M | 41 | 12 | 42.9 | 15.6 | 3.3 | -19.7 | 12.9 | -14.5 | -4.33 |
|  | SBK 54 | Rib | F | 40 | 9.2 | 44.7 | 16.2 | 3.3 | -19.7 | 13.5 | -14.2 | -4.92 |
|  | SBK 57 | Metacarpal | F | 76 | 10.1 | 43.7 | 14.9 | 3.4 | -20.3 | 13.9 | -15.3 | -3.93 |
|  | SBK 58 | Metacarpal | M | 51 | 2.6 | 41.2 | 14.8 | 3.2 | -18.5 | 14 | -15 | -3.68 |
| Royal London Hospital, London | RLH 103 | Rib | F | 26-35 | 10 | 38.8 | 14.2 | 3.2 | -19.2 | 12.6 | -12.3 | -5.24 |
|  | RLH 135 | Rib | M | 36-45 | 6.1 | 35.1 | 12.7 | 3.2 | -19.6 | 12.4 | -13.4 | -4.23 |
|  | RLH 208 | Rib | M? | 36-45 | 7.7 | 38.2 | 14 | 3.2 | -19.1 | 12.7 | -13.8 | -4.47 |
|  | RLH 340 | Rib | M? | 36-45 | 4.7 | 38.5 | 13.7 | 3.3 | -18.3 | 13.2 | -13.2 | -5.09 |
|  | RLH 349 | Rib | M | 36-45 | 17.1 | 39.8 | 14.6 | 3.2 | -18.9 | 13 | -14.2 | -5.13 |
|  | RLH 356 | Rib | M? | 18+ | 6.8 | 35.9 | 12.9 | 3.2 | -19.7 | 11.9 | -12.4 | -4.94 |
|  | RLH 367 | Rib | M | 36-45 | 2.6 | 41.5 | 15.1 | 3.2 | -19.1 | 12.8 | -14.3 | -5.19 |
|  | RLH 386 | Rib | F? | 36-45 | 5.7 | 37 | 13.4 | 3.2 | -19.2 | 11.6 | -13.5 | -4.44 |
|  | RLH 397 | Rib | F | 36-45 | 15.6 | 32.7 | 11.9 | 3.2 | -19 | 13.6 | -13.4 | -4.61 |
|  | RLH 421 | Rib | M | 36-45 | 7 | 36 | 12.9 | 3.3 | -20.4 | 11.3 | -13 | -4.47 |
|  | RLH 572 | Rib | M | 36-45 | 4.3 | 43 | 15.7 | 3.2 | -19.6 | 12.3 | -13.9 | -4.52 |
| St Brides Lower, London | SBL 1203 | Rib | F | 46+ | 18.5 | 43.6 | 15.9 | 3.2 | -19.1 | 12.3 | -14.1 | -6.41 |
|  | SBL 1207 | Rib | F | 26-35 | 18.8 | 41.5 | 15.3 | 3.2 | -19.3 | 12.8 | -14.2 | -4.00 |
|  | SBL 1215 | Rib | F? | 36-45 | 11.1 | 41.8 | 15.3 | 3.2 | -20.2 | 11.3 | -14.4 | -4.29 |
|  | SBL 1244.1 | Rib | M | 18+ | 19.6 | 43.4 | 16 | 3.2 | -19.5 | 12.2 | -14.2 | -4.40 |
|  | SBL 1526 | Rib | M | 26-35 | 17.8 | 42.2 | 15.6 | 3.2 | -19.4 | 13.5 | -15.2 | -5.40 |
|  | SBL 1558 | Rib | M | 36-45 | 10.9 | 40.3 | 14.8 | 3.2 | -19.2 | 12.4 | -14.3 | -5.97 |
|  | SBL 1641 | Rib | F | 36-45 | 17 | 43 | 15.8 | 3.2 | -19.3 | 11.5 | -15.1 | -6.25 |
|  | SBL 1653 | Rib | F | 26-35 | 16.8 | 43 | 15.9 | 3.2 | -19.3 | 12 | -14.7 | -4.64 |
|  | SBL 1785 | Rib | M | 46+ | 9.2 | 41.2 | 14.9 | 3.2 | -19.1 | 12.2 | -13.6 | -4.50 |
|  | SBL 1799 | Rib | F | 36-45 | 13.6 | 42.4 | 15.7 | 3.1 | -19.2 | 13.4 | -14.8 | -4.63 |
|  | SBL 1872 | Rib | M | 36-45 | 16.7 | 42.2 | 15.6 | 3.1 | -19.4 | 12.6 | -13.6 | -4.65 |
|  | SBL 1932 | Rib | M | 36-45 | 18.1 | 43.8 | 16.1 | 3.2 | -19.7 | 12.8 | -16 | -5.14 |
|  | SBL 2049 | Rib | F | 36-45 | 18.4 | 43.7 | 15.9 | 3.2 | -20.9 | 10.6 | -15.9 | -4.68 |
|  | SBL 2134 | Rib | F | 26-35 | 12.8 | 42.6 | 15.4 | 3.2 | -19.8 | 12.8 | -14.7 | -4.78 |
|  | SBL 2296 | Rib | M | 46+ | 19.4 | 42.3 | 15.7 | 3.2 | -19 | 11.8 | -12 | -4.77 |

**Sex category**: U =Sex not determined, F = female, F? = probably female, M =Male, M? = probably male

**Age category**: U = age not determined, m= months, wiu=weeks in utero

**Data**: -= No data available

Table S2: Collagen quality indicators, Collagen and Carbonate stable isotope values for all animal remains in this study

| Site | Period | Sample | Taxon | Element sampled | Coll Yield (%) | %C | %N | C:N (ratio) | δ^13^C_coll_ (‰) | δ^15^N (‰) | δ^13^C_carb_ (‰) | δ^18^O_carb_ (‰) |
| --- | --- | --- | --- | --- | --- | --- | --- | --- | --- | --- | --- | --- |
| Cross Street, Manchester  (Northern) | 17^th^ -19^th^C | CSM 2030 | Cattle | Rib | 17.4 | 39.2 | 14.6 | 3.1 | -22.2 | 7.0 | - | - |
|  | 17^th^ -19^th^C | CSM 2050 | Mallard | Femur | 17.0 | 38.3 | 14.1 | 3.2 | -21.9 | 10.9 | - | - |
|  | 17^th^ -19^th^C | CSM 2051-2 | Cattle | Rib | 16.0 | 38.1 | 14.4 | 3.1 | -22.7 | 6.4 | - | - |
|  | 17^th^ -19^th^C | CSM 2056 | Sheep | Tibia | 6.9 | 40.7 | 14.9 | 3.2 | -21.9 | 6.5 | -15.0 | -4.81 |
|  | 17^th^ -19^th^C | CSM 2056-1 | Sheep | Phalanx | 7.8 | 34.9 | 13.0 | 3.1 | -21.9 | 4.5 | - | - |
|  | 17^th^ -19^th^C | CSM 2061 | Cattle | Rib |  | 45.1 | 16.7 | 3.2 | -20.5 | 6.0 | -13.8 | -4.42 |
|  | 17^th^ -19^th^C | CSM 2170 | Sheep | Phalanx | 22.5 | 42.4 | 15.6 | 3.2 | -21.7 | 5.1 | - | - |
|  | 17^th^ -19^th^C | CSM 3006 | Pig | Tibia | 14.8 | 37.9 | 14.0 | 3.2 | -22.1 | 6.5 | -16.0 | -5.18 |
|  | 17^th^ -19^th^C | CSM 3006-1 | Cattle | Scapula | 7.4 | 35.9 | 12.8 | 3.3 | -21.1 | 6.0 | - | - |
|  | 17^th^ -19^th^C | CSM 3006-2 | Cattle | Tibia | 17.6 | 38.0 | 14.2 | 3.1 | -22.3 | 6.7 | - | - |
|  | 17^th^ -19^th^C | CSM 3007 | Sheep | Metacarpal | 11.9 | 35.5 | 13.0 | 3.2 | -22.4 | 6.4 | - | - |
|  | 17^th^ -19^th^C | CSM 3008 | Sheep | Radius | 4.4 | 21.7 | 8.2 | 3.1 | -22.4 | 5.3 | - | - |
|  | 17^th^ -19^th^C | CSM 3008-1 | Pig | Tibia | 10.5 | 36.9 | 13.7 | 3.2 | -21.3 | 8.8 | -13.4 | -4.76 |
|  | 17^th^ -19^th^C | CSM 3009-1 | Cattle | Tibia | 3.7 | 35.7 | 13.3 | 3.1 | -22.4 | 7.5 | - | - |
|  | 17^th^ -19^th^C | CSM 3009-2 | Cattle | Tibia | 6.1 | 32.9 | 12.2 | 3.2 | -22.2 | 6.5 | - | - |
|  | 17^th^ -19^th^C | CSM 3011 | Sheep | Tibia | 24.1 | 39.6 | 14.6 | 3.2 | -21.4 | 5.0 | - | - |
|  | 17^th^ -19^th^C | CSM 3011-1 | Sheep | Metacarpal | 17.1 | 39.7 | 14.7 | 3.2 | -21.7 | 3.3 | - | - |
|  | 17^th^ -19^th^C | CSM 3011-2 | Sheep | Tibia | 13.8 | 41.1 | 15.2 | 3.2 | -21.4 | 4.5 | - | - |
|  | 17^th^ -19^th^C | CSM 4021 -3 | Sheep | Radius | 25.3 | 40.2 | 14.8 | 3.2 | -22.2 | 5.9 | - | - |
|  | 17^th^ -19^th^C | CSM 4021-1 | Cattle | Tibia |  | 41.8 | 15.3 | 3.2 | -21.9 | 6.1 | - | - |
|  | 17^th^ -19^th^C | CSM 4050 | Cattle | Metatarsal |  | 43.0 | 15.9 | 3.2 | -22.0 | 5.8 | -14.0 | -4.85 |
|  | 17^th^ -19^th^C | CSM 4054 | Cattle | Scapula | 17.8 | 39.3 | 14.5 | 3.2 | -22.1 | 7.0 | -13.8 | -4.95 |
|  | 17^th^ -19^th^C | CSM 4055 | Pig | Tibia | 14.9 | 39.5 | 14.4 | 3.2 | -21.9 | 5.5 | -15.3 | -4.46 |
|  | 17^th^ -19^th^C | CSM 4062 | Cattle | Femur |  | 43.1 | 16.0 | 3.1 | -21.5 | 6.4 | -12.6 | -4.33 |
|  | 17^th^ -19^th^C | CSM 4238 | Sheep | Radius | 7.7 | 40.0 | 14.2 | 3.3 | -21.7 | 6.1 | -14.7 | -5.23 |
|  | 17^th^ -19^th^C | CSM 5027 | Sheep | Femur | 8.8 | 36.1 | 13.2 | 3.2 | -22.4 | 6.2 | - | - |
|  | 17^th^ -19^th^C | CSM 5028 | Mallard | Rib | 6.8 | 33.1 | 11.9 | 3.2 | -19.8 | 11.7 | - | - |
|  | 17^th^ -19^th^C | CSM 5029 | Cattle | Radius | 18.8 | 41.2 | 15.3 | 3.2 | -22.5 | 5.7 | - | - |
|  | 17^th^ -19^th^C | CSM 5030 | Pig | Radius | 14.8 | 39.4 | 14.5 | 3.2 | -21.8 | 6.1 | -15.9 | -5.99 |
|  | 17^th^ -19^th^C | CSM 6134 | Pig | Metapodial | 11.9 | 38.8 | 14.4 | 3.1 | -22.3 | 6.0 | -13.7 | -4.10 |
|  | 17^th^ -19^th^C | CSM 6217 | Pig | Tibia | 16.6 | 43.7 | 16.2 | 3.1 | -16.2 | 6.4 | - | - |
|  | 17^th^ -19^th^C | CSM 6217-1 | Sheep | Metapodial | 10.4 | 39.5 | 14.6 | 3.2 | -22.2 | 4.5 | - | - |
|  | 17^th^ -19^th^C | CSM 6323 | Pig | Tibia | 11.8 | 35.7 | 13.2 | 3.2 | -21.9 | 6.6 | -15.5 | -5.01 |
|  | 17^th^ -19^th^C | CSM 6602 | Cattle | Tibia |  | 43.8 | 15.8 | 3.2 | -22.1 | 5.4 | - | - |
|  | 17^th^ -19^th^C | CSM 6832 | Cattle | Tibia |  | 43.2 | 15.9 | 3.2 | -22.1 | 5.8 | - | - |
|  | 17^th^ -19^th^C | CSM 6838 | Pig | Tibia |  | 43.0 | 15.5 | 3.2 | -20.2 | 8.8 | - | - |
|  | 17^th^ -19^th^C | CSM 6925 | Sheep | Tibia |  | 44.4 | 16.2 | 3.2 | -22.1 | 6.8 | - | - |
| Norton Priory, Chester  (Nothern) | 16^th^-18^th^C | NPBT 1 | Cattle | Tibia | 18.1 | 39.7 | 14.5 | 3.2 | -22.4 | 5.2 | - | - |
|  | 16^th^-18^th^C | NPBT 2 | Cattle | Tibia | 12.0 | 40.0 | 14.5 | 3.2 | -22.2 | 7.1 | - | - |
|  | 16^th^-18^th^C | NPBT 3 | Cattle | Tibia | 14.8 | 42.3 | 15.5 | 3.2 | -22.4 | 5.9 | - | - |
|  | 16^th^-18^th^C | NPBT 4 | Cattle | Tibia | 14.1 | 37.8 | 13.7 | 3.2 | -22.2 | 5.9 | - | - |
|  | 18^th^-20^th^C | NPBT 5 | Cattle | Mandible | 16.8 | 37.6 | 13.9 | 3.2 | -22.1 | 5.8 | - | - |
|  | 18^th^-20^th^C | NPBT 6 | Cattle | Metatarsal | 14.1 | 38.9 | 13.9 | 3.3 | -22.2 | 6.0 | - | - |
|  | 16^th^-18^th^C | NPBT 7 | Cattle | Humerus | 6.9 | 36.2 | 13.3 | 3.2 | -21.9 | 4.9 | - | - |
|  | 16^th^-18^th^C | NPBT 8 | Cattle | Radius | 10.1 | 39.3 | 14.4 | 3.2 | -22.1 | 5.8 | - | - |
|  | 16^th^-18^th^C | NPBT 9 | Cattle | Radius | 10.0 | 40.7 | 14.9 | 3.2 | -22.1 | 6.9 | - | - |
|  | 16^th^-18^th^C | NPBT 10 | Cattle | Radius | 14.4 | 40.8 | 14.8 | 3.2 | -22.2 | 7.2 | - | - |
|  | 16^th^-18^th^C | NPBT 11 | Cattle | Radius | 9.4 | 41.0 | 15.1 | 3.2 | -22.4 | 6.1 | - |  |
|  | 16^th^-18^th^C | NPBT 12 | Cattle | Astragalus | 9.5 | 38.7 | 13.4 | 3.4 | -22.8 | 7.1 | - | - |
|  | 16^th^-18^th^C | NPBT 13 | Cattle | Astragalus | 7.5 | 41.2 | 15.0 | 3.2 | -22.4 | 5.4 | - | - |
|  | 18^th^-20^th^C | NPBT 14 | Cattle | Metatarsal | 4.3 | 39.5 | 14.4 | 3.2 | -22.1 | 6.1 | - | - |
|  | 18^th^-20^th^C | NPBT 15 | Cattle | Metatarsal | 9.0 | 39.9 | 14.7 | 3.2 | -22.1 | 7.0 | - | - |
|  | 18^th^-20^th^C | NPBT 16 | Cattle | Metatarsal | 11.3 | 40.9 | 14.9 | 3.2 | -22.2 | 7.3 | - | - |
|  | 18^th^-20^th^C | NPBT 17 | Cattle | Tibia | 8.3 | 39.6 | 14.6 | 3.2 | -22.4 | 6.1 | - | - |
|  | 18^th^-20^th^C | NPBT 18 | Cattle | Humerus | 5.0 | 36.8 | 13.5 | 3.2 | -21.8 | 5.0 | - | - |
|  | 16^th^-18^th^C | NPBT 19 | Cattle | Femur | 16.0 | 38.3 | 14.1 | 3.2 | -21.5 | 7.3 | - | - |
|  | 16^th^-18^th^C | NPBT 20 | Cattle | Tibia | 10.0 | 40.1 | 14.6 | 3.2 | -22.1 | 6.3 | - | - |
|  | 16^th^-18^th^C | NPDF 1 | Chicken | Metatarsus | 13.7 | 42.4 | 15.4 | 3.2 | -20.5 | 8.1 | - | - |
|  | 16^th^-18^th^C | NPDF 2 | Chicken | Metatarsus | 17.9 | 43.5 | 15.8 | 3.2 | -20.9 | 10.2 | - | - |
|  | 16^th^-18^th^C | NPDF 3 | Chicken | Metatarsus | 17.5 | 46.5 | 16.8 | 3.2 | -20.6 | 8.4 | - | - |
|  | 16^th^-18^th^C | NPDF 4 | Chicken | Metatarsus | 14.4 | 45.9 | 16.7 | 3.2 | -20.8 | 8.3 | - | - |
|  | 16^th^-18^th^C | NPDF 5 | Chicken | Metatarsus | 18.6 | 42.1 | 15.2 | 3.2 | -20.7 | 9.1 | - | - |
|  | 16^th^-18^th^C | NPDF 6 | Chicken | Humerus | 13.3 | 39.8 | 13.9 | 3.4 | -21.0 | 9.6 | - | - |
|  | 16^th^-18^th^C | NPDF 7 | Chicken | Humerus | 13.6 | 41.2 | 15.0 | 3.2 | -21.4 | 9.0 | - | - |
|  | 16^th^-18^th^C | NPDF 8 | Chicken | Femur | 17.2 | 37.6 | 13.7 | 3.2 | -21.2 | 10.1 | - | - |
|  | 16^th^-18^th^C | NPDF 9 | Chicken | Tibiotarsus | 15.4 | 43.1 | 15.5 | 3.2 | -21.8 | 8.4 | - | - |
|  | 16^th^-18^th^C | NPDF 10 | Chicken | Femur | 20.4 | 41.2 | 15.0 | 3.2 | -21.1 | 8.7 | - | - |
|  | 16^th^-18^th^C | NPDF 11 | Chicken | Tarsometatarsus | 14.9 | 42.7 | 15.2 | 3.3 | -20.5 | 11.6 | - | - |
|  | 16^th^-18^th^C | NPDF 12 | Chicken | Tarsometatarsus | 18.9 | 44.2 | 15.9 | 3.2 | -21.4 | 10.9 | - | - |
|  | 16^th^-18^th^C | NPSG 1 | Sheep | Radius | 15.7 | 41.9 | 15.4 | 3.2 | -22.3 | 7.8 | - | - |
|  | 16^th^-18^th^C | NPSG 2 | Sheep | Scapula | 15.6 | 42.5 | 15.6 | 3.2 | -22.2 | 9.0 | - | - |
|  | 16^th^-18^th^C | NPSG 3 | Sheep | Humerus | 15.6 | 42.9 | 15.7 | 3.2 | -21.8 | 9.2 | - | - |
|  | 16^th^-18^th^C | NPSG 4 | Sheep | Humerus | 14.5 | 43.5 | 15.9 | 3.2 | -22.7 | 9.8 | - | - |
|  | 16^th^-18^th^C | NPSG 5 | Sheep | Tibia | 15.2 | 39.1 | 14.5 | 3.1 | -22.2 | 9.3 | - | - |
|  | 16^th^-18^th^C | NPSG 5B | Sheep | Humerus | 16.2 | 40.2 | 14.8 | 3.2 | -21.3 | 7.3 | - | - |
|  | 18^th^-20^th^C | NPSG 6 | Sheep | Scapula | 6.6 | 37.9 | 14.0 | 3.2 | -22.2 | 7.3 | - | - |
|  | 18^th^-20^th^C | NPSG 7 | Sheep | Astragalus | 6.8 | 42.2 | 15.4 | 3.2 | -22.3 | 8.0 | - | - |
|  | 18^th^-20^th^C | NPSG 8 | Sheep | Metacarpal | 9.9 | 40.5 | 14.6 | 3.2 | -22.0 | 7.4 | - | - |
|  | 18^th^-20^th^C | NPSG 9 | Sheep | Tibia | 13.5 | 43.9 | 15.8 | 3.2 | -22.6 | 8.6 | - | - |
|  | 18^th^-20^th^C | NPSG 10 | Sheep | Metatarsal | 18.6 | 43.6 | 16.3 | 3.1 | -21.8 | 8.1 | - | - |
|  | 18^th^-20^th^C | NPSG 11 | Sheep | Tibia | 18.0 | 44.0 | 16.1 | 3.2 | -22.2 | 8.5 | - | - |
|  | 18^th^-20^th^C | NPSG 12 | Sheep | Scapula | 18.0 | 44.0 | 16.3 | 3.2 | -21.7 | 4.6 | - | - |
|  | 18^th^-20^th^C | NPSG 13 | Sheep | Tibia | 13.3 | 40.3 | 14.6 | 3.2 | -22.2 | 6.0 | - | - |
|  | 16^th^-18^th^C | NPSG 14 | Sheep | Tibia | 9.9 | 41.6 | 15.1 | 3.2 | -22.1 | 6.7 | - | - |
|  | 18^th^-20^th^C | NPSG 14B | Sheep | Ulna | 14.3 | 37.5 | 13.9 | 3.2 | -22.2 | 7.2 | - | - |
|  | 16^th^-18^th^C | NPSG 15 | Sheep | Ulna | 16.3 | 40.6 | 14.9 | 3.2 | -22.3 | 5.9 | - | - |
|  | 16^th^-18^th^C | NPSG 16 | Sheep | Humerus | 15.0 | 40.7 | 14.7 | 3.2 | -22.0 | 7.2 | - | - |
|  | 16^th^-18^th^C | NPSG 17 | Sheep | Scapula | 15.8 | 42.4 | 15.5 | 3.2 | -22.5 | 8.9 | - | - |
|  | 16^th^-18^th^C | NPSG 18 | Sheep | Scapula | 15.4 | 42.1 | 15.5 | 3.2 | -22.3 | 10.5 | - | - |
|  | 16^th^-18^th^C | NPSG 19 | Sheep | Humerus | 19.2 | 42.0 | 15.2 | 3.2 | -22.4 | 9.1 | - | - |
|  | 16^th^-18^th^C | NPSG 20 | Sheep | Humerus | 8.8 | 40.2 | 14.6 | 3.2 | -22.0 | 7.2 | - | - |
|  | 16^th^-18^th^C | NPSG 21 | Sheep | Humerus | 4.8 | 41.5 | 15.2 | 3.2 | -21.3 | 7.2 | - | - |
|  | 16^th^-18^th^C | NPSS 1 | Pig | Humerus | 4.9 | 38.1 | 13.9 | 3.2 | -21.3 | 9.3 | - | - |
|  | 16^th^-18^th^C | NPSS 2 | Pig | Metapodial | 11.0 | 37.4 | 13.6 | 3.2 | -21.3 | 9.1 | - | - |
|  | 18^th^-20^th^C | NPSS 3 | Pig | Mandible | 7.1 | 43.6 | 16.0 | 3.2 | -21.2 | 8.4 | - | - |
|  | 18^th^-20^th^C | NPSS 4 | Pig | Mandible | 9.5 | 40.5 | 14.6 | 3.2 | -21.3 | 8.5 | - | - |
|  | 16^th^-18^th^C | NPSS 5 | Pig | Tibia | 5.0 | 43.1 | 15.8 | 3.2 | -21.3 | 8.5 | - | - |
|  | 16^th^-18^th^C | NPSS 6 | Pig | Scapula | 16.8 | 43.4 | 15.6 | 3.2 | -21.9 | 5.1 | - | - |
|  | 16^th^-18^th^C | NPSS 7 | Pig | Humerus | 18.8 | 34.0 | 12.5 | 3.2 | -21.5 | 6.8 | - | - |
|  | 16^th^-18^th^C | NPSS 8 | Pig | Radius | 23.5 | 39.0 | 14.4 | 3.2 | -21.3 | 6.4 | - | - |
|  | 16^th^-18^th^C | NPSS 9 | Pig | Radius | 14.0 | 37.5 | 13.8 | 3.2 | -21.9 | 9.1 | - | - |
|  | 16^th^-18^th^C | NPSS 10 | Pig | Metapodial | 19.7 | 40.9 | 15.2 | 3.1 | -21.2 | 8.7 | -15.6 | -6.00 |
|  | 16^th^-18^th^C | NPSS 11 | Pig | Scapula | 12.4 | 40.3 | 14.8 | 3.2 | -21.5 | 9.0 | -14.7 | -5.06 |
|  | 16^th^-18^th^C | NPSS 12 | Pig | Mandible | 10.6 | 39.6 | 14.1 | 3.3 | -21.3 | 7.2 | - | - |
|  | 18^th^-20^th^C | NPSS 13 | Pig | Ulna | 18.4 | 43.5 | 16.0 | 3.2 | -21.8 | 6.7 | - | - |
|  | 18^th^-20^th^C | NPSS 14 | Pig | Metapodial | 7.3 | 39.8 | 14.3 | 3.2 | -22.1 | 8.1 | -15.8 | -5.52 |
|  | 18^th^-20^th^C | NPSS 15 | Pig | Tibia | 12.2 | 39.9 | 14.6 | 3.2 | -21.9 | 6.9 | -15.9 | -5.60 |
|  | 18^th^-20^th^C | NPSS 16 | Pig | Maxilla | 9.4 | 40.9 | 14.9 | 3.2 | -21.1 | 9.5 | -14.3 | -5.36 |
|  | 18^th^-20^th^C | NPSS 17 | Pig | Radius | 17.7 | 38.1 | 13.9 | 3.2 | -21.1 | 6.6 | -14.4 | -4.73 |
|  | 18^th^-20^th^C | NPSS 18 | Pig | Metapodial | 13.9 | 35.9 | 13.2 | 3.2 | -20.8 | 7.1 | - | - |
|  | 18^th^-20^th^C | NPSS 19 | Pig | Tibia | 9.2 | 41.5 | 15.0 | 3.2 | -21.9 | 5.2 | - | - |
|  | 18^th^-20^th^C | NPSS 20 | Pig | Ulna | 19.9 | 37.4 | 13.6 | 3.2 | -21.2 | 6.5 | - | - |
| Hungate, York  (Northern) | 19^th^ C | HUN 01 | Sheep | Mandible | - | 39.8 | 14.3 | 3.2 | -22.3 | 7.2 | - | - |
|  | 19^th^ C | HUN 02 | Sheep | Mandible | - | 43.3 | 15.9 | 3.1 | -21.9 | 4.5 | - | - |
|  | 19^th^ C | HUN 03 | Sheep | Mandible | - | 41.0 | 15.3 | 3.1 | -22.1 | 9.2 | - | - |
|  | 15^th^ C | HUN 027 | Sheep | Metacarpal | - | 39.3 | 14.3 | 3.2 | -22.6 | 6.2 | - | - |
|  | 18^th^-19^th^ C | HUN 064 | Sheep | Metacarpal | - | 39.3 | 14.1 | 3.2 | -21.6 | 4.6 | - | - |
|  | 18^th^-19^th^ C | HUN 080 | Sheep | Metacarpal | - | 39.2 | 14.2 | 3.2 | -22.1 | 6.1 | - | - |
|  | 18^th^-19^th^ C | HUN 082 | Sheep | Metacarpal | - | 40.0 | 14.4 | 3.2 | -22.2 | 6.5 | - | - |
|  | 20^th^ C | HUN 101 | Sheep | Metacarpal | - | 39.2 | 14.3 | 3.2 | -22.2 | 4.1 | - | - |
|  | 20^th^ C | HUN 102 | Sheep | Metacarpal | - | 39.3 | 14.2 | 3.2 | -22.3 | 5.3 | - | - |
|  | 20^th^ C | HUN 105 | Sheep | Metacarpal | - | 38.8 | 14.3 | 3.2 | -22.2 | 7.0 | - | - |
|  | 20^th^ C | HUN 108 | Sheep | Metacarpal | - | 40.0 | 14.5 | 3.2 | -23.0 | 7.0 | - | - |
|  | 19^th^ C | HUN 160 | Sheep | Metacarpal | - | 39.0 | 14.0 | 3.2 | -22.1 | 4.7 | - | - |
|  | 19^th^ C | HUN 161 | Sheep | Metacarpal | - | 38.4 | 13.9 | 3.2 | -22.5 | 6.7 | - | - |
|  | 18^th^-19^th^ C | HUN 162 | Sheep | Metacarpal | - | 43.0 | 15.5 | 3.2 | -22.3 | 6.1 | - | - |
|  | 18^th^-19^th^ C | HUN 163 | Sheep | Metacarpal | - | 36.2 | 13.2 | 3.2 | -22.0 | 8.9 | - | - |
|  | 18^th^-19^th^ C | HUN 165 | Sheep | Metacarpal | - | 43.0 | 15.6 | 3.2 | -22.7 | 8.2 | - | - |
|  | 16^th^-17^th^ C | HUN 241 | Sheep | Metatarsal | - | 44.5 | 16.1 | 3.2 | -22.5 | 6.4 | - | - |
|  | 16^th^-17^th^ C | HUN 267 | Sheep | Metatarsal | - | 43.1 | 15.6 | 3.2 | -21.5 | 6.1 | - | - |
|  | 18^th^-19^th^ C | HUN 422 | Sheep | Metacarpal | - | 42.7 | 15.6 | 3.2 | -21.8 | 3.6 | - | - |
|  | 18^th^-19^th^ C | HUN 426 | Sheep | Metatarsal | - | 39.6 | 14.5 | 3.2 | -21.7 | 6.9 | - | - |
|  | 18^th^-19^th^ C | HUN 331 | Sheep | Metacarpal | - | 38.9 | 14.3 | 3.2 | -22.1 | 3.9 | - | - |
| The Bedern, York  (Northern) | 15^th^ C | BED 01 | Sheep | Metacarpal | - | 37.6 | 13.9 | 3.2 | -22.1 | 3.9 | - | - |
|  | 15^th^ C | BED 02 | Sheep | Pelvis | - | 40.8 | 15.2 | 3.1 | -22.3 | 8.8 | - | - |
|  | 15^th^ C | BED 03 | Sheep | Metacarpal | - | 35.8 | 13.5 | 3.1 | -21.8 | 5.5 | - | - |
|  | 15^th^ C | BED 04 | Sheep | Scapula | - | 38.7 | 14.2 | 3.2 | -22.3 | 9.0 | - | - |
|  | 15^th^ C | BED 05 | Sheep | Metacarpal | - | 35.7 | 13.6 | 3.1 | -22.1 | 5.0 | - | - |
|  | 18^th^-19^th^ C | BED 07 | Sheep | Tibia | - | 38.5 | 14.5 | 3.1 | -21.5 | 5.6 | - | - |
|  | 18^th^-19^th^ C | BED 08 | Sheep | Humerus | - | 37.2 | 14.2 | 3.1 | -21.8 | 5.4 | - | - |
|  | 18^th^-19^th^ C | BED 09 | Sheep | Tibia | - | 38.4 | 14.5 | 3.1 | -21.5 | 4.8 | - | - |
|  | 18^th^-19^th^ C | BED 15 | Sheep | Metacarpal | - | 37.9 | 14.3 | 3.1 | -20.8 | 8.7 | - | - |
|  | 18^th^-19^th^ C | BED 16 | Sheep | Metacarpal | - | 36.2 | 13.7 | 3.1 | -22.2 | 8.0 | - | - |
|  | 18^th^-19^th^ C | BED 18 | Sheep | Metacarpal | - | 34.5 | 12.9 | 3.1 | -22.3 | 5.2 | - | - |
| Walmgate, York  (Northern) | 18^th^-19^th^ C | WAL 01 | Sheep | Mandible | - | 43.0 | 5.5 | 3.2 | -20.8 | 6.3 | - | - |
|  | 18^th^-19^th^ C | WAL 02 | Sheep | Mandible | - | 39.1 | 13.3 | 3.2 | -21.7 | 4.4 | - | - |
|  | 18^th^-19^th^ C | WAL 03 | Sheep | Mandible | - | 42.8 | 15.6 | 3.2 | -21.7 | 5.4 | - | - |
| Oulton, Leeds  (Northern) | 18^th^-19^th^ C | Flee (061)(062) | Cattle | Metacarpal | 14.4 | 36.8 | 13.5 | 3.2 | -21.7 | 7.9 | - | - |
|  | 18^th^-19^th^ C | Flee 053 | Cattle | Metacarpal | 10.3 | 33.3 | 12.2 | 3.2 | -23.3 | 9.1 | - | - |
|  | 18^th^-19^th^ C | Flee 053-1 | Cattle | Tibia | 12.5 | 39.5 | 14.5 | 3.2 | -22.5 | 8.3 | - | - |
|  | 18^th^-19^th^ C | Flee 053-3 | Cattle | Metacarpal | 11.8 | 34.0 | 12.7 | 3.1 | -22.7 | 8.5 | - | - |
| Otley, Leeds  (Northern) | 18^th^-19^th^ C | GPMO 037 | Cattle | Long bone | 19.3 | 40.0 | 14.7 | 3.2 | -22.1 | 4.4 | -13.0 | -5.68 |
|  | 18^th^-19^th^ C | GPMO 159 | Pig | Femur | 21.5 | 39.8 | 14.6 | 3.2 | -11.5 | 9.3 | -9.9 | -4.97 |
|  | 18^th^-19^th^ C | GPMO 168 | Sheep | Metapodial | 21.0 | 37.4 | 13.7 | 3.2 | -21.7 | 5.5 | - | - |
|  | 18^th^-19^th^ C | GPMO 169 | Sheep | Radius | 9.5 | 39.6 | 14.5 | 3.2 | -22.3 | 6.7 | - | - |
|  | 18^th^-19^th^ C | GPMO UC1 | Sheep | Radius | 15.1 | 40.4 | 14.7 | 3.2 | -22.4 | 6.5 | - | - |
|  | 18^th^-19^th^ C | GPMO UC1-2 | Cattle | Rib | 22.9 | 42.1 | 15.3 | 3.2 | -22.2 | 7.3 | - | - |
|  | 18^th^-19^th^ C | GPMO UC2 | Cattle | Femur | 20.4 | 40.4 | 14.9 | 3.2 | -22.7 | 7.1 | - | - |
|  | 18^th^-19^th^ C | GPMO UC3 | Pig | Tibia | 17.9 | 40.1 | 14.7 | 3.2 | -8.6 | 6.6 | -10.3 | -3.89 |
| Sand-le-Mere  (Northern) | 16^th^ - 19^th^ C | SALM T8 801 | Cattle | Pelvis | 15.7 | 39.9 | 14.6 | 3.2 | -21.7 | 6.7 | - | - |
|  | 16^th^ - 19^th^ C | SALM T8 803 | Cattle | Maxilla | 4.8 | 35.7 | 13.0 | 3.2 | -21.3 | 8.1 | - | - |
|  | 16^th^ - 19^th^ C | SALM T8 807 | Cattle | Ulna | 11.8 | 21.1 | 7.8 | 3.2 | -22.2 | 4.9 | - | - |
| Square Chapel Halifax  (Northern) | 18^th^ - 19^th^C | SQC 1006 | Sheep | Femur | 12.3 | 37.9 | 13.7 | 3.2 | -20.5 | 10.2 | -13.2 | -4.49 |
|  | 18^th^ - 19^th^C | SQC 1012-1 | Cattle | Long bone | 23.6 | 42.5 | 15.5 | 3.2 | -21.7 | 8.1 | -6.8 | -6.33 |
|  | 18^th^ - 19^th^C | SQC 1012-2 | Pig | Tibia | 24.7 | 40.6 | 14.9 | 3.2 | -22.3 | 10.1 | -12.5 | -4.71 |
|  | 18^th^ - 19^th^C | SQC 1012-3 | Sheep | Tibia | 13.3 | 39.1 | 13.9 | 3.3 | -22.7 | 7.8 | -13.4 | -3.70 |
|  | 18^th^ - 19^th^C | SQC 1012-4 | Sheep | Calcaneus | 9.5 | 42.2 | 15.4 | 3.2 | -21.7 | 8.1 | -12.8 | -3.89 |
|  | 18^th^ - 19^th^C | SQC 1250 | Sheep | Tibia | 17.1 | 37.3 | 13.7 | 3.2 | -22.4 | 6.2 | -15.3 | -6.07 |
|  | 18^th^ - 19^th^C | SQC 1355 | Cattle | Metatarsal | 7.6 | 21.1 | 7.7 | 3.2 | -22.7 | 3.7 | - | - |

**Data**: - = No data available

Table S3: FTIR-ATR results for all post-medieval samples in this study. Each sample was measured in triplicate

| *Sample* | *Human/Animal* | *IRSF (Mean ± σ)* | *C/P (Mean ± σ)* |
| --- | --- | --- | --- |
| CSM 1.07 | Human | 3.42 ± 0.04 | 0.21 ± 0.00 |
| CSM 1.12 | Human | 3.87 ± 0.00 | 0.16 ± 0.00 |
| CSM 1.27 | Human | 3.91 ± 0.03 | 0.16 ± 0.00 |
| CSM 1.37 | Human | 3.50 ± 0.03 | 0.21 ± 0.00 |
| CSM 1.41 | Human | 3.80 ± 0.01 | 0.18 ± 0.00 |
| CSM 2.05 | Human | 3.76 ± 0.02 | 0.14 ± 0.00 |
| CSM 2.07 | Human | 3.68 ± 0.08 | 0.18 ± 0.00 |
| CSM 2.12 | Human | 3.73 ± 0.02 | 0.14 ± 0.00 |
| CSM 2.15 | Human | 3.68 ± 0.06 | 0.18 ± 0.00 |
| CSM 2.16 | Human | 3.80 ± 0.03 | 0.18 ± 0.00 |
| CSM 2.18 | Human | 3.80 ± 0.02 | 0.17 ± 0.00 |
| CSM 2.2 | Human | 3.76 ± 0.02 | 0.15 ± 0.00 |
| CSM 2.21 | Human | 3.75 ± 0.04 | 0.18 ± 0.00 |
| CSM 2.25 | Human | 3.92 ± 0.06 | 0.16 ± 0.00 |
| CSM 2.29 | Human | 3.92 ± 0.06 | 0.17 ± 0.00 |
| CSM 2.3 | Human | 3.74 ± 0.01 | 0.18 ± 0.00 |
| CSM 2.31 | Human | 3.58 ± 0.07 | 0.20 ± 0.00 |
| CSM 2.32 | Human | 3.70 ± 0.11 | 0.18 ± 1.00 |
| CSM 2.34 | Human | 4.05 ± 0.09 | 0.15 ± 0.00 |
| CSM 2.35 | Human | 3.74 ± 0.00 | 0.18 ± 0.00 |
| CSM 2.36 | Human | 4.01 ± 0.08 | 0.14 ± 0.00 |
| CSM 2.37 | Human | 4.01 ± 0.04 | 0.14 ± 0.00 |
| CSM 2.4 | Human | 4.02 ± 0.15 | 0.15 ± 1.00 |
| CSM 2.41 | Human | 3.74 ± 0.00 | 0.18 ± 0.00 |
| CSM 2.43 | Human | 3.94 ± 0.07 | 0.14 ± 0.00 |
| CSM 2.49 | Human | 3.87 ± 0.03 | 0.16 ± 0.00 |
| CSM 2.51 | Human | 3.77 ± 0.03 | 0.15 ± 0.00 |
| CSM 2.52 | Human | 3.44 ± 0.01 | 0.23 ± 0.00 |
| CSM 2.54 | Human | 3.61 ± 0.02 | 0.17 ± 0.00 |
| CSM 3 | Human | 3.87 ± 0.01 | 0.16 ± 0.00 |
| CSM 3.25 | Human | 3.84 ± 0.09 | 0.16 ± 0.00 |
| CSM 3.33 | Human | 4.00 ± 0.07 | 0.14 ± 0.00 |
| CSM 3.34 | Human | 4.01 ± 0.02 | 0.14 ± 0.00 |
| CSM 3.36 | Human | 4.07 ± 0.01 | 0.14 ± 0.00 |
| CSM 3.43 | Human | 3.66 ± 0.01 | 0.15 ± 0.00 |
| CSM 3.45 | Human | 3.51 ± 0.07 | 0.19 ± 0.00 |
| CSM 3.48 | Human | 3.79 ± 0.07 | 0.15 ± 0.00 |
| CSM 3.53 | Human | 3.81 ± 0.02 | 0.16 ± 0.00 |
| CSM 4.05 | Human | 4.08 ± 0.00 | 0.14 ± 0.00 |
| CSM 4.11 | Human | 3.60 ± 0.03 | 0.18 ± 0.00 |
| CSM 4.12 | Human | 3.81 ± 0.02 | 0.16 ± 0.00 |
| CSM 4.24 | Human | 4.02 ± 0.02 | 0.15 ± 0.00 |
| CSM 4.28 | Human | 3.56 ± 0.14 | 0.19 ± 0.00 |
| CSM 4.38 | Human | 3.85 ± 0.06 | 0.17 ± 0.00 |
| CSM 4.53 | Human | 3.88 ± 0.09 | 0.16 ± 0.00 |
| CSM 5.05 | Human | 3.53 ± 0.05 | 0.19 ± 0.00 |
| CSM 5.07 | Human | 3.91 ± 0.05 | 0.14 ± 0.00 |
| CSM 5.09 | Human | 3.60 ± 0.01 | 0.18 ± 0.00 |
| CSM 5.16 | Human | 3.80 ± 0.02 | 0.18 ± 0.00 |
| CSM 5.23 | Human | 3.62 ± 0.04 | 0.17 ± 0.00 |
| CSM 5.36 | Human | 3.72 ± 0.02 | 0.15 ± 0.00 |
| CSM 61.03 | Human | 3.30 ± 0.02 | 0.25 ± 0.00 |
| CSM 61.04 | Human | 3.81 ± 0.04 | 0.18 ± 0.00 |
| CSM 62.02 | Human | 3.86 ± 0.04 | 0.16 ± 0.00 |
| HGM 1 | Human | 3.91 ± 0.03 | 0.13 ± 0.00 |
| HGM 2 | Human | 3.47 ± 0.03 | 0.20 ± 0.00 |
| HGM 3 | Human | 3.77 ± 0.03 | 0.14 ± 0.00 |
| HGM 4 | Human | 4.05 ± 0.03 | 0.11 ± 0.00 |
| HGM 5 | Human | 3.49 ± 0.03 | 0.21 ± 0.00 |
| HGM 6 | Human | 3.82 ± 0.03 | 0.16 ± 0.00 |
| HGM 8 | Human | 3.65 ± 0.03 | 0.15 ± 0.00 |
| HGM 9 | Human | 3.77 ± 0.02 | 0.16 ± 0.00 |
| HGM 10 | Human | 3.71 ± 0.02 | 0.16 ± 0.00 |
| HGM 11 | Human | 4.01 ± 0.02 | 0.12 ± 0.00 |
| HGM 12 | Human | 3.75 ± 0.02 | 0.15 ± 0.00 |
| HGM 13 | Human | 4.00 ± 0.02 | 0.12 ± 0.00 |
| HGM 15 | Human | 3.93 ± 0.01 | 0.13 ± 0.00 |
| HGM 16 | Human | 3.63 ± 0.02 | 0.14 ± 0.00 |
| HGM 17 | Human | 3.97 ± 0.03 | 0.12 ± 0.00 |
| HGM 19 | Human | 3.88 ± 0.03 | 0.13 ± 0.00 |
| HGM 20 | Human | 3.64 ± 0.03 | 0.15 ± 0.00 |
| HGM 21 | Human | 3.54 ± 0.02 | 0.19 ± 0.00 |
| HGM 22 | Human | 3.60 ± 0.02 | 0.16 ± 0.00 |
| HGM 23 | Human | 3.61 ± 0.02 | 0.16 ± 0.00 |
| HGM 24 | Human | 3.69 ± 0.02 | 0.17 ± 0.00 |
| HGM 26 | Human | 3.90 ± 0.02 | 0.13 ± 0.00 |
| HGM 27 | Human | 3.75 ± 0.02 | 0.16 ± 0.00 |
| HGM 28 | Human | 3.84 ± 0.02 | 0.13 ± 0.00 |
| HGM 29 | Human | 3.72 ± 0.04 | 0.16 ± 0.00 |
| HGM 30 | Human | 3.84 ± 0.02 | 0.13 ± 0.00 |
| HGM 31 | Human | 3.77 ± 0.02 | 0.15 ± 0.00 |
| HGM 32 | Human | 4.06 ± 0.03 | 0.11 ± 0.00 |
| HGM 33 | Human | 3.76 ± 0.03 | 0.14 ± 0.00 |
| HGM 34 | Human | 3.90 ± 0.02 | 0.13 ± 0.00 |
| HGM 35 | Human | 3.77 ± 0.01 | 0.13 ± 0.00 |
| HGM 36 | Human | 3.65 ± 0.01 | 0.15 ± 0.00 |
| FEW 53 | Human | 3.72 ± 0.03 | 0.19 ± 0.00 |
| FEW 77 | Human | 3.59 ± 0.02 | 0.19 ± 0.00 |
| FEW 130 | Human | 3.77 ± 0.05 | 0.17 ± 0.00 |
| FEW 156 | Human | 3.52 ± 0.01 | 0.23 ± 0.00 |
| FEW 177 | Human | 3.67 ± 0.09 | 0.17 ± 0.00 |
| FEW 238 | Human | 3.37 ± 0.04 | 0.25 ± 0.00 |
| FEW 241 | Human | 3.58 ± 0.06 | 0.20 ± 0.00 |
| SBK 58 | Human | 3.50 ± 0.01 | 0.21 ± 0.00 |
| SQC 35 | Human | 3.56 ± 0.01 | 0.22 ± 0.00 |
| SQC 39 | Human | 3.51 ± 0.02 | 0.21 ± 0.00 |
| SQC 59 | Human | 3.60 ± 0.03 | 0.20 ± 0.00 |
| SQC 80 | Human | 3.70 ± 0.02 | 0.18 ± 0.00 |
| SQC 88 | Human | 3.72 ± 0.02 | 0.18 ± 0.00 |
| SQC 92 | Human | 3.74 ± 0.03 | 0.16 ± 0.00 |
| SQC 98 | Human | 3.93 ± 0.06 | 0.13 ± 0.00 |
| SQC 119 | Human | 3.64 ± 0.03 | 0.16 ± 0.00 |
| SQC 140 | Human | 3.57 ± 0.02 | 0.18 ± 0.00 |
| SQC 191 | Human | 3.80 ± 0.01 | 0.12 ± 0.00 |
| SQC 1144 | Human | 4.07 ± 0.02 | 0.13 ± 0.00 |
| SQC 1146 | Human | 3.96 ± 0.02 | 0.15 ± 0.00 |
| SQC 1159 | Human | 4.04 ± 0.02 | 0.15 ± 0.00 |
| SQC 1171 | Human | 3.57 ± 0.03 | 0.18 ± 0.00 |
| SQC 1202 | Human | 3.68 ± 0.02 | 0.18 ± 0.00 |
| SQC 1232 | Human | 3.65 ± 0.02 | 0.18 ± 0.00 |
| SQC 1247 | Human | 4.03 ± 0.04 | 0.14 ± 0.00 |
| SQC 1268 | Human | 4.07 ± 0.02 | 0.13 ± 0.00 |
| SQC 1329 | Human | 3.66 ± 0.02 | 0.18 ± 0.00 |
| SQC 1337 | Human | 3.85 ± 0.02 | 0.16 ± 0.00 |
| SQC 1347 | Human | 3.56 ± 0.03 | 0.18 ± 0.00 |
| SQC 1357 | Human | 3.73 ± 0.02 | 0.16 ± 0.00 |
| SQC 1377 | Human | 4.03 ± 0.01 | 0.14 ± 0.00 |
| SQC 1381 | Human | 3.92 ± 0.02 | 0.14 ± 0.00 |
| SQC 1384 | Human | 3.88 ± 0.01 | 0.16 ± 0.00 |
| SQC 1415 | Human | 4.15 ± 0.02 | 0.13 ± 0.00 |
| SQC 1452 | Human | 3.52 ± 0.03 | 0.19 ± 0.00 |
| SQC 1470 | Human | 3.65 ± 0.03 | 0.17 ± 0.00 |
| SQC 1482 | Human | 3.73 ± 0.02 | 0.17 ± 0.00 |
| SQC 1513 | Human | 3.76 ± 0.02 | 0.17 ± 0.00 |
| SQC 1525 | Human | 3.76 ± 0.02 | 0.16 ± 0.00 |
| SQC 1546 | Human | 3.59 ± 0.03 | 0.19 ± 0.00 |
| VGL 2 | Human | 3.72 ± 0.04 | 0.18 ± 0.00 |
| VGL 6 | Human | 3.55 ± 0.02 | 0.20 ± 0.00 |
| VGL 12 | Human | 3.49 ± 0.03 | 0.22 ± 0.00 |
| STGC 1003 | Human | 3.87 ± 0.03 | 0.13 ± 0.00 |
| STGC 1006 | Human | 3.53 ± 0.02 | 0.15 ± 0.00 |
| STGC 1010 | Human | 3.39 ± 0.03 | 0.17 ± 0.00 |
| STGC 1014 | Human | 3.49 ± 0.03 | 0.17 ± 0.00 |
| STGC 1017 | Human | 3.45 ± 0.03 | 0.17 ± 0.00 |
| STGC 1020 | Human | 3.43 ± 0.03 | 0.17 ± 0.00 |
| STGC 1024 | Human | 3.40 ± 0.02 | 0.17 ± 0.00 |
| STGC 1029 | Human | 3.45 ± 0.02 | 0.16 ± 0.00 |
| STGC 5003 | Human | 3.80 ± 0.03 | 0.13 ± 0.00 |
| ROM 03 | Human | 3.75 ± 0.03 | 0.18 ± 0.00 |
| ROM 07 | Human | 3.91 ± 0.04 | 0.15 ± 0.00 |
| ROM 09 | Human | 3.82 ± 0.03 | 0.17 ± 0.00 |
| ROM 11 | Human | 3.84 ± 0.03 | 0.15 ± 0.00 |
| ROM 13 | Human | 3.88 ± 0.01 | 0.15 ± 0.00 |
| ROM 17 | Human | 4.06 ± 0.02 | 0.13 ± 0.00 |
| ROM 23 | Human | 3.48 ± 0.02 | 0.20 ± 0.00 |
| ROM 24 | Human | 3.61 ± 0.05 | 0.15 ± 0.00 |
| ROM 25 | Human | 3.50 ± 0.03 | 0.24 ± 0.00 |
| ROM 28 | Human | 3.72 ± 0.02 | 0.16 ± 0.00 |
| ROM 31 | Human | 3.66 ± 0.03 | 0.20 ± 0.00 |
| ROM 35 | Human | 3.66 ± 0.03 | 0.20 ± 0.00 |
| ROM 37 | Human | 3.86 ± 0.02 | 0.15 ± 0.00 |
| ROM 44 | Human | 3.70 ± 0.01 | 0.18 ± 0.00 |
| ROM 55 | Human | 3.54 ± 0.01 | 0.21 ± 0.00 |
| ROM 56 | Human | 3.43 ± 0.03 | 0.21 ± 0.00 |
| ROM 57 | Human | 3.84 ± 0.01 | 0.16 ± 0.00 |
| ROM 58 | Human | 3.72 ± 0.05 | 0.16 ± 0.00 |
| ROM 60 | Human | 3.90 ± 0.02 | 0.15 ± 0.00 |
| QCS 122 | Human | 3.58 ± 0.05 | 0.16 ± 0.00 |
| QCS 124 | Human | 3.27 ± 0.07 | 0.25 ± 0.00 |
| QCS 163 | Human | 4.09 ± 0.07 | 0.14 ± 0.00 |
| QCS 534 | Human | 3.99 ± 0.07 | 0.16 ± 0.00 |
| QCS 589 | Human | 3.55 ± 0.07 | 0.19 ± 0.00 |
| QCS 1123 | Human | 4.23 ± 0.01 | 0.12 ± 0.00 |
| QCS 1804 | Human | 3.88 ± 0.01 | 0.15 ± 0.00 |
| QCS 1810 | Human | 3.89 ± 0.01 | 0.17 ± 0.00 |
| QCS 1817 | Human | 3.64 ± 0.07 | 0.17 ± 0.00 |
| QCS 1998 | Human | 3.07 ± 0.02 | 0.25 ± 0.00 |
| SBK 7 | Human | 3.75 ± 0.02 | 0.17 ± 0.00 |
| SBK 8 | Human | 3.99 ± 0.03 | 0.13 ± 0.00 |
| SBK 9 | Human | 3.65 ± 0.03 | 0.17 ± 0.00 |
| SBK 10 | Human | 3.68 ± 0.02 | 0.19 ± 0.00 |
| SBK 11 | Human | 3.80 ± 0.02 | 0.16 ± 0.00 |
| SBK 12 | Human | 3.65 ± 0.02 | 0.19 ± 0.00 |
| SBK 15 | Human | 3.60 ± 0.01 | 0.19 ± 0.00 |
| SBK 17 | Human | 3.71 ± 0.01 | 0.17 ± 0.00 |
| SBK 18 | Human | 3.53 ± 0.03 | 0.23 ± 0.00 |
| SBK 21 | Human | 3.69 ± 0.03 | 0.20 ± 0.00 |
| SBK 26 | Human | 3.76 ± 0.01 | 0.17 ± 0.00 |
| SBK 30 | Human | 3.43 ± 0.01 | 0.24 ± 0.00 |
| SBK 34 | Human | 3.64 ± 0.02 | 0.20 ± 0.00 |
| SBK 36 | Human | 3.75 ± 0.02 | 0.15 ± 0.00 |
| SBK 43 | Human | 3.66 ± 0.02 | 0.17 ± 0.00 |
| SBK 44 | Human | 3.65 ± 0.02 | 0.17 ± 0.00 |
| SBK 45 | Human | 3.60 ± 0.02 | 0.20 ± 0.00 |
| SBK 46 | Human | 3.50 ± 0.02 | 0.19 ± 0.00 |
| SBK 48 | Human | 3.47 ± 0.02 | 0.20 ± 0.00 |
| SBK 53 | Human | 3.47 ± 0.02 | 0.23 ± 0.00 |
| SBK 54 | Human | 3.48 ± 0.02 | 0.24 ± 0.00 |
| SBK 57 | Human | 3.56 ± 0.02 | 0.21 ± 0.00 |
| SBK 58 | Human | 3.50 ± 0.01 | 0.21 ± 0.00 |
| RLH 103 | Human | 3.77 ± 0.01 | 0.17 ± 0.00 |
| RLH 135 | Human | 3.44 ± 0.01 | 0.24 ± 0.00 |
| RLH 208 | Human | 3.66 ± 0.02 | 0.20 ± 0.00 |
| RLH 340 | Human | 3.76 ± 0.02 | 0.14 ± 0.00 |
| RLH 349 | Human | 3.65 ± 0.02 | 0.17 ± 0.00 |
| RLH 356 | Human | 3.64 ± 0.02 | 0.17 ± 0.00 |
| RLH 367 | Human | 3.59 ± 0.02 | 0.19 ± 0.00 |
| RLH 386 | Human | 3.51 ± 0.02 | 0.19 ± 0.00 |
| RLH 397 | Human | 3.47 ± 0.02 | 0.20 ± 0.00 |
| RLH 421 | Human | 3.52 ± 0.02 | 0.24 ± 0.00 |
| RLH 572 | Human | 3.48 ± 0.02 | 0.24 ± 0.00 |
| SBL 1203 | Human | 3.57 ± 0.03 | 0.21 ± 0.00 |
| SBL 1207 | Human | 3.50 ± 0.03 | 0.21 ± 0.00 |
| SBL 1215 | Human | 3.76 ± 0.03 | 0.17 ± 0.00 |
| SBL 1244.1 | Human | 4.02 ± 0.03 | 0.13 ± 0.00 |
| SBL 1526 | Human | 3.65 ± 0.02 | 0.19 ± 0.00 |
| SBL 1558 | Human | 3.67 ± 0.03 | 0.18 ± 0.00 |
| SBL 1641 | Human | 3.67 ± 0.04 | 0.16 ± 0.00 |
| SBL 1653 | Human | 3.64 ± 0.04 | 0.19 ± 0.00 |
| SBL 1785 | Human | 3.59 ± 0.04 | 0.19 ± 0.00 |
| SBL 1799 | Human | 3.73 ± 0.04 | 0.17 ± 0.00 |
| SBL 1872 | Human | 3.52 ± 0.04 | 0.24 ± 0.00 |
| SBL 1932 | Human | 3.68 ± 0.03 | 0.19 ± 0.00 |
| SBL 2049 | Human | 3.60 ± 0.04 | 0.19 ± 0.00 |
| SBL 2134 | Human | 3.71 ± 0.04 | 0.17 ± 0.00 |
| SBL 2296 | Human | 3.61 ± 0.04 | 0.19 ± 0.00 |
| CSM 2056 | Animal | 3.77 ± 0.03 | 0.14 ± 0.00 |
| CSM 2061 | Animal | 3.68 ± 0.03 | 0.17 ± 0.00 |
| CSM 3006 | Animal | 3.66 ± 0.02 | 0.18 ± 0.00 |
| CSM 3008 | Animal | 3.76 ± 0.02 | 0.17 ± 0.00 |
| CSM 4050 | Animal | 3.87 ± 0.04 | 0.13 ± 0.00 |
| CSM 4054 | Animal | 3.68 ± 0.02 | 0.16 ± 0.00 |
| CSM 4055 | Animal | 3.70 ± 0.02 | 0.16 ± 0.00 |
| CSM 4062 | Animal | 3.52 ± 0.02 | 0.18 ± 0.00 |
| CSM 4238 | Animal | 3.69 ± 0.01 | 0.17 ± 0.00 |
| CSM 5030 | Animal | 3.97 ± 0.03 | 0.12 ± 0.00 |
| CSM 6134 | Animal | 3.70 ± 0.03 | 0.16 ± 0.00 |
| CSM 6323 | Animal | 3.73 ± 0.03 | 0.15 ± 0.00 |
| GPMO 037- | Animal | 3.54 ± 0.01 | 0.19 ± 0.00 |
| GPMO 159- | Animal | 3.50 ± 0.02 | 0.19 ± 0.00 |
| GPMO U/SC 3 | Animal | 3.81 ± 0.05 | 0.14 ± 0.00 |
| NPSS 10 | Animal | 3.69 ± 0.02 | 0.17 ± 0.00 |
| NPSS 11 | Animal | 3.88 ± 0.01 | 0.13 ± 0.00 |
| NPSS 14 | Animal | 3.69 ± 0.02 | 0.16 ± 0.00 |
| NPSS 15 | Animal | 3.53 ± 0.03 | 0.18 ± 0.00 |
| NPSS 16 | Animal | 3.70 ± 0.03 | 0.17 ± 0.00 |
| NPSS 17 | Animal | 3.71 ± 0.02 | 0.15 ± 0.00 |
| SQC 1012-1 | Animal | 3.70 ± 0.02 | 0.15 ± 0.00 |
| SQC 1012-2 | Animal | 3.87 ± 0.02 | 0.15 ± 0.00 |
| SQC 1012-3 | Animal | 3.66 ± 0.01 | 0.18 ± 0.00 |
| SQC 1012-4 | Animal | 3.75 ± 0.01 | 0.17 ± 0.00 |
| SQC 1012 | Animal | 3.69 ± 0.03 | 0.17 ± 0.00 |
| SQC 1250 | Animal | 3.69 ± 0.03 | 0.16 ± 0.00 |

Table S4: Independent samples Mann-Whitney U Tests (significance set to 0.05) between males and females in sites where there was a sufficient number of sexed individuals to compare. F? And M? were assumed to be F and M respectively

| *Site* | *N* | *δ^13^Ccollagen* | *δ^13^Ccarbonate* | *δ^15^Ncollagen* |
| --- | --- | --- | --- | --- |
| *CSM* | *F=24; M=21* | U=209.5; p = 0.335 | U=248.5; p = 0.941 | U=237.0; p = 0.745 |
| *SCH* | *F=10; M=16* | U=70.0; p = 0.612 | U=52.0; p = 0.145 | U=75.0; p = 0.806 |
| *ROM* | *F=10; M=10* | U=47.5; p = 0.867 | U=44.5; p = 0.698 | U=38.0; p = 0.382 |
| *SBK* | *F=10; M=13* | U=45.5; p = 0.235 | U=51.0; p = 0.400 | U=46.0; p = 0.249 |
| *SBL* | *F=8; M=7* | U=22.0; p = 0.512 | U=18.5; p = 0.291 | U=21.0; p = 0.444 |

**Table S5:** Tests of normality for all isotope data

| Isotopes | Sites | Kolmogorov-Smirnov^a^ | | | Shapiro-Wilk | | |
| --- | --- | --- | --- | --- | --- | --- | --- |
|  |  | Statistic | df | Sig. | Statistic | df | Sig. |
| δ^13^C_coll_ (‰) | CSM | .086 | 54 | .200^*^ | .979 | 54 | .446 |
|  | HGM | .127 | 31 | .200^*^ | .934 | 31 | .057 |
|  | FEW | .203 | 7 | .200^*^ | .940 | 7 | .638 |
|  | SCH | .104 | 32 | .200^*^ | .976 | 32 | .691 |
|  | SGC | .238 | 9 | .149 | .838 | 9 | .055 |
|  | VGL | .253 | 3 | . | .964 | 3 | .637 |
|  | ROM | .115 | 21 | .200^*^ | .977 | 21 | .872 |
|  | QCS | .466 | 10 | .000 | .449 | 10 | .000 |
|  | SBK | .162 | 23 | .122 | .937 | 23 | .157 |
|  | RLH | .197 | 11 | .200^*^ | .946 | 11 | .596 |
|  | SBL | .241 | 15 | .019 | .799 | 15 | .004 |
| δ^15^N (‰) | CSM | .100 | 54 | .200^*^ | .950 | 54 | .026 |
|  | HGM | .137 | 31 | .148 | .892 | 31 | .004 |
|  | FEW | .274 | 7 | .121 | .824 | 7 | .070 |
|  | SQC | .178 | 32 | .011 | .896 | 32 | .005 |
|  | STGC | .138 | 9 | .200^*^ | .924 | 9 | .431 |
|  | VGL | .385 | 3 | . | .750 | 3 | .000 |
|  | ROM | .139 | 21 | .200^*^ | .953 | 21 | .387 |
|  | QCS | .188 | 10 | .200^*^ | .972 | 10 | .908 |
|  | SBK | .122 | 23 | .200^*^ | .955 | 23 | .370 |
|  | RLH | .118 | 11 | .200^*^ | .982 | 11 | .977 |
|  | SBL | .125 | 15 | .200^*^ | .970 | 15 | .863 |
| δ^13^C_carb_ (‰) | CSM | .100 | 54 | .200^*^ | .969 | 54 | .182 |
|  | HGM | .102 | 31 | .200^*^ | .975 | 31 | .659 |
|  | FEW | .184 | 7 | .200^*^ | .944 | 7 | .679 |
|  | SQC | .114 | 32 | .200^*^ | .953 | 32 | .173 |
|  | STGC | .130 | 9 | .200^*^ | .969 | 9 | .889 |
|  | VGL | .321 | 3 | . | .881 | 3 | .328 |
|  | ROM | .091 | 21 | .200^*^ | .972 | 21 | .787 |
|  | QCS | .240 | 10 | .109 | .745 | 10 | .003 |
|  | SBK | .185 | 23 | .040 | .839 | 23 | .002 |
|  | RLH | .136 | 11 | .200^*^ | .947 | 11 | .604 |
|  | SBL | .159 | 15 | .200^*^ | .936 | 15 | .340 |
|  | *. This is a lower bound of the true significance. | | | | | | |
|  | a. Lilliefors Significance Correction | | | | | | |

H_0_: The sample comes from a normal distribution

H_1_: The sample does not come from a normal distribution

- Where P-value is smaller than 0.05, the null hypothesis is rejected
- Some sites do not come from a normal population so nonparametric tests will be used for comparisons
